# The genomic response of human granulosa cells (KGN) to melatonin and specific agonists/antagonists to the melatonin receptors

**DOI:** 10.1038/s41598-022-21162-y

**Published:** 2022-10-20

**Authors:** Asma Arjoune, Marc-André Sirard

**Affiliations:** 1 Centre de recherche en Reproduction, Développement et Santé Intergénérationnelle, Faculté des Sciences de L’agriculture et de l’alimentation, Département des Sciences animales, Université Laval, Québec, QC G1V 0A6 Canada; 2 Department of Animal Production, National Agronomic Institute of Tunisia, University of Carthage, 43 Avenue Charles Nicolle, 1082 Mahrajène, Tunisia

**Keywords:** Cell biology, Endocrinology

## Abstract

Melatonin is a known modulator of follicle development; it acts through several molecular cascades via binding to its two specific receptors MT1 and MT2. Even though it is believed that melatonin can modulate granulosa cell (GC) functions, there is still limited knowledge of how it can act in human GC through MT1 and MT2 and which one is more implicated in the effects of melatonin on the metabolic processes in the dominant follicle. To better characterize the roles of these receptors on the effects of melatonin on follicular development, human granulosa-like tumor cells (KGN) were treated with specific melatonin receptor agonists and antagonists, and gene expression was analyzed with RNA-seq technology. Following appropriate normalization and the application of a fold change cut-off of 1.5 (FC 1.5, *p* ≤ 0.05) for each treatment, lists of the principal differentially expressed genes (DEGs) are generated. Analysis of major upstream regulators suggested that the MT1 receptor may be involved in the melatonin antiproliferative effect by reprogramming the metabolism of human GC by activating the PKB signaling pathway. Our data suggest that melatonin may act complementary through both MT1 and MT2 receptors to modulate human GC steroidogenesis, proliferation, and differentiation. However, MT2 receptors may be the ones implicated in transducing the effects of melatonin on the prevention of GC luteinization and follicle atresia at the antral follicular stage through stimulating the PKA pathway.

## Introduction

Ovarian follicular development requires close coordination between the oocyte and the surrounding somatic cells such as granulosa cells (GC). The major roles of GC include the synthesis of sex steroids and of several growth factors that interact with the oocyte during folliculogenesis^1^. The direct communication as well as the paracrine interactions between the oocyte and GC provide essential metabolic support for oocyte development and maturation^2^. This bidirectional communication creates an intrafollicular microenvironment sensitive to the female metabolic context which can affect the oocyte epigenetic and metabolic programming throughout follicular growth^3^. The shift in energy metabolism to glycolysis in GC during follicular growth prepares for the proliferation and differentiation processes^4^. Therefore, cultured ovarian GC are a useful model to investigate the molecular mechanisms of gene regulation related to metabolic processes in a dominant follicular stage context.

Melatonin, which is normally generated in the brain but can also be secreted by GC, is a known modulator of follicle development, oocyte maturation, and embryo development^5–7^. It acts through several molecular cascades via binding to its two high-affinity, G-protein coupled receptors: MTNR1A and MTNR1B, also named MT1 and MT2, respectively^8,9^. Melatonin also protects the integrity of oocytes and GC by scavenging reactive oxygen species (ROS) and regulating apoptosis-related genes to prevent apoptosis^10,11^.

Recently, the supplementation of porcine oocyte culture media with melatonin was shown to affect oocyte lipid metabolism and mitochondrial functions, but the underlying processes were not identified^12,13^. Furthermore, our recent results suggested that melatonin could be a modulator of human GC metabolism at the dominant follicular stage via the PKB/mTOR signaling pathway to prevent follicular atresia and early luteinisation at the antral stage^14^.

In this study, KGN cells were used as a model to better characterize the roles of the MT1 and MT2 receptors on the effects of melatonin on follicular atresia and the regulation of proliferation and differentiation of granulosa cells during the antral stage. The KGN cell line originated from a diagnosed stage-3 granulosa cell tumor from an antral or pre-antral stage follicle of a Japanese woman in 1984 and are characterized by functional FSH receptors and other similarities with in vivo ovarian granulosa cells^15^. Accordingly, KGN cells were treated with specific melatonin receptor agonists and antagonists and the results of RNA seq technology and genomic analysis served to describe the roles of MT1 and MT2 in human GC and to identify which one is the major receptor implicated in the effects of melatonin on the metabolic processes in the dominant follicle.

## Results

### RNAseq data analysis

#### Gene expression analysis

Transcriptome analysis of KGN cells following six different treatments (control and melatonin alone, or with specific MT1 or MT2 agonists or antagonists) was carried out by RNA seq technology. Treatments were compared to the appropriate control group using pairwise comparison following standardization and the application of differential cut-offs for each treatment. The lists of the principal differentially expressed genes (DEGs) from these contrasts are included as supplemental data (Supplemental table S1, S2, S3, S4, S5 and S6).

To characterize the principal upstream regulators and genomic pathways associated with the observed DEGs in each treatment, functional analysis was executed with the Ingenuity Pathway Analysis (IPA) software (Qiagen, Mississauga, ON, Canada) which can analyze gene expression patterns using a built-in scientific literature-based database^16^. The combination of our dataset and the IPA knowledge extracted from the literature facilitates the interpretation of the transcriptional changes resulting from the treatments and allows the identification of the upstream regulators and biological functions modulated by the treatments.

#### Analysis of upstream regulators

The IPA software computes two statistical measures for each potential transcription regulator: an overlap p-value and an activation z score. The overlap p‐value calls likely upstream regulators based on significant overlap between the genes in the dataset and known targets regulated by a transcription regulator. The overlap p-value was calculated in our analysis with the Fisher’s Exact Test. The z score (activation, z-score ≥ 2 or inhibition, z-score ≤  − 2) was identified by comparing the expression of genes in our dataset to expected results from the literature. The biological insights implied from the observed gene expression in each treatment are highlighted below.

The IPA upstream analysis revealed a list of upstream regulators associated with the differentially expressed genes in each treatment (Supplemental table 7). For the MLT (10^–7^ M) treatment, the list of upstream regulators contained transcription regulators, transporters, enzymes, and growth factors. Histone Deacetylase 4 (HDAC4), a class IIa histone deacetylase, the most significant regulator in this treatment, was involved in regulation of chicken skeletal muscle cell proliferation, differentiation, and apoptosis^17^. The Krüppel-like zinc-finger transcription factor Ikzf1 is critical for B and T cells development and is involved in the inhibition of glucose transporter expression^18,19^. Autophagy Related 12 (ATG12), another significant regulator in the MLT (10^−7^ M) treatment, is a positive regulator of autophagy in granulosa cells of numerous species^20,21^. The HCR gene, which encodes the Coiled-Coil α- Helical Rod protein 1 (CCHCR1), was also identified as a significant regulator in this treatment. It is involved in epithelial cell proliferation and differentiation^22^, and steroidogenesis^23^. Inhibin A (INHA) was one of the most significant regulators in response to the MLT (10^−7^ M) treatment and is a potent growth factor involved in porcine follicle development^24^.

The response of KGN cells to the lower MLT concentration (10^–9^ M) treatment included transcription factors, enzymes, cytokines, and chemical drugs (Supplemental table 7). The most significant regulator in this treatment, LDL-cholesterol (low density lipoprotein), is the main cholesterol transport vehicle in mammalian cells and a potential source of cholesterol for steroid synthesis in granulosa cells. Factor 18 (SOX18), a member of the Sry-related HMG box-containing (SOX) family, is an important factor for vascular development^25^. Heparan Sulfate Proteoglycan 2 (HSPG2), also named perlecan, controls the regulation of the extracellular matrix in ovarian follicles^26^. Another cell-cycle and cell survival factor, the Nuclear Receptor Binding SET Domain Protein 2 (NSD2), also known as Wolf-Hirschhorn syndrome candidate 1 (WHSC1), was also a significant regulator^27,28^. NQO2 N-Ribosyldihydronicotinamide Quinone Reductase 2 (NQO2), is an enzyme that works in a context dependent way, and is a ROS generator and detoxifier^29^. NQO2 contains a melatonin binding site, known as melatonin receptor 3 (MT3)^30^.

The list of upstream regulators identified in response to the MT1 agonist N-acetyl serotonin (NAS) used in combination with melatonin (10^−9^ M) is available in Supplemental table 7. Microfibrillar-Associated Protein 5 (MFAP5), which is downstream of Akt, downregulates inflammation and controls cell proliferation and progression, had a tendency to be inhibited^31–33^. Another factor downstream of Akt, PDZ Domain Containing 1 (PDZK1), also known as Na^+^/H + Exchanger Regulator Factor-3 (NHERF3) was identified as an upstream regulator. It is a cell cycle regulator, and its expression correlated with Akt activation in the MCF-7 breast cancer cell line^34,35^. Caveolin-1 (CAV1), another significant regulator identified in the NAS treatment, facilitates endocytosis and regulates cell metabolism such as modulation of glycolytic activities, mitochondrial functions, fatty acid metabolism, cholesterol distribution, and insulin signaling in normal and cancer cells^36,37^. MicroRNA-30 (mir-30), which is an important member of the miRNA family, was reported as an inhibitor in ovarian cancer^38^, a pro-inflammatory agonist, and a regulator of mitochondrial respiration^39,40^.

The IPA functional analysis generated another list of upstream regulators for the S26131 treatment, an agonist specific for MT1 (Supplemental table 7) and cyclin B was the most significant regulator in this treatment. Cyclin B is implicated in granulosa cell proliferation and survival^41,42^ and cooperates with RSF1 (Remodeling and Spacing Factor 1), a transcription regulator and another significant regulator, to facilitate DNA repair and maintain cell homeostasis^43^. Transforming Growth Factor Beta 2 (TGFB2) belongs to the TGF superfamily of proteins and was predicted as inhibited in this treatment (z = − 2.208, P = 1.60E−03). It is known for its antiproliferative and autophagic action in both bovine and porcine granulosa cells, but it is also implicated in ovarian follicular differentiation^44–46^. Protein Kinase D 1 (PRKD1, also named PKD1), is an upstream kinase for p38 MAPK. It was identified as a significant upstream regulator in our data with a tendency to be inhibited by the MT1 antagonist S26131 (z = − 0.192, P = 1.92E−03). MicroRNA 8 (mir-8), also known as mir-200 in humans, was identified as activated in the S26131 treatment (z = 2.230, P = 2.80E−03). It controls cell growth through insulin signaling and PI3K activation in different tissue types and maintains cell survival^47,48^.

The MT2 specific agonist IIK7 was used to investigate the role of MT2 at the antral follicular stage. The Zinc Finger and BTB Domain Containing 17, (ZBTB17, also known as MIZ-1), which is involved in c-MYC regulation, was the most significant upstream regulator in response to this treatment (Supplemental table 7). The E2F transcription factor 4 (E2F4), one of the major regulators of cell proliferation^49^, was predicted to be activated in this treatment (z = 2.000, P = 5.77E−26). Cytoskeleton Associated Protein 2 Like (CKAP2L), another potent regulator of cell proliferation and growth^50^, was predicted as inhibited (z = − 4.796, P = 1.19E−23). Strangely, CDKN1A (Cyclin Dependent Kinase Inhibitor 1A), a cell cycle inhibitor which promotes the final differentiation of GC, was suggested to be activated in this treatment (z = 3 219, P = 6,36E−23). The CCAAT/Enhancer-Binding Protein Beta (CEBPB), a key hepatocyte transcription factor implicated in cell cycle and epidermal differentiation^51^, was predicted to be inhibited (z = − 4.275, P = 3.72E−21). Prostaglandin E Receptor 2 (PTGER2) was also suggested as an inhibited regulator in response to the IIk7 treatment (z = − 4.487, P = 1.66E−16). The cell cycle inhibitor TP53, which is also involved in granulosa cell differentiation and apoptosis, was predicted to be activated in the IIK7 treatment (z = 4.700, P = 6.47E−14).

The list of upstream regulators identified in response to the 4P-PDOT (MT2 specific antagonist) treatment is presented in supplemental table 7. Prokineticin Receptor 2 (PROKR2), a G protein receptor that activates the MAPK and STAT signaling pathways, was the most significant regulator in this treatment. Immunoglobulin Superfamily Containing Leucine Rich Repeat (ISLR), a significant regulator in this treatment, was previously implicated in cell proliferation^52^ and prevented autophagy in the skeletal muscle. One of the most significant upstream regulators, YAP1 (Yes-Associated Protein), was implicated in cell proliferation, differentiation, and apoptosis^53^. Ribosomal Protein S15 (RPS15), a significant regulator in the 4P-PDOT treatment, is a regulator of ribosome biogenesis and mRNA translation that maintains cell senescence^54^.

### Major canonical pathways related to melatonin and melatonin receptor agonist and antagonist treatments

The lists of DEGs in response to each of the treatments were downloaded from the IPA software separately and were analyzed to identify the most significant canonical pathways in our datasets (Supplemental table 8). The most significant canonical pathways following activation of MT1 receptors by NAS and MLT were the nNOS Signaling in Neurons (p = 1.54E−03), Cell Cycle: G2/M DNA Damage Checkpoint Regulation (p = 1.84E−03), and The Visual Cycle (p = 5.05E−03). After inhibition of MT1 receptors by S26131 (in the presence of melatonin), Semaphorin Neuronal Repulsive Signaling Pathway (p = 3.84E−03) and Ephrin A Signaling (p = 4.07E−03) were the most significant canonical pathways. The most significant canonical pathways following the activation of MT2 receptors by IIK7 in the presence of melatonin were the Kinetochore Metaphase Signaling Pathway (p = 5.23E−10), Mitotic Roles of Polo-Like Kinase (p = 2,55E-08), and Cell Cycle: G2/M DNA Damage Checkpoint Regulation (p = 1.46E−07). Following MT2 receptor inhibition with 4P-PDOT (in the presence of melatonin), the most significant canonical pathways were Spermine Biosynthesis (p = 9.59E−03), ATM Signaling (p = 1.15E−02), and Oxidized GTP and dGTP Detoxification (p = 1.91E−02).

## Discussion

This study revealed, for the first time, the potential impact of extracellular melatonin on the transcriptome of human granulosa cells. Our analysis allowed us to distinguish the specific receptor effects from the general antioxidant effects of melatonin. To the best of our knowledge, our study is the first to investigate the implication of MT1 and MT2 in the response of human GC to melatonin and to identify the signaling cascades involved. The analysis of the transcriptomic response identified genes and pathways related to steroidogenesis, cell proliferation and differentiation, cell survival, apoptosis, inflammation, oxidative stress, and energy metabolism. Likewise, our results revealed that in addition to stimulating the PKB/mTOR pathway, melatonin may activate the PKA pathway via the MT2 receptor to control human GC differentiation and to prevent atresia and early aging during follicle development.

The mRNAs for the MT1 and MT2 melatonin receptors were previously detected in human granulosa cells^55^, and activation of these receptors may induce different second messengers depending on cell types, doses, and species^56^. Activation of MT1 and MT2 may decrease cAMP accumulation and consequently suppress protein kinase A (PKA) activity in different tissues^57–59^. Furthermore, our previous results suggested that melatonin can prevent aging and early luteinization of human GC at the antral follicular stage via the PKB/mTOR signaling pathway^14^. Thus, melatonin influences GC functions via numerous mechanisms. However, there is a little information on the roles of MT1 and MT2 in human GC during folliculogenesis, as well as on whether these receptors affect the response of human GC to melatonin. In this study, we used KGN cells as a specific in vitro model of human GC at the antral stage to examine the physiological effects of MT1 and MT2 activation or knockdown on GC functions and to predict which signaling pathways are implicated in the responses of metabolic processes to melatonin in the dominant follicle.

### Effects of MT1 receptor activation or inhibition on human GC functions

We investigated the effects of MT1 activation or knockdown during melatonin supplementation on the maintenance of ovarian GC functions during follicle development and growth. Treatment of human granulosa cells (KGN) with melatonin (10^−9^ M) revealed that melatonin may sustain granulosa cells steroidogenesis by regulating the expression of LDL which was the most significant regulator in our data. LDL are formed from triglyceride-rich lipoproteins, are the main cholesterol transporter^60^, and are indispensable for progesterone production in cultured human GC^61^. Progesterone can be converted to 17β-estradiol in follicles to support growth and ovulation^62^. Melatonin is also known for its anti-apoptotic and anti-mitogenic actions in rat granulosa cells^63^. Melatonin supplementation of human GC may promote follicle vascularization through SOX18 and VEGFC (Vascular Endothelial Growth Factor C) gene expression according to the results of this study. The SOX family comprises genes with a conserved high-mobility group DNA-binding domain (HMG box) homologous to SRY that controls cell proliferation and differentiation during embryogenesis and development^25,64^. Vascularisation is highest in the dominant follicle in most species and supplies GC with oxygen, growth factors, gonadotropins, lipids, and steroid precursors required for antral follicle growth, dominance, and oocyte maturation^65,66^. Maintenance of vascular permeability is linked to follicle survival as well as oocyte competence, and vascular loss is a sign of the earlier stages of follicular atresia^67,68^. Xiaowei and al., 2017, suggested that AMPK inhibition intensifies follicle vascularization and promotes follicle growth in mammalian ovaries^69^. Melatonin may regulate follicle development by maintaining GC proliferation and vascular permeability through AMPK inhibition at the antral follicular stage. Additionally, melatonin may control the formation of the extracellular matrix (ECM) in human GC by promoting the expression of ECM genes such as the Perlecan (HSPG2) gene which encodes one of the ECM component proteins expressed later in granulosa cells of antral follicles^70,71^. The ECM regulates many cellular processes during folliculogenesis by acting as a reservoir for autocrine and paracrine signaling^72^. It affects cell attachment, communication, and steroid production in cultured granulosa cells^73^ of numerous mammalian species. The survival and proliferation of ovine granulosa cells in culture can be influenced by the ECM composition^74,75^ which is also implicated in GC differentiation after ovulation^76^. Our previous data revealed that melatonin maintains cell proliferation and survival in KGN cells and modulates GC metabolism^14^. Other important factors involved in cell cycle and metabolism reprogramming were highlighted by the upstream effect of NSD2 which plays a fundamental role in cell development. It induces the expression of cell cycle progression-associated genes in normal and cancer cells^77,78^ and its loss induces cell senescence and contributes to the remodeling of mitochondrial activities^79^. Nevertheless, studies revealed that NSD2 can modulate the expression of key glucose metabolic enzymes, it increases NADPH production by increasing the carbon flux through the Phosphate Pentose Pathway (PPP) which in turn is used to reduce ROS production and to maintain cell survival and redox homeostasis^80^. This enzyme is also involved in cell proliferation and insulin secretion in pancreatic β cells^81^. The role of melatonin as an efficient ROS scavenger is well documented, and according to our data, melatonin may control redox homeostasis by regulating the expression of the NQO2 enzyme which is also a melatonin cytosolic receptor (MT3)^82^. Its antiproliferative effect in response to melatonin was observed in tumour cells such as HepG2 cells^83^; however, it maintained the proliferation of vascular smooth muscle cells through ERK1/2 phosphorylation^84^. Our data suggest that melatonin maintains GC functions and prevents oxidative stress by modulating metabolism.

After treatment with N-acetyl serotonin (NAS), an agonist of the MT1 receptor, transcriptome analysis by IPA generated a list of upstream regulators and most of them, as for the melatonin treatment, were related to cell cycle and differentiation. The Akt pathway is downstream of some of these regulators such as MFAP5, PDZK1, and CAV1, which are related to cell progression and growth. The expression of MFAP5 also affected inflammatory related genes in previous studies^31,33,35^, and its inhibition in our data by NAS suggests that MT1 may be responsible for limiting GC proliferation and growth, and at the same time preventing early luteinization at the antral follicular stage through activation of the Akt pathway.

Our results suggest that the MT1 receptor may play an essential role in modulating the metabolic response to melatonin in human GC by enhancing Caveolin-1 (CAV1) and activating SPARC (Secreted Protein Acidic And Cysteine Rich expression) gene expression. Cav1 participates in numerous differentiation processes^85,86^, cell survival, and senescence by maintaining Akt activation^87^. In addition to its master role in cellular processes^88^, Cav1 was also described as an energy homeostasis regulator. Furthermore, Cav1 increased glucose uptake and stimulated the transcription of glucose transporters to produce ATP through the glycolytic pathway in cancer and normal cells. It may regulate mitochondrial function by regulating cholesterol flux in mouse embryonic fibroblasts^36^. It is also involved in fatty acid transport and insulin secretion in pancreatic beta cells^89^. Our data suggest that the MT1 receptor may be involved in the melatonin antiproliferative effect by reprogramming the metabolism of human GC through the modulation of mitochondrial function and of the glycolytic pathway.

**Figure 1:**
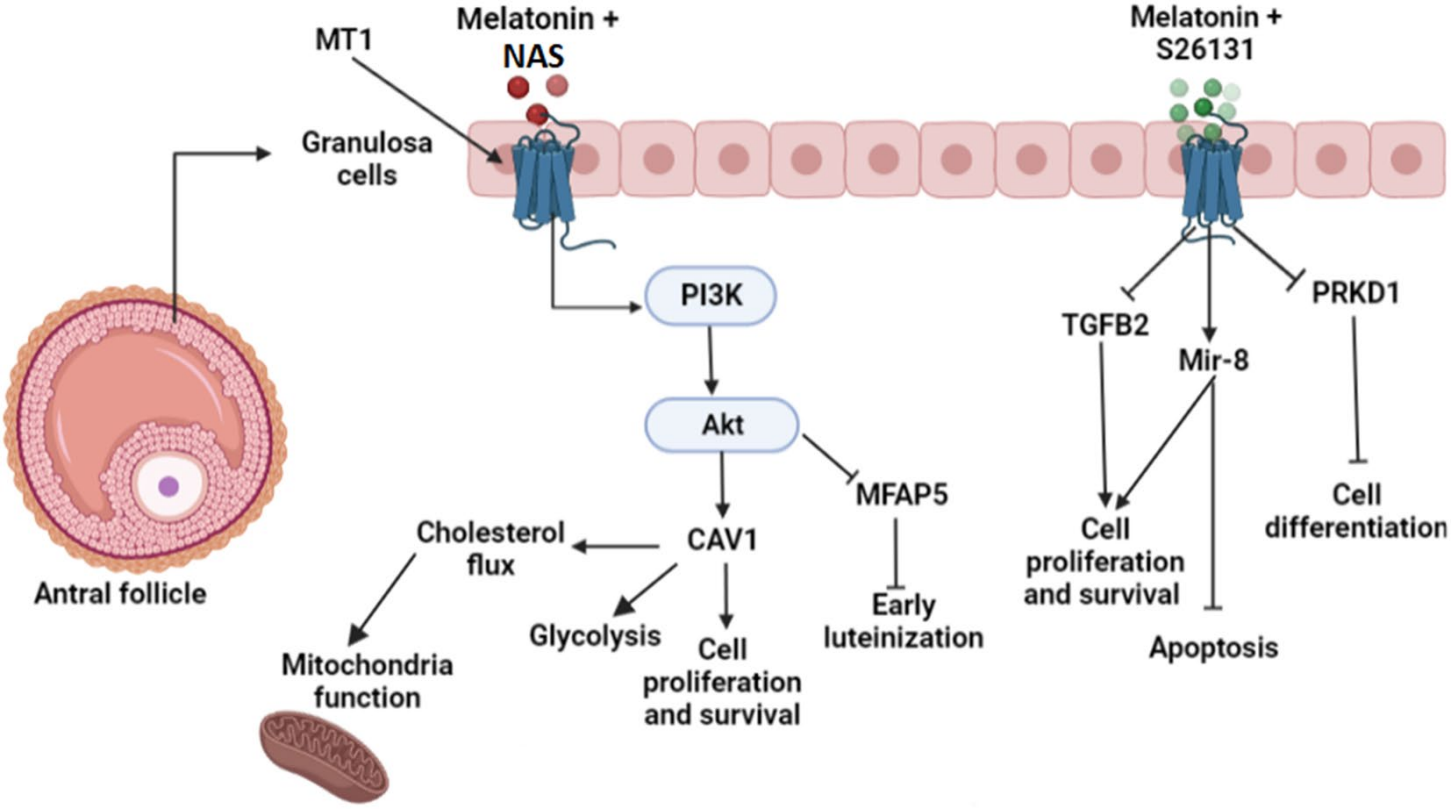
Effects of MT1 receptor activation or knockdown on the response of human granulosa cells to melatonin (generated by BioRender, https://biorender.com/).

Furthermore, our results suggest that blocking the MT1 receptor with S26131 impacted the expression of some cell cycle, proliferation, and survival-related genes. Among the upstream regulators strongly inhibited in response to the MT1 antagonist and related to cell differentiation was Transforming Growth Factor Beta 2 (TGFB2), which is involved in follicle growth and granulosa cell proliferation^90,91^. This growth factor had only a mild stimulatory or even an inhibitory effect on GC proliferation and survival in numerous species^45,92,93^. Its inhibition in our data by S26131 annuls its antiproliferative effect on GC. It appears that melatonin maintains human GC proliferation and differentiation through a receptor other than MT1, regardless of the role of MT1 (Fig. 1). Furthermore, previous studies revealed that melatonin promoted osteoblastic cell differentiation via upregulating the p38 MAPK and PRKD1 signaling pathways^94^. However, MT1 receptor knockdown in our melatonin treatment may have inhibited the melatonin effect on stimulation of GC differentiation by slightly reducing PRKD1 expression. The MT1 antagonist did not block the effects of melatonin on the maintenance of GC proliferation as the expression of the miR-8 gene was identified as an activated regulator by IPA upstream analysis (z = 2.230, P = 2.80E−03). The miR-8 gene is part of the miR-200 family in humans, and members of this family play essential roles in neuron differentiation and proliferation^95,96^, cell apoptosis^97^, and the control of body fat via the insulin signaling pathway^47^. The addition of S26131 to the melatonin treatment did not mask the proliferative and survival effects of melatonin in human GC, and this fact led us to believe that, regardless of the role of the MT1 receptor in GC, the MT2 receptor may be more involved in the response of human GC to melatonin supplementation at the antral follicular stage. On the other hand, several studies found that melatonin promoted viability, proliferation, and steroidogenesis in bovine and mouse GC via its specific receptor MT1^98^.

### Effects of MT2 receptor activation or inhibition on human GC functions

The role of the MT2 receptor on female fertility in response to melatonin is well established and this receptor may be more implicated in promoting GC functions. We used the specific agonist IIK7 to understand the consequences of MT2 receptor activation in human GC. Surprisingly, analysis of the transcriptomic response of KGN cells to IIK7 in combination with melatonin revealed a list of upstream regulators similar to the list generated from the analysis of data from PKA activation with forskolin in KGN cells in our recent study^14^. Most of the upstream regulators identified following MT2 receptor activation were cell cycle related genes such as E2F4, CKAP2L, RABL6, CCND1, E2F1, and COPS5 which were mostly inhibited in human GC. Our data suggest that the MT2 receptor may be the one responsible for transducing the antiproliferative action of melatonin in GC at the antral follicular stage. Prostaglandin E Receptor 2 (PTGER2) was a significantly and strongly inhibited upstream factor in the IIK7 treatment (z = − 4.487, P = 1.66E−16). Strangely, PTGER2 is an adenylate cyclase-coupled receptor that increases cAMP and protein kinase A signaling and is implicated in follicle rupture, ovulation, and fertilization processes^99^. In human granulosa-lutein cells, PTGER2 mediates the effects of prostaglandins on progesterone production^100^. Through its specific MT2 receptor, melatonin may prevent premature differentiation of granulosa cells by supressing progesterone production and stimulating fibroblast growth factor 2 (FGF2), a well-known luteal proangiogenic factor which is stimulated by the elevated PGE2 levels during the periovulatory period in bovine granulosa cell^101^.

The cell cycle inhibitors tumor protein 53 (TP53) and cyclin dependent kinase inhibitor 1A (CDKN1A) were among the most significant upstream regulators identified by IPA and were strongly activated by MT2 receptor activation in the melatonin treatment (z = 4.700, P = 6.47E−14 and z = 3.219, P = 6.36E−23, respectively). The TP53 is a potent cell cycle arrest, apoptosis, and senescence factor which was also reported to be a metabolic regulator through the maintenance of mitochondrial functions especially oxidative phosphorylation to promote fatty acid β-oxidation^102^. Sirotkin and al., 2008, mentioned that TP53 is implicated in GC steroidogenesis and follicle atresia^103^. It was also identified as an activated upstream regulator in response to PKB and PKC activation in granulosa cells from follicles at the plateau phase with a reduced cell growth rate^104–106^. The essential proangiogenic factor for dominant growth and development vascular endothelial growth factor (VEGF) was also identified as a significant inhibited upstream regulator in the IIK7 treatment. Decreasing VEGF expression downregulated the PI3K/Akt pathway and reduced glucose uptake and glycolysis in peripheral organs^107^. It reinforces FSH effects in inducing the proliferation and survival of bovine and porcine granulosa cells through the ERK1/2 pathway, and in maintaining follicular cell steroidogenesis at a later stage of follicle differentiation^108,109^.

**Figure 2:**
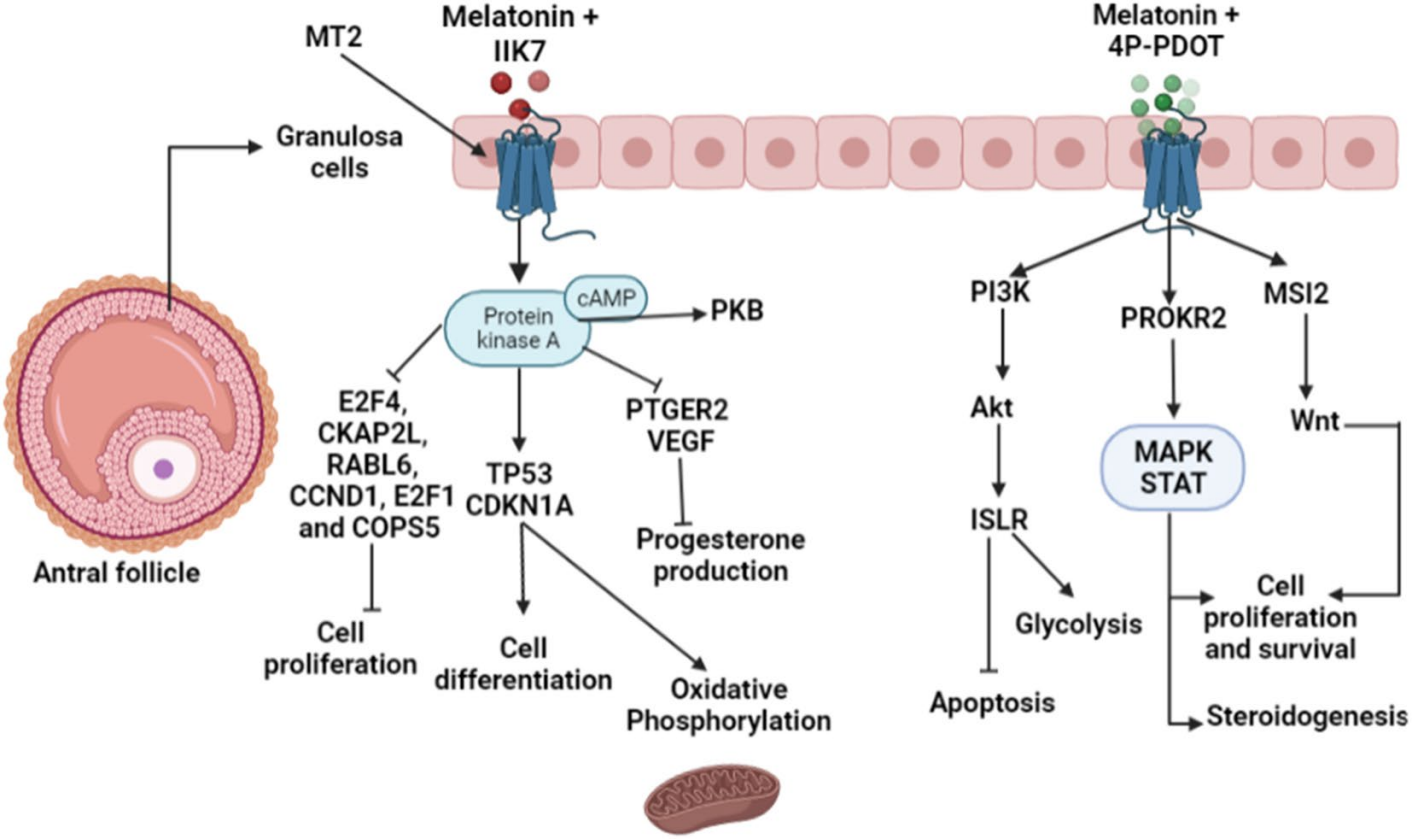
Effects of MT2 receptor activation or knockdown on the response of human granulosa cells to melatonin (generated by BioRender, https://biorender.com/).

Taken together, our results suggest that melatonin may act through the MT2 receptor to stimulate cAMP accumulation and activate the PKA signaling pathway to keep human GC growth in the deceleration phase at the antral follicular stage and to prevent early luteinization (Fig. 2). Nevertheless, PKA is a master kinase in granulosa cell proliferation and differentiation while PKB acts as a permissive pathway to PKA to maintain cell survival^110,111^. In agreement with our results, p38 mitogen-activated protein kinase (MAPK14) and forkhead box O1 (FOXO1), which are involved in the PKB pathway, were also identified as predicted regulators^112,113^. In fact, the FOXO1 protein was inhibited following PKB activation. Crosstalk between PKA and PKB in KGN cells was previously described^106^.

In line with our findings, some studies also reported that the MT2 receptor was more implicated in the GC response to melatonin in several species. He and al., 2017, found that melatonin modulated porcine GCs steroidogenesis, proliferation, and apoptosis through the MT2 receptor when the MT2 specific agonist IIK7 was used^114^. Besides, a recent study revealed that melatonin may protect porcine GC from hypoxia-induced apoptosis through reducing ROS production via activation of the MT2 –PKA–Caspase8/9 pathway^115^. In bovine GC, the role of the MT2 receptor on the effect of melatonin was highlighted as MT2 silencing inhibited GC steroids secretion, increased apoptosis related genes expression, and disrupted some folliculogenesis regulating factors^116^.

In our data, MT2 inhibition by its specific antagonist 4P-PDOT may have concealed some of the effects of melatonin on human GC (Fig. 2). Prokineticin receptor 2 (PROKR2), the most significant upstream regulator in this treatment, is known for its ability to activate the MAPK and STAT pathways which play central roles in GC proliferation, survival, and steroidogenesis^42,117^. The expression of the immunoglobulin superfamily containing leucine repeat (ISLR) gene was also highlighted in our data. It was previously suggested that ISLR may modulate glycolysis in human NSCLC cells by activating the IL-6/JAK/STAT3 signalling pathway^118^. It is involved in myoblast differentiation and can alleviate skeletal muscle atrophy and prevent muscle cell apoptosis via the IGF/PI3K/AKT-FOXO signaling pathway^119^. Musashi RNA binding protein 2 (MSI2), a significant upstream in the 4P-PDOT treatment, plays essential roles in many cellular processes by activating the Wnt pathway^120^. Researchers have shown that the two MSI proteins play critical roles in the proliferation and differentiation of stem cells of the hematopoietic and nervous systems^121,122^. Nonetheless, Wang and al., 2021 confirmed that MT2 inactivation in bovine GC by 4P-PDOT may affect the regulatory action of melatonin on GC functions^123^.

**Figure 3:**
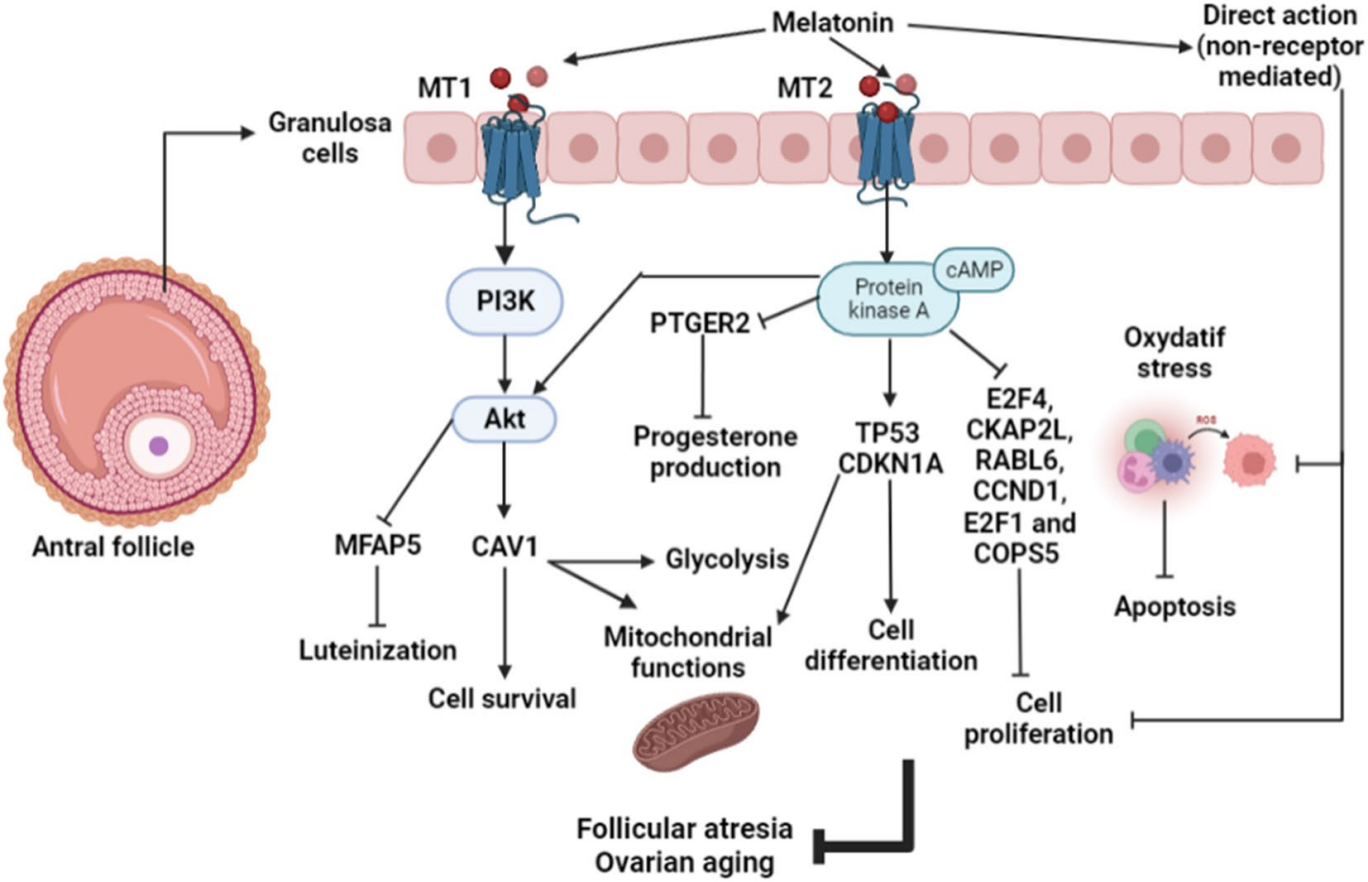
Role of MT1 and MT2 receptors on the genomic response of human granulosa cells to melatonin (generated by BioRender, https://biorender.com/).

Our findings led us to the conclusion that melatonin may modulate GC functions via MT1 receptors and probably through other nuclear receptors which exert their effects on human GC by activating PKB and other signaling cascades involved in maintaining cellular processes. Our data suggest that melatonin may act, in a complementary manner, through both MT1 and MT2 receptors to modulate human GC steroidogenesis, proliferation, and differentiation. However, MT2 receptors may be the ones implicated in transducing the effects of melatonin on the prevention of GC luteinization and follicle atresia at the antral follicular stage (Fig. 3).

## Methods

### Reagents

Chemicals such as 4-androstene-3,17-dione (product A9630), melatonin (MLT, product M5250), N-acetyl serotonin (NAS, product A1824), and 4-phenyl-2-propionamidotetralin (4P-PDOT, product SML1189) were purchased from Sigma-Aldrich (St. Louis, MO, USA). IIK7 (product sc-300823A) and S26131 were purchased from Santa Cruz Biotechnology (Texas, USA) and Medkoo Biosciences (Morrisville, NC, USA) respectively. To make stock solution, MLT and NAS were dissolved in 95% ethanol; 4P-PDOT, IIK7, and S26131 were dissolved in 95% DMSO.

### Human granulosa-like tumour cell line (KGN)

The KGN human granulosa-like tumour cell line, which retains properties of normal granulosa cells such as cAMP-inducible aromatase expression and steroids production^15^, was purchased from the RIKEN Bioresource Centre (Tsukuba, Japan). A research ethics board (REB) review was not required for working with the KGN cell line according to the Human Research Ethics Committee of Laval University (CÉRUL).

### Cell culture and treatments

The KGN cells were cultured as previously described^106^. Cells were cultured in DMEM/F12 medium (Life Technologies, Waltham, MA, USA) supplemented with 10% foetal bovine serum (Corning, NY, USA) and 100X Penicillin–Streptomycin (10,000 U/mL) (Life technologies) in an atmosphere of 5% CO_2_/95% O_2_ at 37 °C. The cells were thawed on day 1, placed in a cell culture flask (75 cm^3^, Sigma-Aldrich), and sub-cultured on day 3. Confluence was never over 80%. On day 6, cells were placed in a 6-well dish at a density of 1.5 $\times$ 10^5^ cells per well in 1.5 mL of medium and grown for 72 h at which point they were reaching almost full confluency. The medium was then replaced with charcoal-stripped (SVFA) DMEM/F12 containing antibiotics and 100 nmol L^−1^ of 4-androstene-3,17-dione for all experimental conditions.

To study the mechanisms of action of melatonin dependent on the MT1 and MT2 receptors, the cells were cultured for 24 h in the presence of melatonin alone (10^–9^ M), melatonin (10^–9^ M) plus a MT1 specific agonist (NAS, 10^–9^ M), melatonin (10^–9^ M) plus a specific MT1 antagonist (S26131, 10^–9^ M), melatonin (10^–9^ M) plus a MT2 specific agonist (IIK7, 10^–9^ M), or melatonin (10^–9^ M) plus a specific MT2 antagonist (4P-PDOT, 10^–9^ M) for 24 h. The control consisted in the addition of ethanol at less than 0.05% of the final culture volume. The experiment was repeated four times (four different weeks) to generate three biological replicates for RNAseq analysis. An additional treatment consisting of melatonin at 10^–7^ M was added to assess the potential antioxidant effects of this molecule at a concentration exceeding the receptor activation level.

### RNA purification and deep sequencing

Total RNA was isolated with the AllPrep DNA/RNA Mini Kit (Qiagen, Mississauga, ON, Canada) according to the manufacturer’s instructions. Total RNA integrity and concentrations were evaluated on a 2100-Bioanalyzer (Agilent Technologies, Palo Alto, CA). The total RNA input used for sequencing was 360 ng for each treatment which consisted of 90 ng from each biological replicate. The mRNA was isolated from total RNA using the NEBNext Poly (A) mRNA Magnetic Isolation Module (E7490S; NEB).

PolyA-selected mRNA was fragmented to a mean size of 200 nt, reverse transcribed to generate double-stranded cDNA, and converted to a paired-end library using the NEBNext Ultra RNA Library Prep kit for Illumina (E7530S; NEB) according to the manufacturer’s instructions, with Agencourt AMPure XP beads and NEBNext Mutliplex Oligos for Illumina (set1, E7335S; NEB). Libraries were sent to the Génome Québec Innovation Centre at McGill University for quality control tests. Libraries were then pooled at equimolar concentrations and sequenced on an Illumina HiSeq4000 in paired-end mode with 100 base pair reads (PE100) to a depth between 55 and 67 million reads.

### Transcriptome assembly and expression level estimate from read counts

Ensembl (release 91) was used as the source of annotated genes and transcript isoforms. Trimming of adapters was performed using Trimmomatic. Sequencing adapters were removed, and base calls with a quality score below 30 were removed from the end of the reads^124^. Trimming was performed with the minimal length set at 32 nt and these reads were kept for further processing. Pseudoalignment of all transcripts described in release 91 of ENSEMBL cDNA gene annotation was accomplished by the kallisto tool^125^. Differential expression of genes was then assessed using pairwise comparisons in EdgeR^126^. Because of the lack of technical replicates, dispersion within EdgeR was evaluated by grouping samples for similar comparisons (first for control, then for MLT, MLT with NAS, MLT with S26131, MLT with IIK7, and MLT with 4P-PDOT samples) and dropping the factor with the lowest impact, as suggested by the EdgeR manual.

According to the Transcript Support Level (TSL) used by the Ensembl gene annotation system, normalized data from RNAseq analysis should first be sorted for significance and then filtered to highlight the well supported and poorly supported transcript models which rely on the comparison of the mRNA and EST alignments to the GENCODE transcripts. The transcripts are scored according to how well the alignment matches over its full length. Differentially expressed genes selected in the present analysis were assigned to the evaluated annotation tsl1 which means that all splice junctions of the transcript were supported by at least one non-suspect mRNA.

## Supplementary Information

Supplementary Tables.

## Data Availability

The data discussed in this publication have been deposited in NCBI’s Gene Expression Omnibus (GEO) and are accessible through GEO Series accession number GSE201943.
