# Supplementary material for: The genomic response of human granulosa cells (KGN) to melatonin and specific agonists/antagonists to the melatonin receptors
Source: Sci Rep. 2022 Oct 20;12:17539. doi: 10.1038/s41598-022-21162-y (PMC9584952; doi:10.1038/s41598-022-21162-y)
Supplement: Supplementary file 1 — Supplementary Tables. [file 41598_2022_21162_MOESM1_ESM.docx]

**Appendices**

**Supplemental Table S1: List of DEGs in melatonin treatment (10^-7^ M) (FC=0, adjusted p-value ≤ 0.05)**

| Symbol | Entrez Gene Name | ID | Expr Log Ratio | Expr p-value |
| --- | --- | --- | --- | --- |
| ABHD3 | abhydrolase domain containing 3, phospholipase | ENSBTAG00000005709 | -0,258 | 4,57E-02 |
| ADAMTSL2 | ADAMTS like 2 | ENSBTAG00000007492 | 2,975 | 2,98E-02 |
| ADGRE5 | adhesion G protein-coupled receptor E5 | ENSBTAG00000021818 | 0,166 | 4,37E-02 |
| ASB1 | ankyrin repeat and SOCS box containing 1 | ENSBTAG00000003376 | 0,349 | 4,19E-02 |
| ASB18 | ankyrin repeat and SOCS box containing 18 | ENSBTAG00000002024 | 4,177 | 1,65E-02 |
| ATP6V1C2 | ATPase H+ transporting V1 subunit C2 | ENSBTAG00000001927 | -3,155 | 4,82E-02 |
| BOD1 | biorientation of chromosomes in cell division 1 | ENSBTAG00000034598 | -0,128 | 4,88E-02 |
| C17orf80 | chromosome 17 open reading frame 80 | ENSBTAG00000006089 | 0,289 | 2,51E-02 |
| C3orf18 | chromosome 3 open reading frame 18 | ENSBTAG00000010165 | 0,420 | 2,02E-02 |
| C7 | complement C7 | ENSBTAG00000011766 | -0,212 | 3,56E-02 |
| CARMIL3 | capping protein regulator and myosin 1 linker 3 | ENSBTAG00000015208 | 1,164 | 4,66E-02 |
| CASKIN2 | CASK interacting protein 2 | ENSBTAG00000007220 | 0,124 | 3,62E-02 |
| CCDC136 | coiled-coil domain containing 136 | ENSBTAG00000011002 | -0,329 | 1,95E-02 |
| CDK5RAP1 | CDK5 regulatory subunit associated protein 1 | ENSBTAG00000018535 | -0,236 | 2,36E-02 |
| CEP85 | centrosomal protein 85 | ENSBTAG00000009579 | -0,241 | 1,60E-02 |
| CHEK2 | checkpoint kinase 2 | ENSBTAG00000004956 | -0,429 | 2,47E-02 |
| CHRNA10 | cholinergic receptor nicotinic alpha 10 subunit | ENSBTAG00000055237 | -2,069 | 2,55E-02 |
| COCH | cochlin | ENSBTAG00000021844 | -0,199 | 4,96E-02 |
| CTC1 | CST telomere replication complex component 1 | ENSBTAG00000007117 | 0,535 | 4,60E-02 |
| DHH | desert hedgehog signaling molecule | ENSBTAG00000000124 | 3,522 | 3,42E-02 |
| DLL4 | delta like canonical Notch ligand 4 | ENSBTAG00000010361 | -2,029 | 1,43E-02 |
| DRC3 | dynein regulatory complex subunit 3 | ENSBTAG00000008798 | 0,398 | 5,00E-02 |
| DYNLRB2 | dynein light chain roadblock-type 2 | ENSBTAG00000017752 | 0,976 | 5,25E-03 |
| EEF1AKNMT | eEF1A lysine and N-terminal methyltransferase | ENSBTAG00000017721 | -0,126 | 3,09E-02 |
| ELF3 | E74 like ETS transcription factor 3 | ENSBTAG00000008756 | -3,870 | 4,75E-02 |
| FAM210B | family with sequence similarity 210 member B | ENSBTAG00000013007 | 0,365 | 4,50E-02 |
| FGFR3 | fibroblast growth factor receptor 3 | ENSBTAG00000007164 | 0,604 | 2,29E-02 |
| FUNDC1 | FUN14 domain containing 1 | ENSBTAG00000001637 | -0,247 | 4,27E-02 |
| GKAP1 | G kinase anchoring protein 1 | ENSBTAG00000023523 | -0,207 | 4,75E-02 |
| GLA | galactosidase alpha | ENSBTAG00000019256 | -0,213 | 3,34E-02 |
| GMPR | guanosine monophosphate reductase | ENSBTAG00000015743 | -0,218 | 3,81E-02 |
| GPR17 | G protein-coupled receptor 17 | ENSBTAG00000006261 | 1,808 | 4,96E-02 |
| HDAC4 | histone deacetylase 4 | ENSBTAG00000017764 | 0,416 | 1,69E-02 |
| HMGXB4 | HMG-box containing 4 | ENSBTAG00000010533 | 0,388 | 2,43E-02 |
| HSPA12A | heat shock protein family A (Hsp70) member 12A | ENSBTAG00000013779 | 0,279 | 2,09E-02 |
| ILDR2 | immunoglobulin like domain containing receptor 2 | ENSBTAG00000019497 | -3,806 | 4,00E-02 |
| IRAG1 | inositol 1,4,5-triphosphate receptor associated 1 | ENSBTAG00000007129 | 0,490 | 2,73E-02 |
| KCNA3 | potassium voltage-gated channel subfamily A member 3 | ENSBTAG00000047879 | -1,233 | 1,48E-02 |
| KCNIP3 | potassium voltage-gated channel interacting protein 3 | ENSBTAG00000017955 | -0,541 | 6,26E-03 |
| KRR1 | KRR1 small subunit processome component homolog | ENSBTAG00000011591 | -0,139 | 4,38E-02 |
| KRT17 | keratin 17 | ENSBTAG00000006806 | -0,780 | 2,60E-02 |
| KRTCAP3 | keratinocyte associated protein 3 | ENSBTAG00000018156 | 1,527 | 3,28E-02 |
| L3MBTL2 | L3MBTL histone methyl-lysine binding protein 2 | ENSBTAG00000030222 | -0,151 | 3,26E-02 |
| LACTB2 | lactamase beta 2 | ENSBTAG00000001808 | -0,306 | 4,23E-02 |
| LIPF | lipase F, gastric type | ENSBTAG00000006445 | 2,976 | 4,65E-02 |
| Lmo3 | LIM domain only 3 | ENSBTAG00000013541 | -1,093 | 5,82E-03 |
| LPAR2 | lysophosphatidic acid receptor 2 | ENSBTAG00000004652 | 0,615 | 4,69E-02 |
| LRRC40 | leucine rich repeat containing 40 | ENSBTAG00000008243 | -0,143 | 3,76E-02 |
| LRRC66 | leucine rich repeat containing 66 | ENSBTAG00000014599 | -2,823 | 2,77E-02 |
| MEIG1 | meiosis/spermiogenesis associated 1 | ENSBTAG00000002856 | -1,453 | 1,84E-02 |
| MEIS3 | Meis homeobox 3 | ENSBTAG00000006862 | 0,180 | 4,19E-02 |
| MPZ | myelin protein zero | ENSBTAG00000033835 | -1,129 | 7,42E-03 |
| MYPOP | Myb related transcription factor, partner of profilin | ENSBTAG00000045708 | 0,299 | 2,59E-02 |
| NADSYN1 | NAD synthetase 1 | ENSBTAG00000016470 | 0,184 | 2,31E-02 |
| NR0B1 | nuclear receptor subfamily 0 group B member 1 | ENSBTAG00000015532 | -1,201 | 4,23E-02 |
| NSG1 | neuronal vesicle trafficking associated 1 | ENSBTAG00000005711 | 0,883 | 1,94E-02 |
| OR7E24 | olfactory receptor family 7 subfamily E member 24 | ENSBTAG00000051507 | -1,272 | 4,27E-03 |
| PFDN4 | prefoldin subunit 4 | ENSBTAG00000008812 | -0,213 | 3,89E-02 |
| POC5 | POC5 centriolar protein | ENSBTAG00000007114 | 0,336 | 7,08E-03 |
| POLR1D | RNA polymerase I and III subunit D | ENSBTAG00000033252 | -0,279 | 4,30E-02 |
| PRRX2 | paired related homeobox 2 | ENSBTAG00000002936 | -2,786 | 4,61E-02 |
| RGS14 | regulator of G protein signaling 14 | ENSBTAG00000008497 | 0,270 | 2,36E-02 |
| RINT1 | RAD50 interactor 1 | ENSBTAG00000008302 | -0,197 | 3,21E-02 |
| RPL7 | ribosomal protein L7 | ENSBTAG00000020139 | -4,935 | 6,82E-03 |
| SCN3A | sodium voltage-gated channel alpha subunit 3 | ENSBTAG00000019385 | -1,566 | 1,02E-02 |
| SEC61A2 | SEC61 translocon subunit alpha 2 | ENSBTAG00000019508 | -0,230 | 4,18E-02 |
| SEPTIN1 | septin 1 | ENSBTAG00000021219 | -1,140 | 3,22E-02 |
| SHANK3 | SH3 and multiple ankyrin repeat domains 3 | ENSBTAG00000030180 | 0,305 | 4,99E-02 |
| SLC36A3 | solute carrier family 36 member 3 | ENSBTAG00000006624 | -1,139 | 2,88E-02 |
| SLC37A2 | solute carrier family 37 member 2 | ENSBTAG00000016704 | -1,679 | 1,22E-02 |
| SLC38A5 | solute carrier family 38 member 5 | ENSBTAG00000011854 | -1,847 | 6,69E-03 |
| SUPT3H | SPT3 homolog, SAGA and STAGA complex component | ENSBTAG00000032887 | -0,268 | 4,02E-03 |
| SYN1 | synapsin I | ENSBTAG00000005042 | 0,501 | 4,98E-02 |
| TMA16 | translation machinery associated 16 homolog | ENSBTAG00000012658 | -0,220 | 2,75E-02 |
| TRMT44 | tRNA methyltransferase 44 homolog | ENSBTAG00000004797 | -0,470 | 3,67E-02 |
| TSNAXIP1 | translin associated factor X interacting protein 1 | ENSBTAG00000006387 | -0,579 | 3,65E-02 |
| TTC9 | tetratricopeptide repeat domain 9 | ENSBTAG00000045604 | -1,062 | 3,47E-02 |
| VAMP8 | vesicle associated membrane protein 8 | ENSBTAG00000023997 | -1,307 | 4,37E-02 |
| VPS36 | vacuolar protein sorting 36 homolog | ENSBTAG00000004307 | 0,177 | 1,50E-02 |
| WDR74 | WD repeat domain 74 | ENSBTAG00000009491 | -0,190 | 3,82E-02 |
| ZBTB49 | zinc finger and BTB domain containing 49 | ENSBTAG00000014680 | 0,616 | 2,37E-02 |
| ZDHHC18 | zinc finger DHHC-type palmitoyltransferase 18 | ENSBTAG00000046672 | 0,155 | 3,39E-02 |
| ZFP2 | ZFP2 zinc finger protein | ENSBTAG00000004262 | 0,323 | 4,13E-02 |
| ZNF526 | zinc finger protein 526 | ENSBTAG00000020754 | 0,458 | 3,64E-02 |
| ZNF629 | zinc finger protein 629 | ENSBTAG00000026307 | 0,153 | 4,83E-02 |

**Supplemental Table S2: List of DEGs in melatonin treatment (10^-9^ M) (FC=0, adjusted p-value ≤ 0.05)**

| Symbol | Entrez Gene Name | ID | Expr Log Ratio | Expr p-value |
| --- | --- | --- | --- | --- |
| AAR2 | AAR2 splicing factor | ENSBTAG00000001645 | -0,150 | 4,69E-02 |
| ABCB5 | ATP binding cassette subfamily B member 5 | ENSBTAG00000002595 | -2,290 | 1,04E-02 |
| ACAP1 | ArfGAP with coiled-coil, ankyrin repeat and PH domains 1 | ENSBTAG00000010532 | -0,216 | 4,18E-02 |
| ACSM4 | acyl-CoA synthetase medium chain family member 4 | ENSBTAG00000046098 | -3,598 | 3,58E-02 |
| AK4 | adenylate kinase 4 | ENSBTAG00000020774 | 0,346 | 3,50E-03 |
| ANKRD35 | ankyrin repeat domain 35 | ENSBTAG00000038844 | 3,444 | 3,99E-02 |
| ATP6V1G2 | ATPase H+ transporting V1 subunit G2 | ENSBTAG00000014491 | 0,421 | 3,14E-02 |
| BAG4 | BAG cochaperone 4 | ENSBTAG00000013143 | -0,190 | 4,79E-02 |
| BCL2 | BCL2 apoptosis regulator | ENSBTAG00000019302 | 0,732 | 1,76E-02 |
| BET1 | Bet1 golgi vesicular membrane trafficking protein | ENSBTAG00000003424 | 0,233 | 3,69E-02 |
| C4orf33 | chromosome 4 open reading frame 33 | ENSBTAG00000044159 | 0,352 | 2,43E-02 |
| CCDC136 | coiled-coil domain containing 136 | ENSBTAG00000011002 | -0,346 | 1,34E-02 |
| CDKL3 | cyclin dependent kinase like 3 | ENSBTAG00000010979 | -0,416 | 3,91E-02 |
| CES2 | carboxylesterase 2 | ENSBTAG00000005093 | -0,230 | 3,12E-02 |
| COQ8B | coenzyme Q8B | ENSBTAG00000019462 | 0,207 | 3,44E-02 |
| CSMD2 | CUB and Sushi multiple domains 2 | ENSBTAG00000005784 | -3,308 | 3,20E-02 |
| CXCR4 | C-X-C motif chemokine receptor 4 | ENSBTAG00000001060 | 3,314 | 4,31E-02 |
| CYB561D1 | cytochrome b561 family member D1 | ENSBTAG00000016643 | 0,289 | 4,12E-02 |
| CYP27A1 | cytochrome P450 family 27 subfamily A member 1 | ENSBTAG00000013489 | 1,308 | 2,18E-02 |
| DAP | death associated protein | ENSBTAG00000006346 | 3,651 | 3,93E-02 |
| DGCR2 | DiGeorge syndrome critical region gene 2 | ENSBTAG00000000429 | 0,224 | 3,97E-02 |
| DISC1 | DISC1 scaffold protein | ENSBTAG00000050839 | 0,522 | 2,52E-02 |
| DLL4 | delta like canonical Notch ligand 4 | ENSBTAG00000010361 | -2,064 | 1,05E-02 |
| DMRT2 | doublesex and mab-3 related transcription factor 2 | ENSBTAG00000008062 | -2,644 | 4,87E-02 |
| DNTTIP2 | deoxynucleotidyltransferase terminal interacting protein 2 | ENSBTAG00000047679 | -0,153 | 4,13E-02 |
| DOC2A | double C2 domain alpha | ENSBTAG00000005031 | -0,693 | 4,45E-02 |
| DYNLRB2 | dynein light chain roadblock-type 2 | ENSBTAG00000017752 | 0,841 | 1,62E-02 |
| EIF5B | eukaryotic translation initiation factor 5B | ENSBTAG00000034255 | -0,107 | 4,54E-02 |
| ELMOD3 | ELMO domain containing 3 | ENSBTAG00000021935 | -0,576 | 9,26E-03 |
| FAM78B | family with sequence similarity 78 member B | ENSBTAG00000047468 | -0,869 | 4,74E-02 |
| GMPPA | GDP-mannose pyrophosphorylase A | ENSBTAG00000002995 | -0,186 | 1,94E-02 |
| GPR88 | G protein-coupled receptor 88 | ENSBTAG00000021101 | 3,801 | 4,01E-02 |
| GRM8 | glutamate metabotropic receptor 8 | ENSBTAG00000054974 | 2,540 | 1,97E-02 |
| HAS2 | hyaluronan synthase 2 | ENSBTAG00000019892 | -0,299 | 8,38E-03 |
| HDAC10 | histone deacetylase 10 | ENSBTAG00000011000 | 0,361 | 2,54E-02 |
| HIGD1C | HIG1 hypoxia inducible domain family member 1C | ENSBTAG00000047358 | -3,731 | 4,90E-02 |
| HS3ST3A1 | heparan sulfate-glucosamine 3-sulfotransferase 3A1 | ENSBTAG00000031107 | 0,956 | 1,32E-02 |
| IFT74 | intraflagellar transport 74 | ENSBTAG00000019568 | -0,175 | 4,09E-02 |
| IRF7 | interferon regulatory factor 7 | ENSBTAG00000047680 | 0,477 | 4,19E-02 |
| KLHL25 | kelch like family member 25 | ENSBTAG00000016091 | 0,386 | 4,49E-02 |
| KRTCAP3 | keratinocyte associated protein 3 | ENSBTAG00000018156 | 2,074 | 2,72E-03 |
| LEKR1 | leucine, glutamate and lysine rich 1 | ENSBTAG00000050129 | -1,851 | 2,93E-02 |
| LIPT1 | lipoyltransferase 1 | ENSBTAG00000003965 | -0,702 | 6,48E-03 |
| LNX2 | ligand of numb-protein X 2 | ENSBTAG00000015614 | 0,409 | 5,73E-03 |
| MASP1 | MBL associated serine protease 1 | ENSBTAG00000012467 | 0,788 | 3,88E-02 |
| MDH1B | malate dehydrogenase 1B | ENSBTAG00000016192 | 0,949 | 1,36E-02 |
| METTL1 | methyltransferase like 1 | ENSBTAG00000016908 | -0,227 | 4,65E-02 |
| MRPS18B | mitochondrial ribosomal protein S18B | ENSBTAG00000006936 | -0,163 | 4,71E-02 |
| MTM1 | myotubularin 1 | ENSBTAG00000014138 | 0,271 | 2,06E-02 |
| MYPOP | Myb related transcription factor, partner of profilin | ENSBTAG00000045708 | 0,363 | 6,20E-03 |
| NAA60 | N-alpha-acetyltransferase 60, NatF catalytic subunit | ENSBTAG00000004875 | -0,184 | 3,59E-02 |
| NADSYN1 | NAD synthetase 1 | ENSBTAG00000016470 | 0,353 | 1,07E-05 |
| NAIP | NLR family apoptosis inhibitory protein | ENSBTAG00000003326 | -0,390 | 4,48E-02 |
| NGFR | nerve growth factor receptor | ENSBTAG00000020979 | -3,687 | 4,05E-02 |
| NME5 | NME/NM23 family member 5 | ENSBTAG00000008752 | -0,248 | 4,74E-02 |
| OARD1 | O-acyl-ADP-ribose deacylase 1 | ENSBTAG00000005975 | -0,256 | 4,75E-02 |
| OVGP1 | oviductal glycoprotein 1 | ENSBTAG00000024849 | 3,442 | 3,72E-02 |
| P2RX5 | purinergic receptor P2X 5 | ENSBTAG00000015258 | -1,325 | 3,17E-02 |
| PCDHA13 | protocadherin alpha 13 | ENSBTAG00000053546 | 0,200 | 3,15E-02 |
| PDE5A | phosphodiesterase 5A | ENSBTAG00000024888 | 0,201 | 3,52E-02 |
| PLK5 | polo like kinase 5 (inactive) | ENSBTAG00000012150 | -1,515 | 3,34E-02 |
| PNKD | PNKD metallo-beta-lactamase domain containing | ENSBTAG00000051631 | -0,294 | 3,27E-02 |
| POLR2D | RNA polymerase II subunit D | ENSBTAG00000053244 | -0,321 | 2,42E-02 |
| PRIMPOL | primase and DNA directed polymerase | ENSBTAG00000018863 | 0,247 | 4,29E-02 |
| PRKN | parkin RBR E3 ubiquitin protein ligase | ENSBTAG00000018996 | 0,629 | 4,50E-02 |
| PSMA1 | proteasome 20S subunit alpha 1 | ENSBTAG00000006564 | -0,098 | 4,48E-02 |
| PSMC1 | proteasome 26S subunit, ATPase 1 | ENSBTAG00000005426 | -0,127 | 3,10E-02 |
| PTGFR | prostaglandin F receptor | ENSBTAG00000015902 | 0,261 | 4,60E-02 |
| RALGPS1 | Ral GEF with PH domain and SH3 binding motif 1 | ENSBTAG00000023843 | 0,400 | 3,02E-03 |
| RFFL | ring finger and FYVE like domain containing E3 ubiquitin protein ligase | ENSBTAG00000013645 | 0,271 | 4,94E-02 |
| RPL35 | ribosomal protein L35 | ENSBTAG00000003205 | -1,286 | 1,25E-02 |
| RSPH1 | radial spoke head component 1 | ENSBTAG00000021284 | 2,263 | 3,20E-02 |
| RSPO3 | R-spondin 3 | ENSBTAG00000008121 | -1,213 | 3,11E-02 |
| SCLY | selenocysteine lyase | ENSBTAG00000017826 | 0,561 | 1,64E-02 |
| SETD9 | SET domain containing 9 | ENSBTAG00000013426 | -0,441 | 1,01E-02 |
| SH3BGRL2 | SH3 domain binding glutamate rich protein like 2 | ENSBTAG00000055014 | -0,896 | 1,19E-02 |
| SLC26A1 | solute carrier family 26 member 1 | ENSBTAG00000007001 | -0,436 | 1,97E-02 |
| SLC35G6 | solute carrier family 35 member G6 | ENSBTAG00000016370 | -2,260 | 2,45E-02 |
| SLC38A9 | solute carrier family 38 member 9 | ENSBTAG00000033313 | -0,253 | 1,91E-02 |
| SLC39A14 | solute carrier family 39 member 14 | ENSBTAG00000019225 | 0,191 | 4,53E-02 |
| SLC4A8 | solute carrier family 4 member 8 | ENSBTAG00000017950 | -0,445 | 1,80E-02 |
| SMIM19 | small integral membrane protein 19 | ENSBTAG00000000979 | -4,350 | 2,11E-02 |
| SPAG1 | sperm associated antigen 1 | ENSBTAG00000032544 | -0,580 | 3,52E-02 |
| SPATA13 | spermatogenesis associated 13 | ENSBTAG00000019545 | -0,623 | 2,71E-02 |
| SPATA18 | spermatogenesis associated 18 | ENSBTAG00000018106 | -0,297 | 4,79E-02 |
| SPTLC3 | serine palmitoyltransferase long chain base subunit 3 | ENSBTAG00000014252 | 0,402 | 3,48E-02 |
| STS | steroid sulfatase | ENSBTAG00000020789 | -1,333 | 4,07E-02 |
| SYP | synaptophysin | ENSBTAG00000016096 | 0,859 | 3,02E-02 |
| TAZ | tafazzin | ENSBTAG00000055101 | -3,258 | 1,95E-02 |
| TEX13A | testis expressed 13A | ENSBTAG00000048086 | -1,931 | 1,66E-02 |
| TRIM52 | tripartite motif containing 52 | ENSBTAG00000019651 | 0,912 | 1,39E-02 |
| TRMT6 | tRNA methyltransferase 6 | ENSBTAG00000001314 | -0,158 | 2,36E-02 |
| TTC9 | tetratricopeptide repeat domain 9 | ENSBTAG00000045604 | -1,120 | 2,59E-02 |
| USP19 | ubiquitin specific peptidase 19 | ENSBTAG00000018921 | 0,080 | 4,96E-02 |
| USP28 | ubiquitin specific peptidase 28 | ENSBTAG00000002323 | -0,229 | 4,47E-02 |
| WDR74 | WD repeat domain 74 | ENSBTAG00000009491 | -0,216 | 1,80E-02 |
| XPNPEP3 | X-prolyl aminopeptidase 3 | ENSBTAG00000006204 | -0,376 | 2,89E-02 |
| ZBTB26 | zinc finger and BTB domain containing 26 | ENSBTAG00000038610 | 0,297 | 3,76E-02 |
| ZBTB49 | zinc finger and BTB domain containing 49 | ENSBTAG00000014680 | 0,568 | 3,58E-02 |
| ZDHHC14 | zinc finger DHHC-type palmitoyltransferase 14 | ENSBTAG00000018464 | 0,204 | 2,50E-02 |
| ZNF304 | zinc finger protein 304 | ENSBTAG00000017872 | 0,405 | 4,67E-02 |
| ZNF354A | zinc finger protein 354A | ENSBTAG00000006428 | -0,264 | 4,92E-02 |
| ZNF354B | zinc finger protein 354B | ENSBTAG00000017123 | 1,073 | 4,51E-03 |
| ZRANB1 | zinc finger RANBP2-type containing 1 | ENSBTAG00000003395 | 0,081 | 4,31E-02 |

**Supplemental Table S3: List of DEGs in melatonin + N-acetyl serotonin treatment (FC=0, adjusted p-value ≤ 0.05)**

| Symbol | Entrez Gene Name | ID | Expr Log Ratio | Expr p-value |
| --- | --- | --- | --- | --- |
| ABCB5 | ATP binding cassette subfamily B member 5 | ENSBTAG00000002595 | -1,647 | 4,79E-02 |
| ABCC9 | ATP binding cassette subfamily C member 9 | ENSBTAG00000019294 | -3,862 | 1,04E-02 |
| AFP | alpha fetoprotein | ENSBTAG00000017131 | -2,131 | 3,29E-02 |
| AKR1C3 | aldo-keto reductase family 1 member C3 | ENSBTAG00000022570 | -0,647 | 2,73E-02 |
| ANKRD35 | ankyrin repeat domain 35 | ENSBTAG00000038844 | 3,509 | 3,60E-02 |
| AP1G2 | adaptor related protein complex 1 subunit gamma 2 | ENSBTAG00000007099 | -0,222 | 4,49E-02 |
| AP3B1 | adaptor related protein complex 3 subunit beta 1 | ENSBTAG00000005016 | 0,100 | 3,47E-02 |
| AQP1 | aquaporin 1 (Colton blood group) | ENSBTAG00000000745 | -1,722 | 1,56E-02 |
| ATG16L2 | autophagy related 16 like 2 | ENSBTAG00000019059 | -0,385 | 1,57E-02 |
| CAMK2A | calcium/calmodulin dependent protein kinase II alpha | ENSBTAG00000020087 | -0,716 | 1,54E-02 |
| CAPN3 | calpain 3 | ENSBTAG00000008868 | -0,418 | 5,45E-03 |
| CCDC136 | coiled-coil domain containing 136 | ENSBTAG00000011002 | -0,336 | 1,64E-02 |
| CCDC3 | coiled-coil domain containing 3 | ENSBTAG00000040490 | 1,761 | 8,97E-03 |
| CDC25B | cell division cycle 25B | ENSBTAG00000008436 | -0,210 | 4,23E-02 |
| CDCA2 | cell division cycle associated 2 | ENSBTAG00000002756 | -0,695 | 1,86E-02 |
| Celf6 | CUGBP, Elav-like family member 6 | ENSBTAG00000014092 | -0,649 | 2,30E-03 |
| CEP85 | centrosomal protein 85 | ENSBTAG00000009579 | -0,254 | 1,08E-02 |
| CFAP69 | cilia and flagella associated protein 69 | ENSBTAG00000003508 | -0,769 | 2,62E-02 |
| CHEK2 | checkpoint kinase 2 | ENSBTAG00000004956 | -0,394 | 3,71E-02 |
| CLIC2 | chloride intracellular channel 2 | ENSBTAG00000010948 | -0,664 | 3,02E-02 |
| CMYA5 | cardiomyopathy associated 5 | ENSBTAG00000006823 | -1,420 | 1,40E-02 |
| COA7 | cytochrome c oxidase assembly factor 7 | ENSBTAG00000002050 | -0,216 | 2,44E-02 |
| CPT1B | carnitine palmitoyltransferase 1B | ENSBTAG00000016048 | -0,341 | 1,75E-02 |
| CROCC | ciliary rootlet coiled-coil, rootletin | ENSBTAG00000003822 | -0,263 | 4,06E-02 |
| CRYM | crystallin mu | ENSBTAG00000009842 | -1,810 | 3,44E-02 |
| CTDP1 | CTD phosphatase subunit 1 | ENSBTAG00000053484 | 0,253 | 3,45E-02 |
| DDB2 | damage specific DNA binding protein 2 | ENSBTAG00000020999 | -0,228 | 4,51E-02 |
| DHH | desert hedgehog signaling molecule | ENSBTAG00000000124 | 3,536 | 3,27E-02 |
| DLL4 | delta like canonical Notch ligand 4 | ENSBTAG00000010361 | -3,424 | 1,03E-03 |
| DMAC2L | distal membrane arm assembly component 2 like | ENSBTAG00000015202 | 0,470 | 4,42E-02 |
| DMC1 | DNA meiotic recombinase 1 | ENSBTAG00000005936 | -3,772 | 7,28E-03 |
| DNAJA3 | DnaJ heat shock protein family (Hsp40) member A3 | ENSBTAG00000010013 | -0,258 | 3,84E-02 |
| DNTTIP2 | deoxynucleotidyltransferase terminal interacting protein 2 | ENSBTAG00000047679 | -0,149 | 4,66E-02 |
| DUSP12 | dual specificity phosphatase 12 | ENSBTAG00000021754 | 0,181 | 4,07E-02 |
| DYNLRB2 | dynein light chain roadblock-type 2 | ENSBTAG00000017752 | 0,864 | 1,35E-02 |
| DZIP3 | DAZ interacting zinc finger protein 3 | ENSBTAG00000006158 | -0,162 | 4,64E-02 |
| EEF2K | eukaryotic elongation factor 2 kinase | ENSBTAG00000017662 | -0,170 | 2,41E-02 |
| EFEMP1 | EGF containing fibulin extracellular matrix protein 1 | ENSBTAG00000017448 | 0,147 | 4,51E-02 |
| EXTL2 | exostosin like glycosyltransferase 2 | ENSBTAG00000019026 | 0,174 | 1,98E-02 |
| FBXO43 | F-box protein 43 | ENSBTAG00000019795 | -0,901 | 1,74E-02 |
| FRMPD2 | FERM and PDZ domain containing 2 | ENSBTAG00000005568 | -2,343 | 1,57E-02 |
| GABARAPL1 | GABA type A receptor associated protein like 1 | ENSBTAG00000011765 | 0,115 | 4,63E-02 |
| GEMIN6 | gem nuclear organelle associated protein 6 | ENSBTAG00000015521 | -0,273 | 3,06E-02 |
| GGPS1 | geranylgeranyl diphosphate synthase 1 | ENSBTAG00000013068 | 0,124 | 4,95E-02 |
| Gm16500 |  | ENSBTAG00000022109 | -0,180 | 4,85E-02 |
| GMFG | glia maturation factor gamma | ENSBTAG00000005390 | -0,901 | 4,68E-02 |
| GNAT2 | G protein subunit alpha transducin 2 | ENSBTAG00000013017 | -0,721 | 1,63E-02 |
| GPAT3 | glycerol-3-phosphate acyltransferase 3 | ENSBTAG00000017592 | -0,723 | 2,73E-02 |
| GRM8 | glutamate metabotropic receptor 8 | ENSBTAG00000054974 | 2,527 | 2,05E-02 |
| HNRNPH1 | heterogeneous nuclear ribonucleoprotein H1 | ENSBTAG00000009389 | -0,145 | 4,30E-02 |
| HOXA11 | homeobox A11 | ENSBTAG00000014738 | 0,641 | 4,43E-02 |
| HTR4 | 5-hydroxytryptamine receptor 4 | ENSBTAG00000010837 | -1,212 | 2,39E-02 |
| IGHMBP2 | immunoglobulin mu DNA binding protein 2 | ENSBTAG00000022185 | -0,318 | 3,00E-02 |
| IL6R | interleukin 6 receptor | ENSBTAG00000018474 | -1,746 | 2,72E-02 |
| ISL2 | ISL LIM homeobox 2 | ENSBTAG00000016651 | 0,708 | 2,06E-03 |
| ITGAL | integrin subunit alpha L | ENSBTAG00000007103 | -1,942 | 2,05E-02 |
| KCNJ10 | potassium inwardly rectifying channel subfamily J member 10 | ENSBTAG00000002414 | 0,469 | 3,80E-02 |
| KDSR | 3-ketodihydrosphingosine reductase | ENSBTAG00000007723 | 0,256 | 4,46E-02 |
| LENG8 | leukocyte receptor cluster member 8 | ENSBTAG00000011689 | -0,280 | 1,92E-02 |
| LLGL2 | LLGL scribble cell polarity complex component 2 | ENSBTAG00000020067 | -0,445 | 4,49E-02 |
| LMF1 | lipase maturation factor 1 | ENSBTAG00000019745 | 0,427 | 3,75E-02 |
| Lmo3 | LIM domain only 3 | ENSBTAG00000013541 | -1,051 | 6,03E-03 |
| LNX2 | ligand of numb-protein X 2 | ENSBTAG00000015614 | 0,320 | 3,22E-02 |
| MAPK8IP3 | mitogen-activated protein kinase 8 interacting protein 3 | ENSBTAG00000002211 | -0,229 | 3,23E-02 |
| MATN4 | matrilin 4 | ENSBTAG00000011279 | -3,868 | 1,91E-02 |
| MDM4 | MDM4 regulator of p53 | ENSBTAG00000006255 | -0,384 | 2,87E-03 |
| MMRN2 | multimerin 2 | ENSBTAG00000003510 | -1,104 | 3,08E-02 |
| Msantd2 | Myb/SANT-like DNA-binding domain containing 2 | ENSBTAG00000011248 | -0,214 | 4,86E-02 |
| MSH5 | mutS homolog 5 | ENSBTAG00000019790 | -0,336 | 3,36E-02 |
| MTARC1 | mitochondrial amidoxime reducing component 1 | ENSBTAG00000047287 | -0,521 | 3,86E-02 |
| MYPOP | Myb related transcription factor, partner of profilin | ENSBTAG00000045708 | 0,281 | 3,53E-02 |
| NADSYN1 | NAD synthetase 1 | ENSBTAG00000016470 | 0,160 | 4,75E-02 |
| OMG | oligodendrocyte myelin glycoprotein | ENSBTAG00000025213 | -0,983 | 3,98E-02 |
| OSBPL7 | oxysterol binding protein like 7 | ENSBTAG00000013523 | -0,248 | 9,56E-03 |
| PABPC1L | poly(A) binding protein cytoplasmic 1 like | ENSBTAG00000016849 | -0,434 | 3,77E-02 |
| PCSK4 | proprotein convertase subtilisin/kexin type 4 | ENSBTAG00000002305 | -0,566 | 5,57E-03 |
| PCTP | phosphatidylcholine transfer protein | ENSBTAG00000018706 | 0,277 | 4,64E-02 |
| PDE5A | phosphodiesterase 5A | ENSBTAG00000024888 | 0,188 | 4,99E-02 |
| PNN | pinin, desmosome associated protein | ENSBTAG00000026995 | -0,135 | 4,75E-02 |
| POPDC2 | popeye domain containing 2 | ENSBTAG00000005604 | 3,091 | 4,39E-02 |
| PRRG4 | proline rich and Gla domain 4 | ENSBTAG00000020731 | 0,581 | 3,60E-02 |
| PTGFR | prostaglandin F receptor | ENSBTAG00000015902 | 0,275 | 3,55E-02 |
| PTPRO | protein tyrosine phosphatase receptor type O | ENSBTAG00000006256 | 2,452 | 1,80E-02 |
| RAPSN | receptor associated protein of the synapse | ENSBTAG00000021745 | -1,360 | 1,67E-02 |
| RASD1 | ras related dexamethasone induced 1 | ENSBTAG00000020520 | 0,917 | 4,12E-02 |
| RBM6 | RNA binding motif protein 6 | ENSBTAG00000006328 | -0,170 | 2,06E-02 |
| RDH11 | retinol dehydrogenase 11 | ENSBTAG00000001950 | 0,207 | 4,34E-02 |
| RECQL5 | RecQ like helicase 5 | ENSBTAG00000011715 | -0,248 | 3,64E-03 |
| RFK | riboflavin kinase | ENSBTAG00000019345 | 0,191 | 3,32E-02 |
| RPL17 | ribosomal protein L17 | ENSBTAG00000022902 | 2,570 | 8,07E-03 |
| SCAMP2 | secretory carrier membrane protein 2 | ENSBTAG00000005844 | 0,104 | 4,45E-02 |
| SCRN2 | secernin 2 | ENSBTAG00000001828 | 0,318 | 1,59E-02 |
| SEC61A2 | SEC61 translocon subunit alpha 2 | ENSBTAG00000019508 | -0,251 | 2,54E-02 |
| SEMA4A | semaphorin 4A | ENSBTAG00000012228 | -0,738 | 3,98E-02 |
| SEPTIN1 | septin 1 | ENSBTAG00000021219 | -1,089 | 3,70E-02 |
| SERPINE1 | serpin family E member 1 | ENSBTAG00000014465 | 0,143 | 4,13E-02 |
| SH2D2A | SH2 domain containing 2A | ENSBTAG00000024470 | -2,177 | 4,76E-02 |
| SLAMF6 | SLAM family member 6 | ENSBTAG00000014368 | -1,028 | 3,61E-02 |
| SLC23A3 | solute carrier family 23 member 3 | ENSBTAG00000012618 | -0,973 | 1,85E-02 |
| SLC26A1 | solute carrier family 26 member 1 | ENSBTAG00000007001 | -0,593 | 1,78E-03 |
| SLC52A2 | solute carrier family 52 member 2 | ENSBTAG00000000857 | 0,262 | 4,91E-02 |
| SMC1B | structural maintenance of chromosomes 1B | ENSBTAG00000024132 | -1,977 | 2,02E-02 |
| SNAPC3 | small nuclear RNA activating complex polypeptide 3 | ENSBTAG00000004592 | 0,156 | 4,55E-02 |
| SPARC | secreted protein acidic and cysteine rich | ENSBTAG00000014835 | 0,103 | 4,05E-02 |
| SPARCL1 | SPARC like 1 | ENSBTAG00000004094 | -3,446 | 3,41E-02 |
| TBC1D22B | TBC1 domain family member 22B | ENSBTAG00000014253 | 0,229 | 3,53E-03 |
| TBX4 | T-box transcription factor 4 | ENSBTAG00000009968 | -3,286 | 4,23E-02 |
| TESC | tescalcin | ENSBTAG00000044078 | -0,285 | 4,33E-02 |
| TFPI | tissue factor pathway inhibitor | ENSBTAG00000049919 | -0,367 | 3,93E-02 |
| TNRC6A | trinucleotide repeat containing adaptor 6A | ENSBTAG00000017999 | -0,188 | 4,22E-02 |
| TRMT44 | tRNA methyltransferase 44 homolog | ENSBTAG00000004797 | -0,454 | 4,14E-02 |
| TTC23 | tetratricopeptide repeat domain 23 | ENSBTAG00000011185 | -0,368 | 3,83E-02 |
| UBE2O | ubiquitin conjugating enzyme E2 O | ENSBTAG00000020115 | -0,139 | 3,13E-02 |
| VWA7 | von Willebrand factor A domain containing 7 | ENSBTAG00000005630 | -0,958 | 4,88E-02 |
| ZFC3H1 | zinc finger C3H1-type containing | ENSBTAG00000012267 | -0,131 | 3,34E-02 |
| ZNF213 | zinc finger protein 213 | ENSBTAG00000026408 | -0,424 | 1,44E-02 |
| ZNF326 | zinc finger protein 326 | ENSBTAG00000019133 | -0,127 | 3,93E-02 |

**Supplemental Table S4: List of DEGs in melatonin + S26131 treatment (FC=0, adjusted p-value ≤ 0.05)**

| Symbol | Entrez Gene Name | ID | Expr Log Ratio | Expr p-value |
| --- | --- | --- | --- | --- |
| 1300017J02Rik | RIKEN cDNA 1300017J02 gene | ENSBTAG00000023411 | -3,487 | 3,43E-02 |
| AFP | alpha fetoprotein | ENSBTAG00000017131 | -2,599 | 1,66E-02 |
| AGO1 | argonaute RISC component 1 | ENSBTAG00000012253 | -0,290 | 2,37E-02 |
| AMOT | angiomotin | ENSBTAG00000016221 | -0,275 | 2,18E-02 |
| APAF1 | apoptotic peptidase activating factor 1 | ENSBTAG00000021661 | -0,209 | 1,34E-02 |
| AQP1 | aquaporin 1 (Colton blood group) | ENSBTAG00000000745 | -1,591 | 2,55E-02 |
| ARPP19 | cAMP regulated phosphoprotein 19 | ENSBTAG00000011022 | -0,176 | 7,90E-03 |
| ATF7IP | activating transcription factor 7 interacting protein | ENSBTAG00000003221 | -0,186 | 2,96E-02 |
| ATL3 | atlastin GTPase 3 | ENSBTAG00000003330 | -0,255 | 1,80E-02 |
| B3GAT3 | beta-1,3-glucuronyltransferase 3 | ENSBTAG00000016950 | 0,174 | 3,50E-02 |
| BBLN | bublin coiled coil protein | ENSBTAG00000014150 | 0,170 | 4,22E-02 |
| BMX | BMX non-receptor tyrosine kinase | ENSBTAG00000000377 | 1,356 | 3,15E-02 |
| BNC2 | basonuclin 2 | ENSBTAG00000003669 | -0,303 | 2,27E-02 |
| BRAT1 | BRCA1 associated ATM activator 1 | ENSBTAG00000014502 | 0,257 | 4,31E-02 |
| C11orf87 | chromosome 11 open reading frame 87 | ENSBTAG00000050066 | -1,281 | 1,37E-02 |
| C7 | complement C7 | ENSBTAG00000011766 | -0,226 | 2,54E-02 |
| CBL | Cbl proto-oncogene | ENSBTAG00000006817 | -0,329 | 3,79E-02 |
| CBX2 | chromobox 2 | ENSBTAG00000038306 | 0,502 | 4,61E-02 |
| CBX5 | chromobox 5 | ENSBTAG00000006246 | -0,403 | 1,20E-03 |
| CCDC136 | coiled-coil domain containing 136 | ENSBTAG00000011002 | -0,429 | 2,41E-03 |
| CCDC146 | coiled-coil domain containing 146 | ENSBTAG00000009341 | -0,462 | 3,92E-02 |
| CDK19 | cyclin dependent kinase 19 | ENSBTAG00000007288 | -0,227 | 4,55E-02 |
| CDKL1 | cyclin dependent kinase like 1 | ENSBTAG00000004780 | -0,543 | 2,56E-02 |
| CDKL4 | cyclin dependent kinase like 4 | ENSBTAG00000024044 | 3,006 | 2,05E-02 |
| CEP295 | centrosomal protein 295 | ENSBTAG00000001902 | 0,312 | 1,76E-02 |
| CEP97 | centrosomal protein 97 | ENSBTAG00000013464 | -0,309 | 1,73E-02 |
| CFAP126 | cilia and flagella associated protein 126 | ENSBTAG00000015856 | -0,305 | 4,66E-02 |
| CFLAR | CASP8 and FADD like apoptosis regulator | ENSBTAG00000010998 | -0,371 | 1,89E-02 |
| CHST9 | carbohydrate sulfotransferase 9 | ENSBTAG00000014082 | -3,501 | 2,95E-02 |
| CLASP2 | cytoplasmic linker associated protein 2 | ENSBTAG00000011333 | -0,151 | 2,79E-02 |
| CLCF1 | cardiotrophin like cytokine factor 1 | ENSBTAG00000046110 | 0,212 | 2,57E-02 |
| CLOCK | clock circadian regulator | ENSBTAG00000044044 | -0,303 | 1,85E-02 |
| COL14A1 | collagen type XIV alpha 1 chain | ENSBTAG00000013369 | -0,376 | 1,35E-02 |
| COQ8B | coenzyme Q8B | ENSBTAG00000019462 | 0,197 | 4,49E-02 |
| CPA3 | carboxypeptidase A3 | ENSBTAG00000015008 | -1,149 | 3,95E-02 |
| CSNK2B | casein kinase 2 beta | ENSBTAG00000008837 | 0,167 | 2,24E-02 |
| CUL4A | cullin 4A | ENSBTAG00000020093 | -0,158 | 3,95E-02 |
| DDX6 | DEAD-box helicase 6 | ENSBTAG00000009067 | -0,293 | 8,47E-03 |
| DEDD2 | death effector domain containing 2 | ENSBTAG00000005761 | 0,178 | 2,17E-02 |
| DNM1L | dynamin 1 like | ENSBTAG00000011395 | -0,106 | 3,84E-02 |
| DOK1 | docking protein 1 | ENSBTAG00000018579 | 0,270 | 2,84E-02 |
| DSTYK | dual serine/threonine and tyrosine protein kinase | ENSBTAG00000016618 | -0,144 | 4,95E-02 |
| DYNLRB2 | dynein light chain roadblock-type 2 | ENSBTAG00000017752 | 0,733 | 3,84E-02 |
| DYRK1B | dual specificity tyrosine phosphorylation regulated kinase 1B | ENSBTAG00000012509 | 0,109 | 3,65E-02 |
| EGFLAM | EGF like, fibronectin type III and laminin G domains | ENSBTAG00000019595 | -2,757 | 3,88E-02 |
| EIF2D | eukaryotic translation initiation factor 2D | ENSBTAG00000010432 | 0,162 | 1,87E-02 |
| EPHA5 | EPH receptor A5 | ENSBTAG00000009438 | -0,338 | 4,17E-02 |
| EPHX4 | epoxide hydrolase 4 | ENSBTAG00000005805 | 1,200 | 1,24E-03 |
| ERCC4 | ERCC excision repair 4, endonuclease catalytic subunit | ENSBTAG00000021773 | -0,256 | 1,27E-02 |
| ETHE1 | ETHE1 persulfide dioxygenase | ENSBTAG00000004379 | 0,198 | 3,87E-02 |
| FAM149B1 | family with sequence similarity 149 member B1 | ENSBTAG00000019130 | 0,240 | 2,34E-02 |
| FAM20B | FAM20B glycosaminoglycan xylosylkinase | ENSBTAG00000013999 | -0,247 | 4,56E-02 |
| FBXO15 | F-box protein 15 | ENSBTAG00000009686 | 4,142 | 1,90E-02 |
| FLAD1 | flavin adenine dinucleotide synthetase 1 | ENSBTAG00000000186 | 0,203 | 4,50E-02 |
| GCHFR | GTP cyclohydrolase I feedback regulator | ENSBTAG00000013477 | 0,899 | 1,83E-02 |
| GGPS1 | geranylgeranyl diphosphate synthase 1 | ENSBTAG00000013068 | 0,129 | 4,13E-02 |
| GJC1 | gap junction protein gamma 1 | ENSBTAG00000004036 | -0,248 | 1,36E-02 |
| GLRA2 | glycine receptor alpha 2 | ENSBTAG00000017209 | -1,560 | 3,10E-02 |
| GPR63 | G protein-coupled receptor 63 | ENSBTAG00000002915 | 0,977 | 3,11E-02 |
| GSPT2 | G1 to S phase transition 2 | ENSBTAG00000013311 | -0,189 | 2,85E-02 |
| GSTA1 | glutathione S-transferase alpha 1 | ENSBTAG00000006546 | -4,235 | 4,93E-02 |
| GTF2E1 | general transcription factor IIE subunit 1 | ENSBTAG00000016848 | -0,272 | 2,82E-02 |
| GUCY1A2 | guanylate cyclase 1 soluble subunit alpha 2 | ENSBTAG00000044144 | -0,698 | 1,03E-02 |
| H2AC6 | H2A clustered histone 6 | ENSBTAG00000039657 | -4,112 | 4,60E-03 |
| H3C13 | H3 clustered histone 13 | ENSBTAG00000024003 | -1,121 | 4,34E-02 |
| HAS2 | hyaluronan synthase 2 | ENSBTAG00000019892 | -0,261 | 2,26E-02 |
| HECA | hdc homolog, cell cycle regulator | ENSBTAG00000024595 | -0,291 | 4,87E-02 |
| HINFP | histone H4 transcription factor | ENSBTAG00000015015 | 0,165 | 3,55E-02 |
| HOOK3 | hook microtubule tethering protein 3 | ENSBTAG00000007634 | -0,282 | 4,60E-02 |
| ICA1L | islet cell autoantigen 1 like | ENSBTAG00000008673 | -0,394 | 6,06E-03 |
| IER2 | immediate early response 2 | ENSBTAG00000016354 | 0,286 | 3,83E-02 |
| ING5 | inhibitor of growth family member 5 | ENSBTAG00000009123 | 0,276 | 2,83E-02 |
| INHBA | inhibin subunit beta A | ENSBTAG00000048508 | -0,173 | 2,25E-02 |
| INO80D | INO80 complex subunit D | ENSBTAG00000014407 | -0,399 | 4,05E-02 |
| ISL2 | ISL LIM homeobox 2 | ENSBTAG00000016651 | 0,557 | 1,72E-02 |
| ITGB3 | integrin subunit beta 3 | ENSBTAG00000009987 | -0,167 | 2,02E-02 |
| KCNT1 | potassium sodium-activated channel subfamily T member 1 | ENSBTAG00000018975 | -2,255 | 3,26E-02 |
| KLHL42 | kelch like family member 42 | ENSBTAG00000006372 | -0,214 | 2,14E-02 |
| KRTCAP3 | keratinocyte associated protein 3 | ENSBTAG00000018156 | 1,870 | 7,79E-03 |
| LOC100912052 | mRNA-decapping enzyme 2-like | ENSBTAG00000000767 | -0,281 | 2,84E-03 |
| LPGAT1 | lysophosphatidylglycerol acyltransferase 1 | ENSBTAG00000005137 | -0,292 | 6,79E-03 |
| LYPD6 | LY6/PLAUR domain containing 6 | ENSBTAG00000047426 | -0,271 | 4,67E-02 |
| MAPT | microtubule associated protein tau | ENSBTAG00000017512 | 0,502 | 2,81E-02 |
| MCM9 | minichromosome maintenance 9 homologous recombination repair factor | ENSBTAG00000002059 | -0,265 | 2,89E-02 |
| MDH1B | malate dehydrogenase 1B | ENSBTAG00000016192 | 1,040 | 7,01E-03 |
| MDM4 | MDM4 regulator of p53 | ENSBTAG00000006255 | -0,387 | 2,76E-03 |
| MEOX2 | mesenchyme homeobox 2 | ENSBTAG00000003238 | -2,296 | 3,37E-02 |
| MINDY2 | MINDY lysine 48 deubiquitinase 2 | ENSBTAG00000012391 | -0,261 | 4,93E-02 |
| NAA50 | N-alpha-acetyltransferase 50, NatE catalytic subunit | ENSBTAG00000007784 | -0,141 | 2,50E-02 |
| NADSYN1 | NAD synthetase 1 | ENSBTAG00000016470 | 0,323 | 6,70E-05 |
| NEGR1 | neuronal growth regulator 1 | ENSBTAG00000055278 | -0,267 | 2,79E-02 |
| NKAP | NFKB activating protein | ENSBTAG00000010884 | 0,181 | 4,96E-02 |
| NPTXR | neuronal pentraxin receptor | ENSBTAG00000000109 | -0,315 | 2,83E-02 |
| NUBP2 | nucleotide binding protein 2 | ENSBTAG00000016561 | 0,235 | 4,20E-02 |
| OPRL1 | opioid related nociceptin receptor 1 | ENSBTAG00000049473 | 0,732 | 1,73E-02 |
| OTUD3 | OTU deubiquitinase 3 | ENSBTAG00000017108 | -0,404 | 1,24E-02 |
| P2RX1 | purinergic receptor P2X 1 | ENSBTAG00000007169 | -0,725 | 4,96E-02 |
| PALS2 | protein associated with LIN7 2, MAGUK family member | ENSBTAG00000015303 | -0,187 | 1,01E-02 |
| PCGF1 | polycomb group ring finger 1 | ENSBTAG00000016606 | 0,196 | 2,97E-02 |
| PEX11B | peroxisomal biogenesis factor 11 beta | ENSBTAG00000008372 | 0,130 | 4,68E-02 |
| PEX12 | peroxisomal biogenesis factor 12 | ENSBTAG00000019723 | 0,259 | 1,65E-02 |
| PIK3R3 | phosphoinositide-3-kinase regulatory subunit 3 | ENSBTAG00000002979 | -0,209 | 3,83E-02 |
| POC5 | POC5 centriolar protein | ENSBTAG00000007114 | 0,277 | 2,70E-02 |
| POLB | DNA polymerase beta | ENSBTAG00000000225 | 0,235 | 1,01E-02 |
| POLR2D | RNA polymerase II subunit D | ENSBTAG00000053244 | -0,297 | 3,92E-02 |
| PPFIBP1 | PPFIA binding protein 1 | ENSBTAG00000004283 | -0,178 | 2,18E-02 |
| PRRX2 | paired related homeobox 2 | ENSBTAG00000002936 | -3,978 | 1,00E-02 |
| PTGFRN | prostaglandin F2 receptor inhibitor | ENSBTAG00000008022 | -0,125 | 3,11E-02 |
| PTPN14 | protein tyrosine phosphatase non-receptor type 14 | ENSBTAG00000021553 | -0,394 | 1,68E-02 |
| PWWP3A | PWWP domain containing 3A, DNA repair factor | ENSBTAG00000030839 | 0,254 | 4,43E-02 |
| RALGPS1 | Ral GEF with PH domain and SH3 binding motif 1 | ENSBTAG00000023843 | 0,312 | 2,27E-02 |
| RBM23 | RNA binding motif protein 23 | ENSBTAG00000038333 | 0,232 | 4,61E-02 |
| RGS14 | regulator of G protein signaling 14 | ENSBTAG00000008497 | 0,239 | 4,51E-02 |
| RMND5A | required for meiotic nuclear division 5 homolog A | ENSBTAG00000021140 | -0,167 | 2,04E-02 |
| RNF166 | ring finger protein 166 | ENSBTAG00000020942 | 0,243 | 1,61E-02 |
| RO60 | Ro60, Y RNA binding protein | ENSBTAG00000013812 | -0,191 | 3,33E-02 |
| ROCK2 | Rho associated coiled-coil containing protein kinase 2 | ENSBTAG00000005847 | -0,195 | 4,04E-02 |
| RPL14 | ribosomal protein L14 | ENSBTAG00000002038 | 0,137 | 4,37E-02 |
| RPL17 | ribosomal protein L17 | ENSBTAG00000022902 | 2,077 | 3,60E-02 |
| RPP14 | ribonuclease P/MRP subunit p14 | ENSBTAG00000019987 | 0,258 | 4,96E-02 |
| RPS25 | ribosomal protein S25 | ENSBTAG00000027772 | 2,771 | 4,79E-02 |
| RSBN1 | round spermatid basic protein 1 | ENSBTAG00000031940 | -0,163 | 2,62E-02 |
| RTN4RL1 | reticulon 4 receptor like 1 | ENSBTAG00000012302 | -2,469 | 2,95E-02 |
| SASH1 | SAM and SH3 domain containing 1 | ENSBTAG00000001826 | -0,333 | 1,24E-02 |
| SCRN2 | secernin 2 | ENSBTAG00000001828 | 0,276 | 3,80E-02 |
| SENP5 | SUMO specific peptidase 5 | ENSBTAG00000032674 | -0,178 | 2,21E-02 |
| SERINC5 | serine incorporator 5 | ENSBTAG00000001619 | -0,241 | 4,24E-02 |
| SETBP1 | SET binding protein 1 | ENSBTAG00000018088 | -1,057 | 1,33E-02 |
| SFT2D2 | SFT2 domain containing 2 | ENSBTAG00000037819 | -0,487 | 1,48E-02 |
| SKA2 | spindle and kinetochore associated complex subunit 2 | ENSBTAG00000021680 | -0,560 | 4,68E-02 |
| SLC16A4 | solute carrier family 16 member 4 | ENSBTAG00000015380 | 0,185 | 3,03E-02 |
| SLC6A6 | solute carrier family 6 member 6 | ENSBTAG00000011088 | -0,278 | 4,21E-02 |
| SMPD1 | sphingomyelin phosphodiesterase 1 | ENSBTAG00000015628 | 0,142 | 7,39E-03 |
| SNTB1 | syntrophin beta 1 | ENSBTAG00000044126 | -0,213 | 3,88E-02 |
| SOX7 | SRY-box transcription factor 7 | ENSBTAG00000054688 | -1,358 | 9,14E-04 |
| SP7 | Sp7 transcription factor | ENSBTAG00000033292 | 4,691 | 1,69E-02 |
| TANGO6 | transport and golgi organization 6 homolog | ENSBTAG00000013941 | -0,279 | 4,05E-02 |
| TERF1 | telomeric repeat binding factor 1 | ENSBTAG00000032982 | 0,188 | 3,57E-02 |
| TGFBR1 | transforming growth factor beta receptor 1 | ENSBTAG00000018035 | -0,221 | 1,03E-02 |
| THAP8 | THAP domain containing 8 | ENSBTAG00000014995 | 0,378 | 4,04E-02 |
| TKFC | triokinase and FMN cyclase | ENSBTAG00000018201 | 0,205 | 1,84E-02 |
| TMEM120B | transmembrane protein 120B | ENSBTAG00000009602 | -0,309 | 2,57E-02 |
| TMEM222 | transmembrane protein 222 | ENSBTAG00000010249 | 0,165 | 2,92E-02 |
| TMEM241 | transmembrane protein 241 | ENSBTAG00000010037 | -0,569 | 3,42E-03 |
| TNRC6A | trinucleotide repeat containing adaptor 6A | ENSBTAG00000017999 | -0,247 | 7,66E-03 |
| TRAPPC6A | trafficking protein particle complex subunit 6A | ENSBTAG00000007065 | 0,282 | 2,12E-02 |
| TTC9 | tetratricopeptide repeat domain 9 | ENSBTAG00000045604 | -1,159 | 2,11E-02 |
| TXNDC11 | thioredoxin domain containing 11 | ENSBTAG00000014823 | -0,116 | 4,63E-02 |
| USP19 | ubiquitin specific peptidase 19 | ENSBTAG00000018921 | 0,081 | 4,83E-02 |
| USP28 | ubiquitin specific peptidase 28 | ENSBTAG00000002323 | -0,299 | 9,09E-03 |
| USP31 | ubiquitin specific peptidase 31 | ENSBTAG00000011744 | -0,262 | 2,87E-02 |
| WIPI2 | WD repeat domain, phosphoinositide interacting 2 | ENSBTAG00000012049 | 0,153 | 7,54E-03 |
| XIAP | X-linked inhibitor of apoptosis | ENSBTAG00000040350 | -0,240 | 4,23E-02 |
| XPNPEP3 | X-prolyl aminopeptidase 3 | ENSBTAG00000006204 | -0,570 | 1,35E-03 |
| YTHDC2 | YTH domain containing 2 | ENSBTAG00000014099 | -0,122 | 4,49E-02 |
| ZBTB22 | zinc finger and BTB domain containing 22 | ENSBTAG00000016829 | 0,188 | 1,12E-02 |
| ZBTB34 | zinc finger and BTB domain containing 34 | ENSBTAG00000039343 | -0,412 | 8,30E-03 |
| ZDHHC4 | zinc finger DHHC-type palmitoyltransferase 4 | ENSBTAG00000009547 | 0,283 | 4,42E-02 |
| ZFX | zinc finger protein X-linked | ENSBTAG00000007730 | -0,192 | 4,32E-02 |
| ZNF445 | zinc finger protein 445 | ENSBTAG00000046301 | -0,287 | 1,53E-02 |
| ZNF652 | zinc finger protein 652 | ENSBTAG00000001919 | -0,343 | 2,71E-02 |
| ZNF704 | zinc finger protein 704 | ENSBTAG00000021743 | -0,824 | 3,56E-02 |
| ZNF711 | zinc finger protein 711 | ENSBTAG00000002668 | -2,642 | 3,55E-02 |
| ZYG11A | zyg-11 family member A, cell cycle regulator | ENSBTAG00000026032 | 1,008 | 2,22E-02 |

**Supplemental Table S5: List of DEGs in melatonin + IIk7 treatment (FC=0, adjusted p-value ≤ 0.05)**

| Symbol | ID | ID | Expr Log Ratio | Expr p-value |
| --- | --- | --- | --- | --- |
| RAB3IL1 | ENSBTAG00000015745 | ENSBTAG00000015745 | 0,501 | 7,47E-04 |
| PLCL1 | ENSBTAG00000007635 | ENSBTAG00000007635 | 0,525 | 1,19E-03 |
| ABCA3 | ENSBTAG00000014903 | ENSBTAG00000014903 | 0,296 | 1,42E-03 |
| CBX5 | ENSBTAG00000006246 | ENSBTAG00000006246 | -0,39 | 1,70E-03 |
| HSPA12A | ENSBTAG00000013779 | ENSBTAG00000013779 | 0,376 | 1,76E-03 |
| TRIM13 | ENSBTAG00000008173 | ENSBTAG00000008173 | 0,342 | 1,82E-03 |
| PTGER3 | ENSBTAG00000019230 | ENSBTAG00000019230 | 0,783 | 2,01E-03 |
| PNKD | ENSBTAG00000051631 | ENSBTAG00000051631 | -0,427 | 2,09E-03 |
| UMPS | ENSBTAG00000013727 | ENSBTAG00000013727 | -0,313 | 2,45E-03 |
| JAKMIP2 | ENSBTAG00000003349 | ENSBTAG00000003349 | -0,65 | 2,46E-03 |
| C1QTNF5 | ENSBTAG00000027766 | ENSBTAG00000027766 | 0,605 | 2,53E-03 |
| RETSAT | ENSBTAG00000021934 | ENSBTAG00000021934 | 0,196 | 2,58E-03 |
| EFCC1 | ENSBTAG00000005922 | ENSBTAG00000005922 | -2,256 | 2,66E-03 |
| GMFG | ENSBTAG00000005390 | ENSBTAG00000005390 | -1,471 | 2,77E-03 |
| TTC9 | ENSBTAG00000045604 | ENSBTAG00000045604 | -1,492 | 3,01E-03 |
| WDR74 | ENSBTAG00000009491 | ENSBTAG00000009491 | -0,271 | 3,06E-03 |
| VPS36 | ENSBTAG00000004307 | ENSBTAG00000004307 | 0,214 | 3,10E-03 |
| LGR5 | ENSBTAG00000013256 | ENSBTAG00000013256 | -0,614 | 3,31E-03 |
| AAMDC | ENSBTAG00000017298 | ENSBTAG00000017298 | -0,302 | 4,05E-03 |
| TMPO | ENSBTAG00000004240 | ENSBTAG00000004240 | -0,484 | 4,19E-03 |
| GPRIN1 | ENSBTAG00000044035 | ENSBTAG00000044035 | -0,614 | 4,26E-03 |
| EIF4ENIF1 | ENSBTAG00000008991 | ENSBTAG00000008991 | 0,222 | 4,48E-03 |
| PTK2B | ENSBTAG00000005958 | ENSBTAG00000005958 | 0,348 | 4,50E-03 |
| BUB1 | ENSBTAG00000021181 | ENSBTAG00000021181 | -0,342 | 4,68E-03 |
| TRIM66 | ENSBTAG00000020890 | ENSBTAG00000020890 | 0,422 | 4,85E-03 |
| ISM1 | ENSBTAG00000017188 | ENSBTAG00000017188 | -0,76 | 4,91E-03 |
| MAPT | ENSBTAG00000017512 | ENSBTAG00000017512 | 0,632 | 4,99E-03 |
| TMEM25 | ENSBTAG00000012940 | ENSBTAG00000012940 | 0,302 | 5,16E-03 |
| SMIM1 | ENSBTAG00000045754 | ENSBTAG00000045754 | 1,034 | 5,27E-03 |
| ELAPOR2 | ENSBTAG00000004023 | ENSBTAG00000004023 | 0,525 | 5,29E-03 |
| KNL1 | ENSBTAG00000053398 | ENSBTAG00000053398 | -0,661 | 5,38E-03 |
| FGF18 | ENSBTAG00000000128 | ENSBTAG00000000128 | 1,076 | 5,43E-03 |
| SPOCD1 | ENSBTAG00000015715 | ENSBTAG00000015715 | -0,371 | 5,52E-03 |
| TMEM151A | ENSBTAG00000020203 | ENSBTAG00000020203 | 0,679 | 5,56E-03 |
| RAD51AP1 | ENSBTAG00000040065 | ENSBTAG00000040065 | -0,917 | 5,61E-03 |
| ZNRF1 | ENSBTAG00000034689 | ENSBTAG00000034689 | -0,145 | 5,79E-03 |
| GALNT11 | ENSBTAG00000021260 | ENSBTAG00000021260 | 0,241 | 6,02E-03 |
| RGS7BP | ENSBTAG00000005169 | ENSBTAG00000005169 | 0,629 | 6,10E-03 |
| FAM133A | ENSBTAG00000011277 | ENSBTAG00000011277 | -1,564 | 6,30E-03 |
| KIF22 | ENSBTAG00000013669 | ENSBTAG00000013669 | -0,555 | 6,31E-03 |
| NIPAL1 | ENSBTAG00000009423 | ENSBTAG00000009423 | -1,008 | 6,31E-03 |
| GMPPA | ENSBTAG00000002995 | ENSBTAG00000002995 | -0,218 | 6,40E-03 |
| STK36 | ENSBTAG00000003819 | ENSBTAG00000003819 | 0,292 | 7,38E-03 |
| SDK1 | ENSBTAG00000004035 | ENSBTAG00000004035 | 0,575 | 7,61E-03 |
| RUVBL2 | ENSBTAG00000023417 | ENSBTAG00000023417 | -0,258 | 7,72E-03 |
| AKR1C3 | ENSBTAG00000022570 | ENSBTAG00000022570 | -0,786 | 7,96E-03 |
| SPAG5 | ENSBTAG00000013100 | ENSBTAG00000013100 | -0,633 | 7,98E-03 |
| RNF157 | ENSBTAG00000016240 | ENSBTAG00000016240 | -0,658 | 8,03E-03 |
| DHFR | ENSBTAG00000007681 | ENSBTAG00000007681 | -0,579 | 8,11E-03 |
| DISC1 | ENSBTAG00000050839 | ENSBTAG00000050839 | 0,614 | 8,24E-03 |
| TBC1D9 | ENSBTAG00000018951 | ENSBTAG00000018951 | 0,292 | 8,30E-03 |
| PITX1 | ENSBTAG00000004602 | ENSBTAG00000004602 | -0,526 | 8,52E-03 |
| CEP85 | ENSBTAG00000009579 | ENSBTAG00000009579 | -0,262 | 8,64E-03 |
| CKS1B | ENSBTAG00000024476 | ENSBTAG00000024476 | -0,346 | 8,75E-03 |
| ESCO2 | ENSBTAG00000006551 | ENSBTAG00000006551 | -0,711 | 8,79E-03 |
| PSMA4 | ENSBTAG00000014440 | ENSBTAG00000014440 | -0,229 | 8,83E-03 |
| KBTBD11 | ENSBTAG00000018284 | ENSBTAG00000018284 | 0,665 | 9,08E-03 |
| DDX39A | ENSBTAG00000021820 | ENSBTAG00000021820 | -0,262 | 9,13E-03 |
| FAXC | ENSBTAG00000044061 | ENSBTAG00000044061 | -0,517 | 9,14E-03 |
| TFPI | ENSBTAG00000049919 | ENSBTAG00000049919 | -0,466 | 9,17E-03 |
| PDE1C | ENSBTAG00000002739 | ENSBTAG00000002739 | 0,298 | 9,23E-03 |
| PRKN | ENSBTAG00000018996 | ENSBTAG00000018996 | 0,81 | 9,34E-03 |
| FAM149B1 | ENSBTAG00000019130 | ENSBTAG00000019130 | 0,273 | 9,38E-03 |
| HSPB7 | ENSBTAG00000055045 | ENSBTAG00000055045 | 0,504 | 9,52E-03 |
| NPY | ENSBTAG00000004503 | ENSBTAG00000004503 | -0,576 | 9,54E-03 |
| SMS | ENSBTAG00000018382 | ENSBTAG00000018382 | -0,18 | 9,54E-03 |
| NTNG2 | ENSBTAG00000007235 | ENSBTAG00000007235 | -0,437 | 9,58E-03 |
| FGF1 | ENSBTAG00000005198 | ENSBTAG00000005198 | -1,039 | 9,62E-03 |
| SETBP1 | ENSBTAG00000018088 | ENSBTAG00000018088 | -1,097 | 9,62E-03 |
| FBN1 | ENSBTAG00000002278 | ENSBTAG00000002278 | 0,272 | 9,64E-03 |
| MTM1 | ENSBTAG00000014138 | ENSBTAG00000014138 | 0,302 | 9,71E-03 |
| UBE2S | ENSBTAG00000009211 | ENSBTAG00000009211 | -0,397 | 9,73E-03 |
| SLIT3 | ENSBTAG00000017746 | ENSBTAG00000017746 | 0,302 | 9,79E-03 |
| PRAG1 | ENSBTAG00000000357 | ENSBTAG00000000357 | -0,31 | 9,83E-03 |
| PDZD4 | ENSBTAG00000021422 | ENSBTAG00000021422 | 0,684 | 9,87E-03 |
| APC2 | ENSBTAG00000015433 | ENSBTAG00000015433 | 0,393 | 9,88E-03 |
| WDR5 | ENSBTAG00000047031 | ENSBTAG00000047031 | -0,302 | 9,90E-03 |
| CEP55 | ENSBTAG00000005129 | ENSBTAG00000005129 | -0,592 | 9,96E-03 |
| TPX2 | ENSBTAG00000018775 | ENSBTAG00000018775 | -0,559 | 1,00E-02 |
| TMEM130 | ENSBTAG00000005679 | ENSBTAG00000005679 | 0,661 | 1,01E-02 |
| P2RX1 | ENSBTAG00000007169 | ENSBTAG00000007169 | -0,955 | 1,05E-02 |
| HIRIP3 | ENSBTAG00000005028 | ENSBTAG00000005028 | -0,389 | 1,07E-02 |
| SHROOM3 | ENSBTAG00000019633 | ENSBTAG00000019633 | 0,312 | 1,08E-02 |
| MARCHF2 | ENSBTAG00000021036 | ENSBTAG00000021036 | 0,293 | 1,09E-02 |
| NXT2 | ENSBTAG00000020739 | ENSBTAG00000020739 | -0,344 | 1,09E-02 |
| BAZ1A | ENSBTAG00000020164 | ENSBTAG00000020164 | -0,203 | 1,11E-02 |
| RALGPS1 | ENSBTAG00000023843 | ENSBTAG00000023843 | 0,344 | 1,11E-02 |
| TMX2 | ENSBTAG00000002404 | ENSBTAG00000002404 | 0,181 | 1,11E-02 |
| EFEMP1 | ENSBTAG00000017448 | ENSBTAG00000017448 | 0,186 | 1,12E-02 |
| TEDC2 | ENSBTAG00000024884 | ENSBTAG00000024884 | -0,594 | 1,14E-02 |
| C11orf87 | ENSBTAG00000050066 | ENSBTAG00000050066 | -1,302 | 1,15E-02 |
| BMP6 | ENSBTAG00000019234 | ENSBTAG00000019234 | 0,72 | 1,17E-02 |
| RAD54L | ENSBTAG00000019901 | ENSBTAG00000019901 | -0,575 | 1,17E-02 |
| HOXA3 | ENSBTAG00000008139 | ENSBTAG00000008139 | -0,323 | 1,18E-02 |
| GRAMD1B | ENSBTAG00000001410 | ENSBTAG00000001410 | 0,37 | 1,21E-02 |
| ZMYND12 | ENSBTAG00000012979 | ENSBTAG00000012979 | 0,65 | 1,21E-02 |
| KIF18B | ENSBTAG00000008088 | ENSBTAG00000008088 | -0,637 | 1,22E-02 |
| TGS1 | ENSBTAG00000005898 | ENSBTAG00000005898 | -0,347 | 1,22E-02 |
| ACD | ENSBTAG00000018903 | ENSBTAG00000018903 | -0,344 | 1,23E-02 |
| KRR1 | ENSBTAG00000011591 | ENSBTAG00000011591 | -0,171 | 1,24E-02 |
| CENPK | ENSBTAG00000044175 | ENSBTAG00000044175 | -0,574 | 1,25E-02 |
| SERPINE1 | ENSBTAG00000014465 | ENSBTAG00000014465 | 0,175 | 1,26E-02 |
| COQ8B | ENSBTAG00000019462 | ENSBTAG00000019462 | 0,244 | 1,27E-02 |
| DGCR2 | ENSBTAG00000000429 | ENSBTAG00000000429 | 0,271 | 1,28E-02 |
| GHR | ENSBTAG00000001335 | ENSBTAG00000001335 | -0,943 | 1,28E-02 |
| CELSR2 | ENSBTAG00000018804 | ENSBTAG00000018804 | 0,27 | 1,29E-02 |
| CKS2 | ENSBTAG00000001938 | ENSBTAG00000001938 | -0,467 | 1,30E-02 |
| PPIL2 | ENSBTAG00000021121 | ENSBTAG00000021121 | 0,23 | 1,30E-02 |
| PPP1R3E | ENSBTAG00000037679 | ENSBTAG00000037679 | 0,596 | 1,30E-02 |
| UPK3B | ENSBTAG00000021430 | ENSBTAG00000021430 | 1,225 | 1,31E-02 |
| KCNJ10 | ENSBTAG00000002414 | ENSBTAG00000002414 | 0,558 | 1,32E-02 |
| NR3C2 | ENSBTAG00000027182 | ENSBTAG00000027182 | 0,445 | 1,33E-02 |
| KRT16 | ENSBTAG00000033766 | ENSBTAG00000033766 | -0,78 | 1,36E-02 |
| COCH | ENSBTAG00000021844 | ENSBTAG00000021844 | -0,249 | 1,38E-02 |
| MMP11 | ENSBTAG00000006108 | ENSBTAG00000006108 | 0,407 | 1,39E-02 |
| ZBTB22 | ENSBTAG00000016829 | ENSBTAG00000016829 | 0,181 | 1,40E-02 |
| AKT1 | ENSBTAG00000017636 | ENSBTAG00000017636 | -0,121 | 1,41E-02 |
| DCLK2 | ENSBTAG00000008098 | ENSBTAG00000008098 | 0,289 | 1,41E-02 |
| DLGAP1 | ENSBTAG00000005372 | ENSBTAG00000005372 | 0,341 | 1,44E-02 |
| ALYREF | ENSBTAG00000008498 | ENSBTAG00000008498 | -0,375 | 1,45E-02 |
| FANCI | ENSBTAG00000009097 | ENSBTAG00000009097 | -0,382 | 1,46E-02 |
| DENND2A | ENSBTAG00000004799 | ENSBTAG00000004799 | -0,714 | 1,47E-02 |
| PAIP2B | ENSBTAG00000054656 | ENSBTAG00000054656 | -0,61 | 1,47E-02 |
| PIM1 | ENSBTAG00000000396 | ENSBTAG00000000396 | -0,228 | 1,47E-02 |
| PROS1 | ENSBTAG00000023652 | ENSBTAG00000023652 | 0,307 | 1,50E-02 |
| RNF44 | ENSBTAG00000017748 | ENSBTAG00000017748 | 0,176 | 1,51E-02 |
| CWF19L1 | ENSBTAG00000007594 | ENSBTAG00000007594 | -0,217 | 1,53E-02 |
| GCC2 | ENSBTAG00000016038 | ENSBTAG00000016038 | 0,228 | 1,54E-02 |
| NAV2 | ENSBTAG00000018431 | ENSBTAG00000018431 | 0,393 | 1,54E-02 |
| DDAH1 | ENSBTAG00000034776 | ENSBTAG00000034776 | 0,261 | 1,58E-02 |
| MOK | ENSBTAG00000007008 | ENSBTAG00000007008 | 0,539 | 1,58E-02 |
| GALNT17 | ENSBTAG00000008718 | ENSBTAG00000008718 | 0,267 | 1,60E-02 |
| ZRANB1 | ENSBTAG00000003395 | ENSBTAG00000003395 | 0,096 | 1,60E-02 |
| JAZF1 | ENSBTAG00000019024 | ENSBTAG00000019024 | 0,27 | 1,62E-02 |
| RBM48 | ENSBTAG00000018363 | ENSBTAG00000018363 | 0,525 | 1,62E-02 |
| CALB2 | ENSBTAG00000001075 | ENSBTAG00000001075 | -0,435 | 1,63E-02 |
| CCNB1 | ENSBTAG00000014239 | ENSBTAG00000014239 | -0,64 | 1,63E-02 |
| FCHO1 | ENSBTAG00000002136 | ENSBTAG00000002136 | 0,447 | 1,63E-02 |
| PKMYT1 | ENSBTAG00000004729 | ENSBTAG00000004729 | -0,679 | 1,64E-02 |
| TMC3 | ENSBTAG00000012166 | ENSBTAG00000012166 | 1,189 | 1,64E-02 |
| CLN6 | ENSBTAG00000005565 | ENSBTAG00000005565 | -0,316 | 1,66E-02 |
| KIF23 | ENSBTAG00000009983 | ENSBTAG00000009983 | -0,516 | 1,66E-02 |
| PPARGC1A | ENSBTAG00000017024 | ENSBTAG00000017024 | 0,514 | 1,66E-02 |
| NADSYN1 | ENSBTAG00000016470 | ENSBTAG00000016470 | 0,193 | 1,67E-02 |
| PDE4B | ENSBTAG00000008636 | ENSBTAG00000008636 | 0,67 | 1,67E-02 |
| ZDHHC14 | ENSBTAG00000018464 | ENSBTAG00000018464 | 0,218 | 1,67E-02 |
| DAB2 | ENSBTAG00000016152 | ENSBTAG00000016152 | 0,264 | 1,69E-02 |
| PRC1 | ENSBTAG00000018643 | ENSBTAG00000018643 | -0,581 | 1,70E-02 |
| HMMR | ENSBTAG00000014773 | ENSBTAG00000014773 | -0,556 | 1,72E-02 |
| TNFRSF1B | ENSBTAG00000024928 | ENSBTAG00000024928 | -1,326 | 1,72E-02 |
| ARRDC4 | ENSBTAG00000018252 | ENSBTAG00000018252 | 0,384 | 1,74E-02 |
| H1-4 | ENSBTAG00000047206 | ENSBTAG00000047206 | -2,877 | 1,75E-02 |
| NUF2 | ENSBTAG00000007247 | ENSBTAG00000007247 | -0,59 | 1,75E-02 |
| PRUNE2 | ENSBTAG00000053347 | ENSBTAG00000053347 | 0,419 | 1,75E-02 |
| NLGN3 | ENSBTAG00000007708 | ENSBTAG00000007708 | -0,615 | 1,76E-02 |
| PLEKHF1 | ENSBTAG00000008625 | ENSBTAG00000008625 | -0,491 | 1,76E-02 |
| B2M | ENSBTAG00000012330 | ENSBTAG00000012330 | -1,07 | 1,77E-02 |
| DEPDC1B | ENSBTAG00000017026 | ENSBTAG00000017026 | -0,683 | 1,77E-02 |
| CASD1 | ENSBTAG00000009109 | ENSBTAG00000009109 | 0,213 | 1,78E-02 |
| LHX9 | ENSBTAG00000013499 | ENSBTAG00000013499 | 0,4 | 1,78E-02 |
| RNF26 | ENSBTAG00000012562 | ENSBTAG00000012562 | -0,223 | 1,79E-02 |
| TENM4 | ENSBTAG00000007439 | ENSBTAG00000007439 | 0,378 | 1,80E-02 |
| CDCA7 | ENSBTAG00000003458 | ENSBTAG00000003458 | -0,826 | 1,82E-02 |
| KIAA0586 | ENSBTAG00000004631 | ENSBTAG00000004631 | -0,249 | 1,82E-02 |
| KIF20B | ENSBTAG00000005708 | ENSBTAG00000005708 | -0,413 | 1,83E-02 |
| AURKA | ENSBTAG00000013009 | ENSBTAG00000013009 | -0,556 | 1,85E-02 |
| PLSCR4 | ENSBTAG00000011986 | ENSBTAG00000011986 | 0,272 | 1,85E-02 |
| ZNF275 | ENSBTAG00000020185 | ENSBTAG00000020185 | 0,377 | 1,85E-02 |
| PSIP1 | ENSBTAG00000007872 | ENSBTAG00000007872 | -0,191 | 1,86E-02 |
| SNRPA | ENSBTAG00000009077 | ENSBTAG00000009077 | -0,25 | 1,86E-02 |
| DNTTIP2 | ENSBTAG00000047679 | ENSBTAG00000047679 | -0,177 | 1,88E-02 |
| NRBP2 | ENSBTAG00000008079 | ENSBTAG00000008079 | 0,257 | 1,88E-02 |
| NUDCD1 | ENSBTAG00000007386 | ENSBTAG00000007386 | -0,224 | 1,89E-02 |
| REEP4 | ENSBTAG00000009504 | ENSBTAG00000009504 | -0,333 | 1,89E-02 |
| CHTF18 | ENSBTAG00000019743 | ENSBTAG00000019743 | -0,476 | 1,90E-02 |
| INCENP | ENSBTAG00000050061 | ENSBTAG00000050061 | -0,445 | 1,91E-02 |
| TGFBI | ENSBTAG00000009513 | ENSBTAG00000009513 | -0,274 | 1,91E-02 |
| FHIP2A | ENSBTAG00000003062 | ENSBTAG00000003062 | 0,208 | 1,92E-02 |
| GNG2 | ENSBTAG00000003043 | ENSBTAG00000003043 | -0,46 | 1,92E-02 |
| CDCA8 | ENSBTAG00000014326 | ENSBTAG00000014326 | -0,629 | 1,93E-02 |
| NCAPD2 | ENSBTAG00000014730 | ENSBTAG00000014730 | -0,344 | 1,93E-02 |
| CDC20 | ENSBTAG00000009819 | ENSBTAG00000009819 | -0,743 | 1,94E-02 |
| MEDAG | ENSBTAG00000008271 | ENSBTAG00000008271 | -0,763 | 1,94E-02 |
| MYH15 | ENSBTAG00000018399 | ENSBTAG00000018399 | -0,781 | 1,94E-02 |
| PCSK5 | ENSBTAG00000008101 | ENSBTAG00000008101 | 0,324 | 1,94E-02 |
| TMEM232 | ENSBTAG00000030198 | ENSBTAG00000030198 | 1,341 | 1,94E-02 |
| NASP | ENSBTAG00000015346 | ENSBTAG00000015346 | -0,295 | 1,95E-02 |
| DYNLRB2 | ENSBTAG00000017752 | ENSBTAG00000017752 | 0,817 | 1,98E-02 |
| PBK | ENSBTAG00000021069 | ENSBTAG00000021069 | -0,579 | 1,98E-02 |
| SFRP1 | ENSBTAG00000027625 | ENSBTAG00000027625 | 0,359 | 1,98E-02 |
| TNFRSF21 | ENSBTAG00000020054 | ENSBTAG00000020054 | 0,26 | 1,99E-02 |
| LTBP2 | ENSBTAG00000021957 | ENSBTAG00000021957 | 0,254 | 2,00E-02 |
| AK7 | ENSBTAG00000016854 | ENSBTAG00000016854 | 1,812 | 2,03E-02 |
| RSPH1 | ENSBTAG00000021284 | ENSBTAG00000021284 | 2,437 | 2,03E-02 |
| KPNA2 | ENSBTAG00000012225 | ENSBTAG00000012225 | -0,351 | 2,04E-02 |
| BCAS2 | ENSBTAG00000016705 | ENSBTAG00000016705 | -0,173 | 2,05E-02 |
| BLM | ENSBTAG00000020301 | ENSBTAG00000020301 | -0,516 | 2,05E-02 |
| PCLO | ENSBTAG00000017350 | ENSBTAG00000017350 | 0,402 | 2,05E-02 |
| SNRPF | ENSBTAG00000016271 | ENSBTAG00000016271 | -0,259 | 2,05E-02 |
| CCNA2 | ENSBTAG00000004943 | ENSBTAG00000004943 | -0,509 | 2,08E-02 |
| SRSF4 | ENSBTAG00000013309 | ENSBTAG00000013309 | -0,156 | 2,09E-02 |
| PIMREG | ENSBTAG00000002981 | ENSBTAG00000002981 | -0,507 | 2,10E-02 |
| UGDH | ENSBTAG00000014521 | ENSBTAG00000014521 | -0,213 | 2,11E-02 |
| CCNL2 | ENSBTAG00000004873 | ENSBTAG00000004873 | 0,275 | 2,12E-02 |
| SMC4 | ENSBTAG00000005862 | ENSBTAG00000005862 | -0,381 | 2,12E-02 |
| PLK4 | ENSBTAG00000039552 | ENSBTAG00000039552 | -0,542 | 2,13E-02 |
| CPM | ENSBTAG00000013496 | ENSBTAG00000013496 | -1,132 | 2,14E-02 |
| EPHA2 | ENSBTAG00000000815 | ENSBTAG00000000815 | -0,198 | 2,14E-02 |
| KIF26A | ENSBTAG00000021904 | ENSBTAG00000021904 | 0,927 | 2,14E-02 |
| ESPL1 | ENSBTAG00000008934 | ENSBTAG00000008934 | -0,588 | 2,15E-02 |
| MKI67 | ENSBTAG00000002444 | ENSBTAG00000002444 | -0,595 | 2,15E-02 |
| SPATA25 | ENSBTAG00000000960 | ENSBTAG00000000960 | 1,783 | 2,16E-02 |
| RGS14 | ENSBTAG00000008497 | ENSBTAG00000008497 | 0,272 | 2,17E-02 |
| MGAT4A | ENSBTAG00000010388 | ENSBTAG00000010388 | 0,627 | 2,18E-02 |
| ECT2 | ENSBTAG00000023814 | ENSBTAG00000023814 | -0,289 | 2,20E-02 |
| KIFC1 | ENSBTAG00000001631 | ENSBTAG00000001631 | -0,616 | 2,20E-02 |
| NUCKS1 | ENSBTAG00000008001 | ENSBTAG00000008001 | -0,249 | 2,20E-02 |
| OPRL1 | ENSBTAG00000049473 | ENSBTAG00000049473 | 0,702 | 2,20E-02 |
| RHBDD2 | ENSBTAG00000005089 | ENSBTAG00000005089 | 0,215 | 2,21E-02 |
| KLHDC8A | ENSBTAG00000015197 | ENSBTAG00000015197 | 0,441 | 2,22E-02 |
| NEK2 | ENSBTAG00000009618 | ENSBTAG00000009618 | -0,525 | 2,24E-02 |
| TMEM132A | ENSBTAG00000018364 | ENSBTAG00000018364 | 0,291 | 2,24E-02 |
| RAD51 | ENSBTAG00000002918 | ENSBTAG00000002918 | -0,516 | 2,25E-02 |
| PHF19 | ENSBTAG00000016958 | ENSBTAG00000016958 | -0,429 | 2,26E-02 |
| TESC | ENSBTAG00000044078 | ENSBTAG00000044078 | -0,322 | 2,26E-02 |
| MRPS22 | ENSBTAG00000047608 | ENSBTAG00000047608 | -0,242 | 2,27E-02 |
| GADD45G | ENSBTAG00000003033 | ENSBTAG00000003033 | 0,829 | 2,28E-02 |
| GARNL3 | ENSBTAG00000006948 | ENSBTAG00000006948 | 0,425 | 2,29E-02 |
| CLDND1 | ENSBTAG00000020996 | ENSBTAG00000020996 | -0,122 | 2,30E-02 |
| H2AZ1 | ENSBTAG00000004428 | ENSBTAG00000004428 | -0,37 | 2,30E-02 |
| PLPP4 | ENSBTAG00000032106 | ENSBTAG00000032106 | -0,325 | 2,33E-02 |
| MYO1D | ENSBTAG00000015527 | ENSBTAG00000015527 | 0,273 | 2,35E-02 |
| SHMT1 | ENSBTAG00000017094 | ENSBTAG00000017094 | -0,402 | 2,37E-02 |
| TMEM38A | ENSBTAG00000013614 | ENSBTAG00000013614 | 0,31 | 2,37E-02 |
| EXO1 | ENSBTAG00000009396 | ENSBTAG00000009396 | -0,634 | 2,38E-02 |
| SEL1L | ENSBTAG00000008083 | ENSBTAG00000008083 | 0,217 | 2,38E-02 |
| KIF4A | ENSBTAG00000012861 | ENSBTAG00000012861 | -0,525 | 2,39E-02 |
| BBS9 | ENSBTAG00000006528 | ENSBTAG00000006528 | 0,203 | 2,42E-02 |
| MTMR10 | ENSBTAG00000014422 | ENSBTAG00000014422 | 0,314 | 2,42E-02 |
| STOX1 | ENSBTAG00000019028 | ENSBTAG00000019028 | -1,314 | 2,42E-02 |
| SH3D19 | ENSBTAG00000016813 | ENSBTAG00000016813 | 0,259 | 2,43E-02 |
| ZNF516 | ENSBTAG00000004650 | ENSBTAG00000004650 | 0,202 | 2,43E-02 |
| MOCOS | ENSBTAG00000012252 | ENSBTAG00000012252 | 0,356 | 2,44E-02 |
| AKAP6 | ENSBTAG00000017719 | ENSBTAG00000017719 | 0,495 | 2,45E-02 |
| HTR2A | ENSBTAG00000013498 | ENSBTAG00000013498 | 0,563 | 2,45E-02 |
| PRUNE2 | ENSBTAG00000012991 | ENSBTAG00000012991 | 0,416 | 2,45E-02 |
| RNF114 | ENSBTAG00000027317 | ENSBTAG00000027317 | -0,14 | 2,45E-02 |
| MDH1B | ENSBTAG00000016192 | ENSBTAG00000016192 | 0,869 | 2,46E-02 |
| METAP2 | ENSBTAG00000019944 | ENSBTAG00000019944 | -0,167 | 2,47E-02 |
| DNAH17 | ENSBTAG00000000920 | ENSBTAG00000000920 | 2,21 | 2,48E-02 |
| ANKRD6 | ENSBTAG00000003455 | ENSBTAG00000003455 | 0,215 | 2,49E-02 |
| C5orf63 | ENSBTAG00000017835 | ENSBTAG00000017835 | 0,651 | 2,50E-02 |
| PTPRN2 | ENSBTAG00000004958 | ENSBTAG00000004958 | -1,038 | 2,50E-02 |
| ALDH2 | ENSBTAG00000008743 | ENSBTAG00000008743 | 0,315 | 2,51E-02 |
| IRF8 | ENSBTAG00000017824 | ENSBTAG00000017824 | -0,988 | 2,51E-02 |
| TNRC6A | ENSBTAG00000017999 | ENSBTAG00000017999 | -0,207 | 2,53E-02 |
| GNAI3 | ENSBTAG00000013016 | ENSBTAG00000013016 | -0,132 | 2,54E-02 |
| IFT80 | ENSBTAG00000031572 | ENSBTAG00000031572 | 0,171 | 2,55E-02 |
| TFAP2A | ENSBTAG00000001250 | ENSBTAG00000001250 | -0,327 | 2,57E-02 |
| KRTAP1-1 | ENSBTAG00000049202 | ENSBTAG00000049202 | -1,32 | 2,58E-02 |
| CNIH3 | ENSBTAG00000053199 | ENSBTAG00000053199 | -0,23 | 2,61E-02 |
| PSMC1 | ENSBTAG00000005426 | ENSBTAG00000005426 | -0,131 | 2,62E-02 |
| EFR3B | ENSBTAG00000007888 | ENSBTAG00000007888 | 0,726 | 2,64E-02 |
| POLE3 | ENSBTAG00000000252 | ENSBTAG00000000252 | -0,175 | 2,65E-02 |
| NR2F2 | ENSBTAG00000018007 | ENSBTAG00000018007 | -0,264 | 2,67E-02 |
| Anapc15 | ENSBTAG00000002999 | ENSBTAG00000002999 | -0,27 | 2,69E-02 |
| EXOSC8 | ENSBTAG00000011855 | ENSBTAG00000011855 | -0,279 | 2,69E-02 |
| SPECC1L | ENSBTAG00000021656 | ENSBTAG00000021656 | 0,264 | 2,70E-02 |
| JPT1 | ENSBTAG00000030170 | ENSBTAG00000030170 | -0,224 | 2,73E-02 |
| FOXL2 | ENSBTAG00000031277 | ENSBTAG00000031277 | -0,312 | 2,75E-02 |
| FTSJ1 | ENSBTAG00000031829 | ENSBTAG00000031829 | -0,451 | 2,75E-02 |
| MICAL3 | ENSBTAG00000048271 | ENSBTAG00000048271 | 0,245 | 2,76E-02 |
| CENPF | ENSBTAG00000024449 | ENSBTAG00000024449 | -0,425 | 2,77E-02 |
| SPTB | ENSBTAG00000004732 | ENSBTAG00000004732 | 0,72 | 2,77E-02 |
| CYB561 | ENSBTAG00000020810 | ENSBTAG00000020810 | 0,408 | 2,79E-02 |
| ZNF629 | ENSBTAG00000026307 | ENSBTAG00000026307 | 0,169 | 2,84E-02 |
| SLC7A7 | ENSBTAG00000014821 | ENSBTAG00000014821 | 0,658 | 2,85E-02 |
| KIF11 | ENSBTAG00000009383 | ENSBTAG00000009383 | -0,46 | 2,86E-02 |
| RNASEH2A | ENSBTAG00000009661 | ENSBTAG00000009661 | -0,456 | 2,86E-02 |
| DAAM1 | ENSBTAG00000005984 | ENSBTAG00000005984 | 0,188 | 2,89E-02 |
| FARSA | ENSBTAG00000021374 | ENSBTAG00000021374 | -0,189 | 2,89E-02 |
| RWDD2A | ENSBTAG00000015911 | ENSBTAG00000015911 | 0,268 | 2,90E-02 |
| ARHGAP11A | ENSBTAG00000020100 | ENSBTAG00000020100 | -0,45 | 2,92E-02 |
| PDE12 | ENSBTAG00000015563 | ENSBTAG00000015563 | -0,204 | 2,92E-02 |
| SEMA3D | ENSBTAG00000024394 | ENSBTAG00000024394 | 0,543 | 2,92E-02 |
| ATRIP | ENSBTAG00000008399 | ENSBTAG00000008399 | -0,232 | 2,93E-02 |
| CAMKK2 | ENSBTAG00000010815 | ENSBTAG00000010815 | 0,161 | 2,94E-02 |
| CBX8 | ENSBTAG00000009107 | ENSBTAG00000009107 | -0,349 | 2,95E-02 |
| PDCD4 | ENSBTAG00000019434 | ENSBTAG00000019434 | 0,17 | 2,95E-02 |
| FAM83D | ENSBTAG00000000660 | ENSBTAG00000000660 | -0,572 | 2,97E-02 |
| ZKSCAN1 | ENSBTAG00000016757 | ENSBTAG00000016757 | 0,363 | 2,97E-02 |
| DOK4 | ENSBTAG00000001857 | ENSBTAG00000001857 | 0,213 | 3,00E-02 |
| HNRNPA2B1 | ENSBTAG00000005726 | ENSBTAG00000005726 | -0,265 | 3,00E-02 |
| PDE5A | ENSBTAG00000024888 | ENSBTAG00000024888 | 0,208 | 3,00E-02 |
| WIPF3 | ENSBTAG00000016220 | ENSBTAG00000016220 | 0,311 | 3,01E-02 |
| PRMT5 | ENSBTAG00000010890 | ENSBTAG00000010890 | -0,135 | 3,02E-02 |
| TMEM129 | ENSBTAG00000011042 | ENSBTAG00000011042 | 0,205 | 3,02E-02 |
| ENTPD2 | ENSBTAG00000048125 | ENSBTAG00000048125 | 2,856 | 3,04E-02 |
| PLK2 | ENSBTAG00000016771 | ENSBTAG00000016771 | -0,361 | 3,05E-02 |
| UBE2C | ENSBTAG00000016746 | ENSBTAG00000016746 | -0,544 | 3,05E-02 |
| EIF4A3 | ENSBTAG00000016023 | ENSBTAG00000016023 | -0,23 | 3,06E-02 |
| HNRNPM | ENSBTAG00000021039 | ENSBTAG00000021039 | -0,272 | 3,06E-02 |
| SLITRK4 | ENSBTAG00000011780 | ENSBTAG00000011780 | 0,497 | 3,06E-02 |
| PNP | ENSBTAG00000037452 | ENSBTAG00000037452 | -0,237 | 3,07E-02 |
| TPP1 | ENSBTAG00000015403 | ENSBTAG00000015403 | 0,239 | 3,09E-02 |
| VPS41 | ENSBTAG00000007305 | ENSBTAG00000007305 | 0,191 | 3,09E-02 |
| NUDC | ENSBTAG00000004416 | ENSBTAG00000004416 | -0,219 | 3,11E-02 |
| POU4F3 | ENSBTAG00000018817 | ENSBTAG00000018817 | -2,795 | 3,11E-02 |
| ZSCAN26 | ENSBTAG00000031869 | ENSBTAG00000031869 | 0,241 | 3,11E-02 |
| GPSM2 | ENSBTAG00000017124 | ENSBTAG00000017124 | -0,322 | 3,12E-02 |
| FBL | ENSBTAG00000002579 | ENSBTAG00000002579 | -0,264 | 3,13E-02 |
| B4GALT1 | ENSBTAG00000015249 | ENSBTAG00000015249 | 0,112 | 3,17E-02 |
| HSPG2 | ENSBTAG00000017122 | ENSBTAG00000017122 | 0,211 | 3,17E-02 |
| KIF20A | ENSBTAG00000008758 | ENSBTAG00000008758 | -0,654 | 3,18E-02 |
| CDK5RAP1 | ENSBTAG00000018535 | ENSBTAG00000018535 | -0,223 | 3,19E-02 |
| HMGB1 | ENSBTAG00000018103 | ENSBTAG00000018103 | -0,275 | 3,19E-02 |
| CPQ | ENSBTAG00000011908 | ENSBTAG00000011908 | 0,175 | 3,20E-02 |
| ANKIB1 | ENSBTAG00000034936 | ENSBTAG00000034936 | 0,222 | 3,21E-02 |
| KLC3 | ENSBTAG00000002070 | ENSBTAG00000002070 | -0,317 | 3,21E-02 |
| PLPPR4 | ENSBTAG00000019031 | ENSBTAG00000019031 | -0,627 | 3,21E-02 |
| SLC4A8 | ENSBTAG00000017950 | ENSBTAG00000017950 | -0,402 | 3,23E-02 |
| SPAG16 | ENSBTAG00000018549 | ENSBTAG00000018549 | 0,282 | 3,23E-02 |
| ATAD5 | ENSBTAG00000018383 | ENSBTAG00000018383 | -0,571 | 3,25E-02 |
| FANCD2 | ENSBTAG00000010077 | ENSBTAG00000010077 | -0,478 | 3,25E-02 |
| DDX52 | ENSBTAG00000010313 | ENSBTAG00000010313 | -0,127 | 3,26E-02 |
| Nrgn | ENSBTAG00000001474 | ENSBTAG00000001474 | -0,699 | 3,26E-02 |
| ZFYVE26 | ENSBTAG00000014334 | ENSBTAG00000014334 | 0,175 | 3,27E-02 |
| ERCC6L | ENSBTAG00000005607 | ENSBTAG00000005607 | -0,561 | 3,29E-02 |
| ATP1B1 | ENSBTAG00000002688 | ENSBTAG00000002688 | 0,304 | 3,30E-02 |
| SUPT3H | ENSBTAG00000032887 | ENSBTAG00000032887 | -0,196 | 3,30E-02 |
| ARF6 | ENSBTAG00000045980 | ENSBTAG00000045980 | -0,15 | 3,32E-02 |
| AATF | ENSBTAG00000021927 | ENSBTAG00000021927 | -0,238 | 3,33E-02 |
| POGLUT1 | ENSBTAG00000008562 | ENSBTAG00000008562 | 0,216 | 3,33E-02 |
| TXNIP | ENSBTAG00000020060 | ENSBTAG00000020060 | 0,143 | 3,34E-02 |
| TAX1BP1 | ENSBTAG00000019020 | ENSBTAG00000019020 | 0,097 | 3,35E-02 |
| TIMM23 | ENSBTAG00000011694 | ENSBTAG00000011694 | -0,145 | 3,35E-02 |
| DLGAP5 | ENSBTAG00000002331 | ENSBTAG00000002331 | -0,631 | 3,36E-02 |
| MCL1 | ENSBTAG00000015154 | ENSBTAG00000015154 | 0,174 | 3,36E-02 |
| NEIL3 | ENSBTAG00000005825 | ENSBTAG00000005825 | -0,613 | 3,37E-02 |
| AGTPBP1 | ENSBTAG00000001257 | ENSBTAG00000001257 | 0,235 | 3,38E-02 |
| KIF2C | ENSBTAG00000015280 | ENSBTAG00000015280 | -0,646 | 3,39E-02 |
| KRTCAP3 | ENSBTAG00000018156 | ENSBTAG00000018156 | 1,51 | 3,39E-02 |
| PCLAF | ENSBTAG00000039462 | ENSBTAG00000039462 | -0,545 | 3,39E-02 |
| SOX30 | ENSBTAG00000020491 | ENSBTAG00000020491 | -2,44 | 3,39E-02 |
| SERINC2 | ENSBTAG00000035081 | ENSBTAG00000035081 | 0,295 | 3,40E-02 |
| CDK5R1 | ENSBTAG00000004475 | ENSBTAG00000004475 | -0,388 | 3,41E-02 |
| MANF | ENSBTAG00000031797 | ENSBTAG00000031797 | -0,248 | 3,42E-02 |
| MEGF6 | ENSBTAG00000020839 | ENSBTAG00000020839 | 0,479 | 3,42E-02 |
| C3orf62 | ENSBTAG00000011894 | ENSBTAG00000011894 | 0,557 | 3,43E-02 |
| PPARA | ENSBTAG00000008063 | ENSBTAG00000008063 | 0,462 | 3,43E-02 |
| CENPM | ENSBTAG00000009956 | ENSBTAG00000009956 | -0,664 | 3,45E-02 |
| FOXF2 | ENSBTAG00000026896 | ENSBTAG00000026896 | -0,545 | 3,45E-02 |
| H2AC12 | ENSBTAG00000054548 | ENSBTAG00000054548 | -0,497 | 3,45E-02 |
| NGEF | ENSBTAG00000008942 | ENSBTAG00000008942 | -0,491 | 3,45E-02 |
| FANCM | ENSBTAG00000002612 | ENSBTAG00000002612 | -0,371 | 3,46E-02 |
| APLP2 | ENSBTAG00000016168 | ENSBTAG00000016168 | 0,156 | 3,47E-02 |
| CACNA2D1 | ENSBTAG00000020569 | ENSBTAG00000020569 | 0,171 | 3,48E-02 |
| TMEM237 | ENSBTAG00000017437 | ENSBTAG00000017437 | -0,138 | 3,48E-02 |
| SLC37A3 | ENSBTAG00000012239 | ENSBTAG00000012239 | 0,314 | 3,49E-02 |
| CBR4 | ENSBTAG00000022013 | ENSBTAG00000022013 | 0,279 | 3,50E-02 |
| UHRF1 | ENSBTAG00000002224 | ENSBTAG00000002224 | -0,608 | 3,50E-02 |
| ZNF706 | ENSBTAG00000008054 | ENSBTAG00000008054 | -0,273 | 3,50E-02 |
| CENPI | ENSBTAG00000021254 | ENSBTAG00000021254 | -0,501 | 3,51E-02 |
| C6orf136 | ENSBTAG00000006958 | ENSBTAG00000006958 | -0,33 | 3,52E-02 |
| MBOAT1 | ENSBTAG00000016519 | ENSBTAG00000016519 | -0,336 | 3,52E-02 |
| ADM | ENSBTAG00000021048 | ENSBTAG00000021048 | -0,495 | 3,53E-02 |
| SOSTDC1 | ENSBTAG00000005377 | ENSBTAG00000005377 | -3,31 | 3,53E-02 |
| HARS1 | ENSBTAG00000018847 | ENSBTAG00000018847 | -0,125 | 3,54E-02 |
| RHBDL3 | ENSBTAG00000020992 | ENSBTAG00000020992 | -1,041 | 3,54E-02 |
| IDUA | ENSBTAG00000007002 | ENSBTAG00000007002 | 0,268 | 3,55E-02 |
| RNF216 | ENSBTAG00000017175 | ENSBTAG00000017175 | 0,116 | 3,55E-02 |
| ARMCX4 | ENSBTAG00000052983 | ENSBTAG00000052983 | -0,285 | 3,56E-02 |
| FAM110C | ENSBTAG00000051168 | ENSBTAG00000051168 | 0,524 | 3,56E-02 |
| AXL | ENSBTAG00000003166 | ENSBTAG00000003166 | -0,205 | 3,57E-02 |
| HSPBAP1 | ENSBTAG00000005066 | ENSBTAG00000005066 | 0,324 | 3,57E-02 |
| EVC2 | ENSBTAG00000004277 | ENSBTAG00000004277 | 0,333 | 3,58E-02 |
| SUV39H1 | ENSBTAG00000004706 | ENSBTAG00000004706 | -0,381 | 3,58E-02 |
| BCL2 | ENSBTAG00000019302 | ENSBTAG00000019302 | 0,652 | 3,59E-02 |
| LONRF1 | ENSBTAG00000020084 | ENSBTAG00000020084 | 0,203 | 3,59E-02 |
| SCLY | ENSBTAG00000017826 | ENSBTAG00000017826 | 0,494 | 3,59E-02 |
| UBR7 | ENSBTAG00000004310 | ENSBTAG00000004310 | -0,161 | 3,60E-02 |
| EXO5 | ENSBTAG00000000532 | ENSBTAG00000000532 | -0,442 | 3,61E-02 |
| KCNA2 | ENSBTAG00000015459 | ENSBTAG00000015459 | -3,406 | 3,62E-02 |
| SKA3 | ENSBTAG00000003314 | ENSBTAG00000003314 | -0,561 | 3,62E-02 |
| CACYBP | ENSBTAG00000001107 | ENSBTAG00000001107 | -0,208 | 3,63E-02 |
| ST6GALNAC5 | ENSBTAG00000007309 | ENSBTAG00000007309 | -0,746 | 3,63E-02 |
| MEIS2 | ENSBTAG00000003172 | ENSBTAG00000003172 | -0,264 | 3,67E-02 |
| GPR22 | ENSBTAG00000046917 | ENSBTAG00000046917 | 4,079 | 3,68E-02 |
| ERO1A | ENSBTAG00000015716 | ENSBTAG00000015716 | -0,163 | 3,69E-02 |
| FOXM1 | ENSBTAG00000015875 | ENSBTAG00000015875 | -0,374 | 3,69E-02 |
| PCDHA13 | ENSBTAG00000053546 | ENSBTAG00000053546 | 0,194 | 3,70E-02 |
| ANLN | ENSBTAG00000009218 | ENSBTAG00000009218 | -0,528 | 3,71E-02 |
| NCAPG2 | ENSBTAG00000016131 | ENSBTAG00000016131 | -0,398 | 3,71E-02 |
| DLL4 | ENSBTAG00000010361 | ENSBTAG00000010361 | -1,565 | 3,72E-02 |
| PCNA | ENSBTAG00000006065 | ENSBTAG00000006065 | -0,407 | 3,72E-02 |
| RGS5 | ENSBTAG00000016341 | ENSBTAG00000016341 | 0,266 | 3,73E-02 |
| SBSPON | ENSBTAG00000017249 | ENSBTAG00000017249 | 0,342 | 3,73E-02 |
| CPNE2 | ENSBTAG00000002233 | ENSBTAG00000002233 | -0,14 | 3,74E-02 |
| MSI1 | ENSBTAG00000009800 | ENSBTAG00000009800 | -0,633 | 3,74E-02 |
| PHYH | ENSBTAG00000007700 | ENSBTAG00000007700 | 0,225 | 3,75E-02 |
| GMPS | ENSBTAG00000013013 | ENSBTAG00000013013 | -0,199 | 3,76E-02 |
| DNMT1 | ENSBTAG00000002736 | ENSBTAG00000002736 | -0,262 | 3,77E-02 |
| ALPL | ENSBTAG00000008951 | ENSBTAG00000008951 | 1,046 | 3,78E-02 |
| FAM72A | ENSBTAG00000020227 | ENSBTAG00000020227 | -0,529 | 3,78E-02 |
| DEPDC1 | ENSBTAG00000001343 | ENSBTAG00000001343 | -0,515 | 3,80E-02 |
| PWWP3A | ENSBTAG00000030839 | ENSBTAG00000030839 | 0,26 | 3,80E-02 |
| SMU1 | ENSBTAG00000019564 | ENSBTAG00000019564 | -0,105 | 3,80E-02 |
| VAT1 | ENSBTAG00000007390 | ENSBTAG00000007390 | 0,144 | 3,81E-02 |
| ARL8B | ENSBTAG00000012902 | ENSBTAG00000012902 | 0,108 | 3,82E-02 |
| NDC80 | ENSBTAG00000021673 | ENSBTAG00000021673 | -0,523 | 3,82E-02 |
| SHROOM4 | ENSBTAG00000002996 | ENSBTAG00000002996 | 0,371 | 3,82E-02 |
| TIMELESS | ENSBTAG00000020459 | ENSBTAG00000020459 | -0,371 | 3,84E-02 |
| GADD45A | ENSBTAG00000013860 | ENSBTAG00000013860 | -0,194 | 3,86E-02 |
| MELK | ENSBTAG00000021686 | ENSBTAG00000021686 | -0,404 | 3,86E-02 |
| METTL1 | ENSBTAG00000016908 | ENSBTAG00000016908 | -0,237 | 3,86E-02 |
| FILIP1L | ENSBTAG00000013664 | ENSBTAG00000013664 | 0,153 | 3,87E-02 |
| HARBI1 | ENSBTAG00000004639 | ENSBTAG00000004639 | 0,329 | 3,89E-02 |
| HOXC10 | ENSBTAG00000003278 | ENSBTAG00000003278 | 0,273 | 3,90E-02 |
| DHH | ENSBTAG00000000124 | ENSBTAG00000000124 | 3,43 | 3,91E-02 |
| RCN3 | ENSBTAG00000021799 | ENSBTAG00000021799 | -0,306 | 3,91E-02 |
| SKA1 | ENSBTAG00000018216 | ENSBTAG00000018216 | -0,661 | 3,93E-02 |
| MMP24 | ENSBTAG00000010024 | ENSBTAG00000010024 | 0,267 | 3,94E-02 |
| ARRB1 | ENSBTAG00000020485 | ENSBTAG00000020485 | -0,237 | 3,97E-02 |
| DLAT | ENSBTAG00000010709 | ENSBTAG00000010709 | -0,144 | 3,97E-02 |
| MSH6 | ENSBTAG00000001424 | ENSBTAG00000001424 | -0,183 | 3,97E-02 |
| SFPQ | ENSBTAG00000016328 | ENSBTAG00000016328 | -0,212 | 3,97E-02 |
| CCDC150 | ENSBTAG00000017695 | ENSBTAG00000017695 | -0,522 | 3,98E-02 |
| LZTR1 | ENSBTAG00000011292 | ENSBTAG00000011292 | 0,186 | 3,98E-02 |
| GBP2 | ENSBTAG00000002416 | ENSBTAG00000002416 | -0,667 | 3,99E-02 |
| MBTD1 | ENSBTAG00000009407 | ENSBTAG00000009407 | 0,147 | 3,99E-02 |
| SLC2A8 | ENSBTAG00000006957 | ENSBTAG00000006957 | 0,407 | 3,99E-02 |
| Stra6l | ENSBTAG00000040034 | ENSBTAG00000040034 | 0,6 | 4,01E-02 |
| ZNF25 | ENSBTAG00000038178 | ENSBTAG00000038178 | 0,277 | 4,01E-02 |
| ARHGEF19 | ENSBTAG00000005670 | ENSBTAG00000005670 | -0,322 | 4,03E-02 |
| ATAD1 | ENSBTAG00000000806 | ENSBTAG00000000806 | 0,115 | 4,03E-02 |
| TUBA1A | ENSBTAG00000001489 | ENSBTAG00000001489 | -0,247 | 4,03E-02 |
| GSPT2 | ENSBTAG00000013311 | ENSBTAG00000013311 | -0,176 | 4,05E-02 |
| POLD2 | ENSBTAG00000012241 | ENSBTAG00000012241 | -0,193 | 4,05E-02 |
| ADAMTS15 | ENSBTAG00000016857 | ENSBTAG00000016857 | 0,328 | 4,06E-02 |
| NICN1 | ENSBTAG00000000387 | ENSBTAG00000000387 | 0,248 | 4,14E-02 |
| TOP2A | ENSBTAG00000019262 | ENSBTAG00000019262 | -0,539 | 4,14E-02 |
| AFAP1L2 | ENSBTAG00000004895 | ENSBTAG00000004895 | -0,609 | 4,15E-02 |
| CARNS1 | ENSBTAG00000017690 | ENSBTAG00000017690 | 0,547 | 4,15E-02 |
| FAM98B | ENSBTAG00000021870 | ENSBTAG00000021870 | -0,221 | 4,16E-02 |
| SHC4 | ENSBTAG00000022583 | ENSBTAG00000022583 | 0,474 | 4,17E-02 |
| SHCBP1 | ENSBTAG00000033441 | ENSBTAG00000033441 | -0,449 | 4,17E-02 |
| TMEM150A | ENSBTAG00000018289 | ENSBTAG00000018289 | 0,265 | 4,17E-02 |
| PGBD5 | ENSBTAG00000027205 | ENSBTAG00000027205 | -3,458 | 4,19E-02 |
| NEO1 | ENSBTAG00000004990 | ENSBTAG00000004990 | 0,267 | 4,21E-02 |
| RCOR3 | ENSBTAG00000007110 | ENSBTAG00000007110 | 0,164 | 4,21E-02 |
| DIAPH3 | ENSBTAG00000012443 | ENSBTAG00000012443 | -0,285 | 4,22E-02 |
| PSMD2 | ENSBTAG00000005660 | ENSBTAG00000005660 | -0,147 | 4,22E-02 |
| Chn2 | ENSBTAG00000020931 | ENSBTAG00000020931 | -0,269 | 4,25E-02 |
| WHRN | ENSBTAG00000010107 | ENSBTAG00000010107 | 0,439 | 4,25E-02 |
| H2AJ | ENSBTAG00000030503 | ENSBTAG00000030503 | -0,366 | 4,27E-02 |
| KCNB1 | ENSBTAG00000027320 | ENSBTAG00000027320 | 0,768 | 4,27E-02 |
| STX3 | ENSBTAG00000012820 | ENSBTAG00000012820 | 0,259 | 4,27E-02 |
| ERBB4 | ENSBTAG00000012647 | ENSBTAG00000012647 | 0,602 | 4,28E-02 |
| STON1 | ENSBTAG00000004168 | ENSBTAG00000004168 | 0,301 | 4,28E-02 |
| B3GNT8 | ENSBTAG00000016041 | ENSBTAG00000016041 | 3,318 | 4,30E-02 |
| TOMM7 | ENSBTAG00000051150 | ENSBTAG00000051150 | -2,937 | 4,31E-02 |
| FSHR | ENSBTAG00000032424 | ENSBTAG00000032424 | 0,436 | 4,32E-02 |
| SGIP1 | ENSBTAG00000011091 | ENSBTAG00000011091 | 2,357 | 4,32E-02 |
| TUBB1 | ENSBTAG00000018785 | ENSBTAG00000018785 | -0,307 | 4,32E-02 |
| TUBB4A | ENSBTAG00000021013 | ENSBTAG00000021013 | -0,251 | 4,32E-02 |
| DMRTA1 | ENSBTAG00000001315 | ENSBTAG00000001315 | 1,574 | 4,33E-02 |
| ZNF423 | ENSBTAG00000017397 | ENSBTAG00000017397 | -0,428 | 4,33E-02 |
| RPL17 | ENSBTAG00000022902 | ENSBTAG00000022902 | 1,999 | 4,34E-02 |
| VASP | ENSBTAG00000019604 | ENSBTAG00000019604 | -0,155 | 4,34E-02 |
| FAM210B | ENSBTAG00000013007 | ENSBTAG00000013007 | 0,366 | 4,35E-02 |
| ERG | ENSBTAG00000011001 | ENSBTAG00000011001 | 1,213 | 4,36E-02 |
| KRIT1 | ENSBTAG00000002750 | ENSBTAG00000002750 | 0,172 | 4,36E-02 |
| POLQ | ENSBTAG00000001920 | ENSBTAG00000001920 | -0,478 | 4,37E-02 |
| MYPOP | ENSBTAG00000045708 | ENSBTAG00000045708 | 0,27 | 4,38E-02 |
| TRA2A | ENSBTAG00000012622 | ENSBTAG00000012622 | -0,208 | 4,38E-02 |
| CDK1 | ENSBTAG00000010109 | ENSBTAG00000010109 | -0,363 | 4,40E-02 |
| RNF185 | ENSBTAG00000002828 | ENSBTAG00000002828 | 0,234 | 4,43E-02 |
| DNAJC22 | ENSBTAG00000016398 | ENSBTAG00000016398 | 0,207 | 4,44E-02 |
| MAGOH | ENSBTAG00000003485 | ENSBTAG00000003485 | -0,168 | 4,45E-02 |
| EOGT | ENSBTAG00000022681 | ENSBTAG00000022681 | 0,257 | 4,46E-02 |
| PSMD6 | ENSBTAG00000015112 | ENSBTAG00000015112 | -0,145 | 4,46E-02 |
| ALDH3A2 | ENSBTAG00000039161 | ENSBTAG00000039161 | 0,38 | 4,47E-02 |
| STK32B | ENSBTAG00000015409 | ENSBTAG00000015409 | -0,502 | 4,47E-02 |
| CEP57L1 | ENSBTAG00000019410 | ENSBTAG00000019410 | -0,224 | 4,48E-02 |
| LYL1 | ENSBTAG00000019329 | ENSBTAG00000019329 | -0,215 | 4,48E-02 |
| CHEK2 | ENSBTAG00000004956 | ENSBTAG00000004956 | -0,379 | 4,49E-02 |
| STAT3 | ENSBTAG00000021523 | ENSBTAG00000021523 | 0,153 | 4,49E-02 |
| LRRC75A | ENSBTAG00000025078 | ENSBTAG00000025078 | -0,556 | 4,51E-02 |
| HHIPL1 | ENSBTAG00000026913 | ENSBTAG00000026913 | 0,333 | 4,54E-02 |
| ORC1 | ENSBTAG00000002719 | ENSBTAG00000002719 | -0,559 | 4,54E-02 |
| INHBA | ENSBTAG00000048508 | ENSBTAG00000048508 | -0,151 | 4,55E-02 |
| DHRS13 | ENSBTAG00000013501 | ENSBTAG00000013501 | -0,315 | 4,56E-02 |
| AKR1C3 | ENSBTAG00000021368 | ENSBTAG00000021368 | 1,148 | 4,57E-02 |
| NEFM | ENSBTAG00000039530 | ENSBTAG00000039530 | -2,25 | 4,59E-02 |
| PSMA6 | ENSBTAG00000009683 | ENSBTAG00000009683 | -0,156 | 4,59E-02 |
| TWIST2 | ENSBTAG00000021444 | ENSBTAG00000021444 | -0,277 | 4,59E-02 |
| GRK5 | ENSBTAG00000007981 | ENSBTAG00000007981 | -0,186 | 4,60E-02 |
| KCNA4 | ENSBTAG00000020793 | ENSBTAG00000020793 | 0,46 | 4,62E-02 |
| FAM83H | ENSBTAG00000038682 | ENSBTAG00000038682 | 0,249 | 4,63E-02 |
| FZD7 | ENSBTAG00000002283 | ENSBTAG00000002283 | -0,185 | 4,63E-02 |
| H2AZ2 | ENSBTAG00000016975 | ENSBTAG00000016975 | -0,185 | 4,63E-02 |
| HEATR5A | ENSBTAG00000022847 | ENSBTAG00000022847 | 0,206 | 4,64E-02 |
| MCM2 | ENSBTAG00000014380 | ENSBTAG00000014380 | -0,412 | 4,64E-02 |
| PLCD3 | ENSBTAG00000006052 | ENSBTAG00000006052 | 0,148 | 4,65E-02 |
| CAMK1D | ENSBTAG00000008650 | ENSBTAG00000008650 | 0,242 | 4,66E-02 |
| RNF128 | ENSBTAG00000022920 | ENSBTAG00000022920 | 0,254 | 4,67E-02 |
| PLA2R1 | ENSBTAG00000032515 | ENSBTAG00000032515 | 0,338 | 4,68E-02 |
| CIT | ENSBTAG00000008963 | ENSBTAG00000008963 | -0,563 | 4,69E-02 |
| LRRC24 | ENSBTAG00000004970 | ENSBTAG00000004970 | 0,268 | 4,69E-02 |
| SLC52A2 | ENSBTAG00000000857 | ENSBTAG00000000857 | 0,265 | 4,69E-02 |
| CCND3 | ENSBTAG00000010106 | ENSBTAG00000010106 | -0,219 | 4,71E-02 |
| HDAC9 | ENSBTAG00000003808 | ENSBTAG00000003808 | 0,23 | 4,71E-02 |
| Fam13a | ENSBTAG00000011187 | ENSBTAG00000011187 | 0,223 | 4,72E-02 |
| PON2 | ENSBTAG00000008361 | ENSBTAG00000008361 | 0,13 | 4,72E-02 |
| FRK | ENSBTAG00000020018 | ENSBTAG00000020018 | 0,394 | 4,73E-02 |
| GLRA2 | ENSBTAG00000017209 | ENSBTAG00000017209 | -1,433 | 4,73E-02 |
| TK1 | ENSBTAG00000007121 | ENSBTAG00000007121 | -0,691 | 4,73E-02 |
| TOB2 | ENSBTAG00000015109 | ENSBTAG00000015109 | 0,18 | 4,73E-02 |
| IMPACT | ENSBTAG00000003035 | ENSBTAG00000003035 | 0,168 | 4,74E-02 |
| NAMPT | ENSBTAG00000015509 | ENSBTAG00000015509 | 0,168 | 4,75E-02 |
| UBE2E2 | ENSBTAG00000047930 | ENSBTAG00000047930 | 0,297 | 4,76E-02 |
| LRRIQ3 | ENSBTAG00000019401 | ENSBTAG00000019401 | 1,041 | 4,78E-02 |
| BCS1L | ENSBTAG00000003813 | ENSBTAG00000003813 | -0,221 | 4,79E-02 |
| CDIN1 | ENSBTAG00000002179 | ENSBTAG00000002179 | -0,193 | 4,80E-02 |
| CCNB2 | ENSBTAG00000005269 | ENSBTAG00000005269 | -0,549 | 4,81E-02 |
| GDF11 | ENSBTAG00000007417 | ENSBTAG00000007417 | -0,426 | 4,81E-02 |
| ACAD10 | ENSBTAG00000001164 | ENSBTAG00000001164 | 0,298 | 4,82E-02 |
| NOL12 | ENSBTAG00000051118 | ENSBTAG00000051118 | -0,397 | 4,83E-02 |
| PDCD7 | ENSBTAG00000039658 | ENSBTAG00000039658 | -0,2 | 4,83E-02 |
| HSPD1 | ENSBTAG00000012586 | ENSBTAG00000012586 | -0,173 | 4,84E-02 |
| FANCB | ENSBTAG00000016433 | ENSBTAG00000016433 | -1,278 | 4,85E-02 |
| ACOT7 | ENSBTAG00000021202 | ENSBTAG00000021202 | -0,25 | 4,87E-02 |
| BORCS5 | ENSBTAG00000008989 | ENSBTAG00000008989 | -0,268 | 4,88E-02 |
| KCTD8 | ENSBTAG00000038659 | ENSBTAG00000038659 | 0,337 | 4,88E-02 |
| SRSF3 | ENSBTAG00000040006 | ENSBTAG00000040006 | -0,213 | 4,88E-02 |
| ATP13A2 | ENSBTAG00000008309 | ENSBTAG00000008309 | 0,194 | 4,90E-02 |
| FKBP4 | ENSBTAG00000007605 | ENSBTAG00000007605 | -0,187 | 4,90E-02 |
| SAT1 | ENSBTAG00000017363 | ENSBTAG00000017363 | 0,204 | 4,92E-02 |
| NCAPG | ENSBTAG00000021582 | ENSBTAG00000021582 | -0,518 | 4,94E-02 |
| MED30 | ENSBTAG00000026254 | ENSBTAG00000026254 | -0,259 | 4,97E-02 |
| RGMB | ENSBTAG00000011864 | ENSBTAG00000011864 | -0,333 | 4,97E-02 |
| GPNMB | ENSBTAG00000000604 | ENSBTAG00000000604 | 0,751 | 4,99E-02 |
| KANK1 | ENSBTAG00000010976 | ENSBTAG00000010976 | 0,22 | 4,99E-02 |
| PRR5L | ENSBTAG00000021029 | ENSBTAG00000021029 | -0,309 | 4,99E-02 |

**Supplemental Table S6: List of DEGs in melatonin + 4P-PDOT treatment (FC=0, adjusted p-value ≤ 0.05)**

| Symbol | Entrez Gene Name | ID | Expr Log Ratio | Expr p-value |
| --- | --- | --- | --- | --- |
| AARS1 | alanyl-tRNA synthetase 1 | ENSBTAG00000019810 | -0,099 | 3,22E-02 |
| APC2 | APC regulator of WNT signaling pathway 2 | ENSBTAG00000015433 | 0,307 | 4,01E-02 |
| ATMIN | ATM interactor | ENSBTAG00000008213 | 0,124 | 4,21E-02 |
| BFSP1 | beaded filament structural protein 1 | ENSBTAG00000021867 | -2,499 | 4,74E-02 |
| C2orf73 | chromosome 2 open reading frame 73 | ENSBTAG00000006861 | 4,084 | 3,80E-02 |
| C7 | complement C7 | ENSBTAG00000011766 | -0,236 | 1,70E-02 |
| CAMK2A | calcium/calmodulin dependent protein kinase II alpha | ENSBTAG00000020087 | -0,634 | 2,77E-02 |
| CASKIN2 | CASK interacting protein 2 | ENSBTAG00000007220 | 0,132 | 2,21E-02 |
| CBX1 | chromobox 1 | ENSBTAG00000038411 | -0,273 | 5,51E-04 |
| CBX5 | chromobox 5 | ENSBTAG00000006246 | -0,358 | 3,90E-03 |
| CCDC136 | coiled-coil domain containing 136 | ENSBTAG00000011002 | -0,460 | 9,31E-04 |
| Celf1 | CUGBP, Elav-like family member 1 | ENSBTAG00000002520 | -0,185 | 3,40E-02 |
| CEP104 | centrosomal protein 104 | ENSBTAG00000020014 | 0,386 | 1,23E-02 |
| CEP85 | centrosomal protein 85 | ENSBTAG00000009579 | -0,193 | 4,87E-02 |
| CFAP126 | cilia and flagella associated protein 126 | ENSBTAG00000015856 | -0,317 | 3,26E-02 |
| CFAP77 | cilia and flagella associated protein 77 | ENSBTAG00000019254 | -3,544 | 3,74E-02 |
| CLDN5 | claudin 5 | ENSBTAG00000022020 | -4,364 | 4,61E-02 |
| CLEC2L | C-type lectin domain family 2 member L | ENSBTAG00000038854 | -1,991 | 2,61E-02 |
| CMTM3 | CKLF like MARVEL transmembrane domain containing 3 | ENSBTAG00000054188 | 0,172 | 1,66E-02 |
| COQ8B | coenzyme Q8B | ENSBTAG00000019462 | 0,206 | 3,30E-02 |
| CWF19L1 | CWF19 like cell cycle control factor 1 | ENSBTAG00000007594 | -0,181 | 4,00E-02 |
| DDX6 | DEAD-box helicase 6 | ENSBTAG00000009067 | -0,250 | 2,42E-02 |
| DLL4 | delta like canonical Notch ligand 4 | ENSBTAG00000010361 | -1,767 | 1,60E-02 |
| DNAJB7 | DnaJ heat shock protein family (Hsp40) member B7 | ENSBTAG00000006205 | -0,145 | 4,14E-02 |
| DYNLRB2 | dynein light chain roadblock-type 2 | ENSBTAG00000017752 | 0,780 | 2,39E-02 |
| EIF2D | eukaryotic translation initiation factor 2D | ENSBTAG00000010432 | 0,136 | 4,64E-02 |
| EIF5B | eukaryotic translation initiation factor 5B | ENSBTAG00000034255 | -0,119 | 2,56E-02 |
| ENPP3 | ectonucleotide pyrophosphatase/phosphodiesterase 3 | ENSBTAG00000020196 | -3,259 | 2,18E-02 |
| ESF1 | ESF1 nucleolar pre-rRNA processing protein homolog | ENSBTAG00000007130 | -0,163 | 4,23E-02 |
| EXOSC2 | exosome component 2 | ENSBTAG00000048071 | -0,258 | 1,99E-02 |
| FAM186B | family with sequence similarity 186 member B | ENSBTAG00000019283 | -1,849 | 3,12E-02 |
| FANCB | FA complementation group B | ENSBTAG00000016433 | -1,356 | 2,93E-02 |
| FBXL20 | F-box and leucine rich repeat protein 20 | ENSBTAG00000008371 | -0,213 | 4,65E-02 |
| FUT11 | fucosyltransferase 11 | ENSBTAG00000002792 | -0,194 | 4,00E-02 |
| GABARAPL1 | GABA type A receptor associated protein like 1 | ENSBTAG00000011765 | 0,149 | 9,11E-03 |
| GJC1 | gap junction protein gamma 1 | ENSBTAG00000004036 | -0,223 | 2,41E-02 |
| GLRA2 | glycine receptor alpha 2 | ENSBTAG00000017209 | -1,600 | 2,66E-02 |
| Gm16500 |  | ENSBTAG00000022109 | -0,186 | 4,04E-02 |
| GMFG | glia maturation factor gamma | ENSBTAG00000005390 | -0,986 | 2,21E-02 |
| GPR88 | G protein-coupled receptor 88 | ENSBTAG00000021101 | 4,110 | 2,47E-02 |
| GRM8 | glutamate metabotropic receptor 8 | ENSBTAG00000054974 | 2,175 | 4,56E-02 |
| GRPEL2 | GrpE like 2, mitochondrial | ENSBTAG00000019950 | -0,510 | 1,13E-02 |
| GUCA1A | guanylate cyclase activator 1A | ENSBTAG00000012822 | -1,972 | 2,63E-02 |
| GYG1 | glycogenin 1 | ENSBTAG00000001721 | 0,141 | 4,73E-02 |
| HAP1 | huntingtin associated protein 1 | ENSBTAG00000052414 | -1,608 | 4,28E-02 |
| HAS2 | hyaluronan synthase 2 | ENSBTAG00000019892 | -0,221 | 4,44E-02 |
| HOXB13 | homeobox B13 | ENSBTAG00000014663 | -1,615 | 4,56E-02 |
| INHBA | inhibin subunit beta A | ENSBTAG00000048508 | -0,229 | 2,04E-03 |
| ISM1 | isthmin 1 | ENSBTAG00000017188 | -0,552 | 3,86E-02 |
| KCNJ11 | potassium inwardly rectifying channel subfamily J member 11 | ENSBTAG00000016170 | -3,930 | 3,64E-02 |
| KCNT1 | potassium sodium-activated channel subfamily T member 1 | ENSBTAG00000018975 | -2,390 | 1,43E-02 |
| KDSR | 3-ketodihydrosphingosine reductase | ENSBTAG00000007723 | 0,274 | 2,93E-02 |
| KLHL11 | kelch like family member 11 | ENSBTAG00000050549 | -0,412 | 2,86E-03 |
| KRR1 | KRR1 small subunit processome component homolog | ENSBTAG00000011591 | -0,161 | 1,72E-02 |
| KRTCAP3 | keratinocyte associated protein 3 | ENSBTAG00000018156 | 1,804 | 8,82E-03 |
| LPGAT1 | lysophosphatidylglycerol acyltransferase 1 | ENSBTAG00000005137 | -0,237 | 2,67E-02 |
| MAP3K13 | mitogen-activated protein kinase kinase kinase 13 | ENSBTAG00000017103 | -0,335 | 4,08E-02 |
| MARVELD2 | MARVEL domain containing 2 | ENSBTAG00000040001 | -0,356 | 4,86E-02 |
| MCF2 | MCF.2 cell line derived transforming sequence | ENSBTAG00000004007 | -3,973 | 1,87E-02 |
| MDM4 | MDM4 regulator of p53 | ENSBTAG00000006255 | -0,391 | 2,12E-03 |
| MED12L | mediator complex subunit 12L | ENSBTAG00000021703 | 0,668 | 2,16E-02 |
| MOB3A | MOB kinase activator 3A | ENSBTAG00000014526 | -0,184 | 2,65E-02 |
| MSRB2 | methionine sulfoxide reductase B2 | ENSBTAG00000044112 | 0,191 | 4,87E-02 |
| MTDH | metadherin | ENSBTAG00000003098 | -0,106 | 3,85E-02 |
| MTM1 | myotubularin 1 | ENSBTAG00000014138 | 0,297 | 9,69E-03 |
| MYH15 | myosin heavy chain 15 | ENSBTAG00000018399 | -0,627 | 4,81E-02 |
| MYPOP | Myb related transcription factor, partner of profilin | ENSBTAG00000045708 | 0,391 | 2,66E-03 |
| NAA50 | N-alpha-acetyltransferase 50, NatE catalytic subunit | ENSBTAG00000007784 | -0,174 | 5,44E-03 |
| NEGR1 | neuronal growth regulator 1 | ENSBTAG00000055278 | -0,252 | 3,49E-02 |
| NEXN | nexilin F-actin binding protein | ENSBTAG00000008921 | -0,151 | 2,68E-02 |
| NF2 | neurofibromin 2 | ENSBTAG00000013153 | -0,241 | 3,45E-02 |
| NFE2 | nuclear factor, erythroid 2 | ENSBTAG00000001562 | -1,530 | 1,28E-02 |
| NFIA | nuclear factor I A | ENSBTAG00000000074 | -0,332 | 2,33E-02 |
| NOL9 | nucleolar protein 9 | ENSBTAG00000016733 | -0,263 | 3,10E-02 |
| NUCKS1 | nuclear casein kinase and cyclin dependent kinase substrate 1 | ENSBTAG00000008001 | -0,223 | 4,06E-02 |
| NUDT3 | nudix hydrolase 3 | ENSBTAG00000019684 | -0,178 | 2,32E-02 |
| OPRL1 | opioid related nociceptin receptor 1 | ENSBTAG00000049473 | 0,639 | 3,42E-02 |
| ORAI1 | ORAI calcium release-activated calcium modulator 1 | ENSBTAG00000004457 | 0,186 | 2,09E-02 |
| OTOP1 | otopetrin 1 | ENSBTAG00000003651 | -3,206 | 1,46E-02 |
| OTUD3 | OTU deubiquitinase 3 | ENSBTAG00000017108 | -0,386 | 1,24E-02 |
| PALS2 | protein associated with LIN7 2, MAGUK family member | ENSBTAG00000015303 | -0,156 | 2,86E-02 |
| PPFIBP1 | PPFIA binding protein 1 | ENSBTAG00000004283 | -0,165 | 3,26E-02 |
| PPIG | peptidylprolyl isomerase G | ENSBTAG00000027789 | -0,121 | 4,32E-02 |
| PSMC1 | proteasome 26S subunit, ATPase 1 | ENSBTAG00000005426 | -0,148 | 1,15E-02 |
| PTGFR | prostaglandin F receptor | ENSBTAG00000015902 | 0,271 | 3,63E-02 |
| RASSF3 | Ras association domain family member 3 | ENSBTAG00000051013 | -0,132 | 3,43E-02 |
| RETSAT | retinol saturase | ENSBTAG00000021934 | 0,132 | 3,97E-02 |
| RFFL | ring finger and FYVE like domain containing E3 ubiquitin protein ligase | ENSBTAG00000013645 | 0,291 | 3,16E-02 |
| RGS14 | regulator of G protein signaling 14 | ENSBTAG00000008497 | 0,250 | 3,18E-02 |
| RHPN2 | rhophilin Rho GTPase binding protein 2 | ENSBTAG00000003089 | 0,309 | 4,60E-02 |
| RPRD1A | regulation of nuclear pre-mRNA domain containing 1A | ENSBTAG00000005497 | -0,106 | 4,45E-02 |
| RPS13 | ribosomal protein S13 | ENSBTAG00000040308 | -2,642 | 2,89E-02 |
| RXFP3 | relaxin family peptide receptor 3 | ENSBTAG00000039929 | -2,412 | 2,80E-02 |
| S100A5 | S100 calcium binding protein A5 | ENSBTAG00000000644 | -3,014 | 3,35E-02 |
| SLC16A4 | solute carrier family 16 member 4 | ENSBTAG00000015380 | 0,171 | 4,10E-02 |
| SMS | spermine synthase | ENSBTAG00000018382 | -0,151 | 2,85E-02 |
| SPATA5 | spermatogenesis associated 5 | ENSBTAG00000000125 | -0,522 | 7,54E-03 |
| TFPI | tissue factor pathway inhibitor | ENSBTAG00000049919 | -0,394 | 2,54E-02 |
| TGFBR1 | transforming growth factor beta receptor 1 | ENSBTAG00000018035 | -0,202 | 1,79E-02 |
| TGS1 | trimethylguanosine synthase 1 | ENSBTAG00000005898 | -0,274 | 4,26E-02 |
| TIGAR | TP53 induced glycolysis regulatory phosphatase | ENSBTAG00000016650 | -0,270 | 4,18E-02 |
| TMEM14A | transmembrane protein 14A | ENSBTAG00000005206 | -0,203 | 3,34E-02 |
| TMEM241 | transmembrane protein 241 | ENSBTAG00000010037 | -0,429 | 1,93E-02 |
| TMEM41B | transmembrane protein 41B | ENSBTAG00000001607 | -0,275 | 4,05E-02 |
| TNFRSF8 | TNF receptor superfamily member 8 | ENSBTAG00000039937 | -3,943 | 3,54E-02 |
| TNRC6A | trinucleotide repeat containing adaptor 6A | ENSBTAG00000017999 | -0,260 | 5,04E-03 |
| TRMT44 | tRNA methyltransferase 44 homolog | ENSBTAG00000004797 | -0,453 | 3,73E-02 |
| TTC9 | tetratricopeptide repeat domain 9 | ENSBTAG00000045604 | -1,493 | 2,98E-03 |
| UAP1L1 | UDP-N-acetylglucosamine pyrophosphorylase 1 like 1 | ENSBTAG00000048054 | -0,249 | 4,58E-02 |
| UBQLN2 | ubiquilin 2 | ENSBTAG00000021843 | 0,149 | 8,84E-03 |
| VPS36 | vacuolar protein sorting 36 homolog | ENSBTAG00000004307 | 0,145 | 4,20E-02 |
| ZDHHC14 | zinc finger DHHC-type palmitoyltransferase 14 | ENSBTAG00000018464 | 0,182 | 4,17E-02 |
| ZNF319 | zinc finger protein 319 | ENSBTAG00000045572 | 0,209 | 2,36E-02 |
| ZNF583 | zinc finger protein 583 | ENSBTAG00000000801 | 0,429 | 3,64E-02 |
| ZNF629 | zinc finger protein 629 | ENSBTAG00000026307 | 0,179 | 1,91E-02 |

**Supplemental Table S7: List of upstream regulators in treatments (melatonin (10^-7^ M), melatonin (10^-9^ M), melatonin+ N-acetyl serotonin, melatonin+ S26131, melatonin+IIK7, melatonin+ 4P-PDOT) (p-value of overlap ≥ 0,05)**

| Melatonin treatment (10^-7^ M) | | | | | |
| --- | --- | --- | --- | --- | --- |
| Upstream Regulator | **Expr Log Ratio** | **Molecule Type** | **Predicted Activation State** | **Activation z-score** | **p-value of overlap** |
| HDAC4 | 0,416 | transcription regulator |  |  | 2,08E-03 |
| GJB2 |  | transporter |  |  | 3,78E-03 |
| Ikzf1 |  | transcription regulator |  |  | 3,78E-03 |
| EWSR1 |  | other |  |  | 7,33E-03 |
| ATG12 | -0,001 | other |  |  | 7,55E-03 |
| SNF8 | -0,053 | enzyme |  |  | 7,55E-03 |
| CCHCR1 | -0,048 | other |  |  | 7,55E-03 |
| DBF4B | -0,295 | other |  |  | 7,55E-03 |
| MSH3 | 0,014 | enzyme |  |  | 7,55E-03 |
| furegrelate |  | chemical reagent |  |  | 7,55E-03 |
| INHA | 0,884 | growth factor |  |  | 7,61E-03 |
| SOX10 | -0,037 | transcription regulator |  |  | 8,19E-03 |
| pregnenolone sulfate |  | chemical - endogenous mammalian |  |  | 1,13E-02 |
| sethoxydim |  | chemical toxicant |  |  | 1,13E-02 |
| UBA6 | -0,022 | enzyme |  |  | 1,13E-02 |
| RAD51 | -0,091 | enzyme |  |  | 1,13E-02 |
| CLCN1 |  | ion channel |  |  | 1,13E-02 |
| GABA-A receptor |  | complex |  |  | 1,13E-02 |
| MED13 | 0,102 | transcription regulator |  |  | 1,37E-02 |
| levodopa |  | chemical - endogenous mammalian |  | 1,134 | 1,40E-02 |
| HMG20B |  | transcription regulator |  |  | 1,50E-02 |
| DTNB | 0,038 | other |  |  | 1,50E-02 |
| LAMB1 | 0,028 | other |  |  | 1,50E-02 |
| SEMA3E | -0,083 | other |  |  | 1,50E-02 |
| DDX19B | -0,156 | enzyme |  |  | 1,50E-02 |
| GZMB |  | peptidase |  |  | 1,50E-02 |
| Pou3f1 |  | transcription regulator |  |  | 1,50E-02 |
| NTRK3 | 0,072 | kinase |  |  | 1,50E-02 |
| BI 2536 |  | chemical drug |  |  | 1,88E-02 |
| NPAS4 | -2,296 | transcription regulator |  |  | 1,88E-02 |
| FOXN4 |  | transcription regulator |  |  | 1,88E-02 |
| PFDN6 | -0,183 | other |  |  | 1,88E-02 |
| Laminin2 |  | complex |  |  | 1,88E-02 |
| CYP46A1 | 0,424 | enzyme |  |  | 1,88E-02 |
| KCND2 | 0,212 | ion channel |  |  | 1,88E-02 |
| VBP1 | 0,000 | other |  |  | 1,88E-02 |
| PFDN2 | -0,120 | other |  |  | 1,88E-02 |
| AGA | -0,026 | enzyme |  |  | 1,88E-02 |
| SQ 29548 |  | chemical reagent |  |  | 1,88E-02 |
| NFYB | -0,022 | transcription regulator |  |  | 1,95E-02 |
| pubchem compound 135889696 |  | chemical reagent |  |  | 2,25E-02 |
| tacedinaline |  | chemical drug |  |  | 2,25E-02 |
| T-2 toxin |  | chemical toxicant |  |  | 2,25E-02 |
| PFDN5 | -0,038 | transcription regulator |  |  | 2,25E-02 |
| OLIG2 |  | transcription regulator |  |  | 2,25E-02 |
| miR-9-3p (and other miRNAs w/seed UAAAGCU) |  | mature microRNA |  |  | 2,25E-02 |
| PHGDH | -0,061 | enzyme |  |  | 2,25E-02 |
| tributyrin |  | chemical drug |  |  | 2,52E-02 |
| AZD4547 |  | chemical drug |  |  | 2,62E-02 |
| miR-494-3p (miRNAs w/seed GAAACAU) |  | mature microRNA |  |  | 2,62E-02 |
| PIK3C3 | 0,060 | kinase |  |  | 2,62E-02 |
| MED13L | 0,043 | other |  |  | 2,62E-02 |
| ERBIN | -0,000 | other |  |  | 2,62E-02 |
| NKX3-2 | 0,236 | transcription regulator |  |  | 2,62E-02 |
| ITGB1BP1 | -0,021 | other |  |  | 2,62E-02 |
| trimethyltin |  | chemical reagent |  |  | 2,62E-02 |
| SOX7 | -0,204 | transcription regulator |  |  | 2,67E-02 |
| Collagen type I (complex) |  | complex |  |  | 2,82E-02 |
| CX3CR1 | 1,741 | G-protein coupled receptor |  |  | 2,88E-02 |
| cosyntropin |  | biologic drug |  |  | 2,99E-02 |
| miR-140-5p (and other miRNAs w/seed AGUGGUU) |  | mature microRNA |  |  | 2,99E-02 |
| SOX18 | 0,287 | transcription regulator |  |  | 2,99E-02 |
| LAMA4 | 0,022 | enzyme |  |  | 2,99E-02 |
| DNM3OS |  | other |  |  | 3,35E-02 |
| GUSB | 0,032 | enzyme |  |  | 3,35E-02 |
| MI-503 |  | chemical reagent |  |  | 3,35E-02 |
| ROR2 | -0,046 | kinase |  |  | 3,53E-02 |
| KLF4 | 0,259 | transcription regulator |  | -0,320 | 3,55E-02 |
| USP37 | 0,075 | peptidase |  |  | 3,72E-02 |
| SIK1/SIK1B |  | kinase |  |  | 3,72E-02 |
| INCB054329 |  | chemical drug |  |  | 3,72E-02 |
| ZFP57 | -0,097 | transcription regulator |  |  | 4,08E-02 |
| DLG1 | -0,003 | kinase |  |  | 4,08E-02 |
| quizartinib |  | chemical drug |  |  | 4,08E-02 |
| miR-214-3p (and other miRNAs w/seed CAGCAGG) |  | mature microRNA |  |  | 4,08E-02 |
| VLDLR | 0,142 | transporter |  |  | 4,08E-02 |
| CNB-001 |  | chemical reagent |  |  | 4,08E-02 |
| 7-(4-fluorobenzylamino)-1,3,4,8-tetrahydropyrrolo[4,3,2-de]quinolin-8(1H)-one |  | chemical reagent |  |  | 4,08E-02 |
| SOX2 |  | transcription regulator |  | 0,067 | 4,23E-02 |
| NUMBL | 0,056 | other |  |  | 4,45E-02 |
| KRIT1 | 0,031 | other |  |  | 4,45E-02 |
| HOXC4 | 0,030 | transcription regulator |  |  | 4,45E-02 |
| pentylenetetrazol |  | chemical drug |  |  | 4,45E-02 |
| NEDD9 | 0,394 | other |  |  | 4,48E-02 |
| miR-16-5p (and other miRNAs w/seed AGCAGCA) |  | mature microRNA |  |  | 4,76E-02 |
| Laminin1 |  | complex |  |  | 4,81E-02 |
| SOX5 | 0,059 | transcription regulator |  |  | 4,81E-02 |
| USP8 | -0,025 | peptidase |  |  | 4,81E-02 |
| ETV6 | 0,031 | transcription regulator |  |  | 4,81E-02 |
| astressin 2B |  | biologic drug |  |  | 4,81E-02 |

| Melatonin treatment (10^-9^ M) | | | | | |
| --- | --- | --- | --- | --- | --- |
| Upstream Regulator | **Expr Log Ratio** | **Molecule Type** | **Predicted Activation State** | **Activation z-score** | **p-value of overlap** |
| LDL-cholesterol |  | complex |  |  | 3,08E-04 |
| EPZ005687 |  | chemical - protease inhibitor |  |  | 3,08E-04 |
| SOX18 | 1,064 | transcription regulator |  |  | 5,72E-04 |
| HSPG2 | 0,105 | enzyme |  |  | 5,98E-04 |
| NSD2 | -0,056 | enzyme |  |  | 7,58E-04 |
| NQO2 | -1,203 | enzyme |  |  | 9,14E-04 |
| sildenafil |  | chemical drug |  |  | 9,42E-04 |
| highly active antiretroviral therapy |  | chemical drug |  |  | 1,33E-03 |
| AFAP1-AS1 |  | other |  |  | 1,33E-03 |
| KRIT1 | -0,014 | other |  |  | 1,33E-03 |
| phencyclidine |  | chemical drug |  |  | 1,57E-03 |
| IFNL2 |  | other |  |  | 2,10E-03 |
| VEGFC | 0,054 | growth factor |  |  | 2,10E-03 |
| PLD2 | -0,010 | enzyme |  |  | 2,70E-03 |
| miR-486-5p (and other miRNAs w/seed CCUGUAC) |  | mature microRNA |  |  | 3,38E-03 |
| mir-486 |  | microRNA |  |  | 3,74E-03 |
| GF 120918 |  | chemical reagent |  |  | 4,13E-03 |
| Abcb1b |  | transporter |  |  | 4,52E-03 |
| probucol |  | chemical drug |  |  | 4,52E-03 |
| hydroxamic acid |  | chemical - other |  |  | 4,58E-03 |
| HAS2-AS1 |  | other |  |  | 4,58E-03 |
| sodium thiosulfate |  | chemical drug |  |  | 4,58E-03 |
| MPHOSPH8 | 0,067 | transcription regulator |  |  | 4,58E-03 |
| OXSM | 0,720 | kinase |  |  | 4,58E-03 |
| LOC299282 |  | other |  |  | 4,58E-03 |
| RGN | 0,352 | enzyme |  |  | 4,58E-03 |
| INTS12 | -0,064 | other |  |  | 4,58E-03 |
| CCDC71L |  | other |  |  | 4,58E-03 |
| THAP11 | 0,000 | transcription regulator |  |  | 4,58E-03 |
| CLEC16A | 0,027 | other |  |  | 4,58E-03 |
| UBE3D | 0,054 | enzyme |  |  | 4,58E-03 |
| ZG16B |  | other |  |  | 4,58E-03 |
| METAP2 | -0,045 | peptidase |  |  | 4,58E-03 |
| SMCHD1 | 0,014 | enzyme |  |  | 4,58E-03 |
| voxtalisib |  | chemical drug |  |  | 4,58E-03 |
| pidilizumab |  | biologic drug |  |  | 4,58E-03 |
| NAPA | 0,032 | transporter |  |  | 4,58E-03 |
| MRGBP | -0,081 | other |  |  | 4,58E-03 |
| SINHCAF | -0,017 | other |  |  | 4,58E-03 |
| CCZ1/CCZ1B |  | other |  |  | 4,58E-03 |
| mir-592 |  | microRNA |  |  | 4,58E-03 |
| NNMT |  | enzyme |  |  | 4,58E-03 |
| DYNLT3 | 0,054 | other |  |  | 4,58E-03 |
| CBLL1 | -0,088 | enzyme |  |  | 4,58E-03 |
| DHX29 | -0,009 | enzyme |  |  | 4,58E-03 |
| GNG14 |  | other |  |  | 4,58E-03 |
| CAB39 | -0,085 | enzyme |  |  | 4,58E-03 |
| INTS6 | 0,017 | enzyme |  |  | 4,58E-03 |
| NCBP2 | -0,005 | other |  |  | 4,58E-03 |
| CEP350 | 0,032 | other |  |  | 4,58E-03 |
| RIT1 | -0,008 | enzyme |  |  | 4,58E-03 |
| PDS5A | -0,014 | other |  |  | 4,58E-03 |
| CD164 | 0,028 | other |  |  | 4,58E-03 |
| tetramethylpyrazine |  | chemical - endogenous non-mammalian |  |  | 4,58E-03 |
| TPD52 | -0,005 | other |  |  | 4,58E-03 |
| PACRG |  | other |  |  | 4,58E-03 |
| UBAP2L | -0,013 | other |  |  | 4,58E-03 |
| MEAF6 | 0,056 | other |  |  | 4,58E-03 |
| melarsoprol |  | chemical drug |  |  | 4,58E-03 |
| pegademase bovine |  | biologic drug |  |  | 4,58E-03 |
| oxaprozin |  | chemical drug |  |  | 4,58E-03 |
| AuNP@PEG@e14a2 |  | chemical reagent |  |  | 4,58E-03 |
| STAT3 inhibitor IX |  | chemical reagent |  |  | 4,58E-03 |
| polysialic acid |  | chemical drug |  |  | 4,58E-03 |
| stanozolol |  | chemical drug |  |  | 4,58E-03 |
| 6alpha-fluorotestosterone |  | chemical toxicant |  |  | 4,58E-03 |
| MAP3K3 | 0,017 | kinase |  |  | 5,37E-03 |
| Cdkn1c | 0,249 | other |  |  | 5,37E-03 |
| LINC00963 |  | other |  |  | 5,82E-03 |
| PTGER4 | 0,386 | G-protein coupled receptor |  | 0,000 | 5,93E-03 |
| LEF1 | -0,057 | transcription regulator |  | -0,832 | 6,47E-03 |
| PPP3CA | 0,064 | phosphatase |  |  | 6,59E-03 |
| risperidone |  | chemical drug |  |  | 6,77E-03 |
| HDAC9 | 0,107 | transcription regulator |  |  | 6,77E-03 |
| amphetamine |  | chemical drug |  |  | 7,26E-03 |
| AREG | -0,023 | growth factor |  |  | 7,74E-03 |
| Smad2/3-Smad4 |  | complex |  |  | 7,79E-03 |
| letrozole |  | chemical drug |  |  | 7,79E-03 |
| buthionine sulfoximine |  | chemical drug |  |  | 7,79E-03 |
| NOTCH1 | 0,071 | transcription regulator |  | -0,659 | 8,00E-03 |
| NR3C1 | -0,001 | ligand-dependent nuclear receptor |  |  | 8,06E-03 |
| CPE | -0,022 | peptidase |  |  | 8,32E-03 |
| fucoidin |  | chemical reagent |  |  | 8,32E-03 |
| ACKR3 | -0,008 | G-protein coupled receptor |  |  | 8,87E-03 |
| aspirin |  | chemical drug |  | -1,165 | 8,98E-03 |
| amikacin |  | chemical drug |  |  | 9,14E-03 |
| AEOL-10150 |  | chemical drug |  |  | 9,14E-03 |
| thymol |  | chemical reagent |  |  | 9,14E-03 |
| 4930426D05Rik |  | other |  |  | 9,14E-03 |
| FBXL12 | 0,111 | other |  |  | 9,14E-03 |
| PCDH9 | -0,145 | other |  |  | 9,14E-03 |
| AGO3 | -0,081 | translation regulator |  |  | 9,14E-03 |
| BCL6 peptide inhibitor |  | chemical reagent |  |  | 9,14E-03 |
| bucillamine |  | chemical drug |  |  | 9,14E-03 |
| CACYBP | -0,059 | other |  |  | 9,14E-03 |
| RNMT | 0,035 | enzyme |  |  | 9,14E-03 |
| safflor yellow B |  | chemical - endogenous non-mammalian |  |  | 9,14E-03 |
| NOSIP | 0,044 | other |  |  | 9,14E-03 |
| SYCP3 | -0,737 | other |  |  | 9,14E-03 |
| mir-663 |  | microRNA |  |  | 9,14E-03 |
| TAF5L | -0,005 | transcription regulator |  |  | 9,14E-03 |
| PIM |  | group |  |  | 9,14E-03 |
| UBE4B | 0,057 | enzyme |  |  | 9,14E-03 |
| PLIN3 | 0,057 | other |  |  | 9,14E-03 |
| cyclo(iso-Asp-GR)-LLIIKLAKLAKKLAKLAK |  | chemical reagent |  |  | 9,14E-03 |
| THAP12 | 0,018 | other |  |  | 9,14E-03 |
| NTSR2 | -0,244 | G-protein coupled receptor |  |  | 9,14E-03 |
| AGO4 | -0,048 | translation regulator |  |  | 9,14E-03 |
| STAMBP | 0,019 | enzyme |  |  | 9,14E-03 |
| NEU2 | -1,093 | enzyme |  |  | 9,14E-03 |
| UBE2V1 | -0,061 | transcription regulator |  |  | 9,14E-03 |
| Gm4836 (includes others) |  | other |  |  | 9,14E-03 |
| CLEC2D |  | transmembrane receptor |  |  | 9,14E-03 |
| OTUD5 | -0,004 | enzyme |  |  | 9,14E-03 |
| VNN1 | 0,122 | enzyme |  |  | 9,14E-03 |
| 1,5-bis-(dihexyl-N-nitrosoamino)-2,4-dinitrobenzene |  | chemical reagent |  |  | 9,14E-03 |
| 3830403N18Rik/Xlr |  | other |  |  | 9,14E-03 |
| S7 |  | chemical drug |  |  | 9,14E-03 |
| SI163 |  | chemical drug |  |  | 9,14E-03 |
| S29 |  | chemical drug |  |  | 9,14E-03 |
| daphnoretin |  | chemical - endogenous non-mammalian |  |  | 9,14E-03 |
| PSB-1115 |  | chemical reagent |  |  | 9,14E-03 |
| XK469 |  | chemical drug |  |  | 9,14E-03 |
| talipexole |  | chemical drug |  |  | 9,14E-03 |
| hexaarginine-neomycin B conjugate |  | chemical reagent |  |  | 9,14E-03 |
| AN-207 |  | chemical toxicant |  |  | 9,14E-03 |
| KR 62436 |  | chemical reagent |  |  | 9,14E-03 |
| SSR180575 |  | chemical drug |  |  | 9,14E-03 |
| NSC 651016 |  | chemical reagent |  |  | 9,14E-03 |
| fenoprofen |  | chemical drug |  |  | 9,14E-03 |
| calcium-EDTA |  | chemical drug |  |  | 9,14E-03 |
| isoalantolactone |  | chemical - endogenous non-mammalian |  |  | 9,14E-03 |
| swainsonine |  | chemical - endogenous non-mammalian |  |  | 9,14E-03 |
| desflurane |  | chemical drug |  |  | 9,14E-03 |
| arylsulfonamide 64B |  | chemical reagent |  |  | 9,14E-03 |
| ITGAV | 0,037 | transmembrane receptor |  |  | 9,43E-03 |
| daidzein |  | chemical drug |  |  | 9,81E-03 |
| hymecromone |  | chemical drug |  |  | 1,00E-02 |
| NCOR1 | -0,014 | transcription regulator |  |  | 1,07E-02 |
| acetaminophen |  | chemical drug |  |  | 1,12E-02 |
| NQO1 | -0,099 | enzyme |  |  | 1,31E-02 |
| OGT | -0,096 | enzyme |  |  | 1,31E-02 |
| L-tyrosine |  | chemical - endogenous mammalian |  |  | 1,37E-02 |
| mycosporine-like amino acid |  | chemical reagent |  |  | 1,37E-02 |
| Ggtase I |  | complex |  |  | 1,37E-02 |
| WDR82 | 0,003 | enzyme |  |  | 1,37E-02 |
| G protein beta gamma |  | complex |  |  | 1,37E-02 |
| sethoxydim |  | chemical toxicant |  |  | 1,37E-02 |
| hexa-D-arginine |  | biologic drug |  |  | 1,37E-02 |
| ATXN7L3 | 0,003 | transcription regulator |  |  | 1,37E-02 |
| hyperoside |  | chemical - endogenous non-mammalian |  |  | 1,37E-02 |
| LAPTM4B | 0,038 | other |  |  | 1,37E-02 |
| Integrin alpha 4 beta 1 |  | complex |  |  | 1,37E-02 |
| CKLF |  | cytokine |  |  | 1,37E-02 |
| xenon |  | chemical drug |  |  | 1,37E-02 |
| GEM231 |  | chemical drug |  |  | 1,37E-02 |
| cerebrolysin |  | chemical drug |  |  | 1,37E-02 |
| PLEC | 0,062 | other |  |  | 1,37E-02 |
| 9-benzyl-8-hydroxy-2-(2-methoxyethoxy)adenine |  | chemical reagent |  |  | 1,37E-02 |
| miR-136-5p (miRNAs w/seed CUCCAUU) |  | mature microRNA |  |  | 1,37E-02 |
| miR-153-3p (miRNAs w/seed UGCAUAG) |  | mature microRNA |  |  | 1,37E-02 |
| mir-153 |  | microRNA |  |  | 1,37E-02 |
| HIF1A-AS1 |  | other |  |  | 1,37E-02 |
| NPTX1 | 0,050 | other |  |  | 1,37E-02 |
| KCNQ2 |  | ion channel |  |  | 1,37E-02 |
| STAG2 | 0,014 | other |  |  | 1,37E-02 |
| CCNL2 | 0,050 | other |  |  | 1,37E-02 |
| MAP1S | 0,076 | enzyme |  |  | 1,37E-02 |
| AP2A2 | 0,120 | transporter |  |  | 1,37E-02 |
| RAB3B | 0,014 | enzyme |  |  | 1,37E-02 |
| TRIP12 | 0,010 | enzyme |  |  | 1,37E-02 |
| MT3 |  | other |  |  | 1,37E-02 |
| nilutamide |  | chemical drug |  |  | 1,37E-02 |
| cilomilast |  | chemical drug |  |  | 1,37E-02 |
| tarenflurbil |  | chemical drug |  |  | 1,37E-02 |
| minodronate |  | chemical drug |  |  | 1,37E-02 |
| pramipexole |  | chemical drug |  |  | 1,37E-02 |
| nitromifene |  | chemical reagent |  |  | 1,37E-02 |
| lead nitrate |  | chemical toxicant |  |  | 1,37E-02 |
| imisopasem manganese |  | chemical drug |  |  | 1,37E-02 |
| 10-hydroxycamptothecin |  | chemical - endogenous mammalian |  |  | 1,37E-02 |
| caffeic acid phenethyl ester |  | chemical drug |  |  | 1,38E-02 |
| histone deacetylase |  | complex |  |  | 1,45E-02 |
| pyrrolidine dithiocarbamate |  | chemical reagent |  |  | 1,49E-02 |
| PGF |  | growth factor |  |  | 1,52E-02 |
| ADORA2B | -0,266 | G-protein coupled receptor |  |  | 1,66E-02 |
| mir-150 |  | microRNA |  |  | 1,74E-02 |
| thymoquinone |  | chemical drug |  |  | 1,81E-02 |
| ANGPT1 | -0,045 | growth factor |  |  | 1,81E-02 |
| HBEGF | 0,105 | growth factor |  |  | 1,81E-02 |
| FLT3 | -0,135 | kinase |  |  | 1,81E-02 |
| NME1 | 0,280 | kinase |  |  | 1,81E-02 |
| MICU1 | 0,008 | other |  |  | 1,82E-02 |
| OSGIN1 | 0,164 | growth factor |  |  | 1,82E-02 |
| DAND5 |  | other |  |  | 1,82E-02 |
| OBSCN |  | kinase |  |  | 1,82E-02 |
| AMOTL2 | 0,045 | other |  |  | 1,82E-02 |
| HSPBP1 | 0,027 | other |  |  | 1,82E-02 |
| WAPL | -0,041 | other |  |  | 1,82E-02 |
| enecadin |  | chemical drug |  |  | 1,82E-02 |
| MITF-p300/CBP |  | complex |  |  | 1,82E-02 |
| firtecan pegol |  | chemical drug |  |  | 1,82E-02 |
| ARTN | 0,557 | growth factor |  |  | 1,82E-02 |
| MS4A2 | -0,487 | transmembrane receptor |  |  | 1,82E-02 |
| LAMB1 | 0,030 | other |  |  | 1,82E-02 |
| HAS3 | 0,062 | enzyme |  |  | 1,82E-02 |
| HSPB6 | 0,300 | other |  |  | 1,82E-02 |
| SEMA3E | 0,004 | other |  |  | 1,82E-02 |
| PCGF1 | -0,059 | other |  |  | 1,82E-02 |
| SIGMAR1 | 0,076 | transmembrane receptor |  |  | 1,82E-02 |
| FSCN1 | 0,061 | other |  |  | 1,82E-02 |
| CELF2 |  | other |  |  | 1,82E-02 |
| MED24 | 0,013 | transcription regulator |  |  | 1,82E-02 |
| MME | -0,073 | peptidase |  |  | 1,82E-02 |
| LGALS2 |  | other |  |  | 1,82E-02 |
| TSN | -0,029 | other |  |  | 1,82E-02 |
| DIDO1 | 0,166 | other |  |  | 1,82E-02 |
| WTAP | -0,039 | other |  |  | 1,82E-02 |
| abrocitinib |  | chemical drug |  |  | 1,82E-02 |
| BO-653 |  | chemical drug |  |  | 1,82E-02 |
| TAS-103 |  | chemical drug |  |  | 1,82E-02 |
| pyridoxal phosphate |  | chemical - endogenous mammalian |  |  | 1,82E-02 |
| bumetanide |  | chemical drug |  |  | 1,82E-02 |
| chlorophyll a |  | chemical - endogenous non-mammalian |  |  | 1,82E-02 |
| CCI-007 |  | chemical reagent |  |  | 1,82E-02 |
| galanthamine |  | chemical drug |  |  | 1,82E-02 |
| cyclohexanecarboxylic acid |  | chemical reagent |  |  | 1,82E-02 |
| CDP-choline |  | chemical - endogenous mammalian |  |  | 1,82E-02 |
| steroid hormone |  | chemical - other |  |  | 1,82E-02 |
| interferon beta-1a |  | biologic drug |  |  | 1,94E-02 |
| KDR | 0,178 | kinase |  |  | 1,97E-02 |
| zoledronic acid |  | chemical drug |  |  | 1,97E-02 |
| ZFTA-RELA |  | fusion gene/product |  |  | 1,97E-02 |
| BDNF | -0,040 | growth factor |  | 0,266 | 2,00E-02 |
| ID2 | -0,083 | transcription regulator |  | 0,000 | 2,03E-02 |
| Hsp27 |  | group |  |  | 2,05E-02 |
| DLL4 | -2,064 | other |  |  | 2,05E-02 |
| herbimycin |  | chemical drug |  |  | 2,05E-02 |
| quinolinic acid |  | chemical - endogenous mammalian |  |  | 2,05E-02 |
| reactive oxygen species |  | chemical toxicant |  |  | 2,11E-02 |
| 5-fluorouracil |  | chemical drug |  | -0,422 | 2,23E-02 |
| ILX-23-7553 |  | chemical drug |  |  | 2,27E-02 |
| fludrocortisone |  | chemical drug |  |  | 2,27E-02 |
| Alp |  | group |  |  | 2,27E-02 |
| Ctnna |  | group |  |  | 2,27E-02 |
| (-)-gossypol |  | chemical drug |  |  | 2,27E-02 |
| IFN alpha receptor |  | complex |  |  | 2,27E-02 |
| FOXN4 |  | transcription regulator |  |  | 2,27E-02 |
| LINC00337 |  | other |  |  | 2,27E-02 |
| UTP18 | 0,065 | other |  |  | 2,27E-02 |
| FLVCR1 | -0,073 | transporter |  |  | 2,27E-02 |
| C1GALT1 | 0,050 | enzyme |  |  | 2,27E-02 |
| RSF1 | -0,028 | transcription regulator |  |  | 2,27E-02 |
| CYP46A1 | -0,345 | enzyme |  |  | 2,27E-02 |
| Vla-4 |  | complex |  |  | 2,27E-02 |
| carvacrol |  | chemical - endogenous non-mammalian |  |  | 2,27E-02 |
| MAL |  | other |  |  | 2,27E-02 |
| LOC100506098 |  | other |  |  | 2,27E-02 |
| LUCAT1 |  | other |  |  | 2,27E-02 |
| CD93 |  | other |  |  | 2,27E-02 |
| RNF34 | 0,115 | enzyme |  |  | 2,27E-02 |
| TLN1 | 0,057 | other |  |  | 2,27E-02 |
| mir-1275 |  | microRNA |  |  | 2,27E-02 |
| TIMP4 |  | other |  |  | 2,27E-02 |
| RNF4 | -0,045 | transcription regulator |  |  | 2,27E-02 |
| NRP2 | -0,025 | kinase |  |  | 2,27E-02 |
| CD59 |  | other |  |  | 2,27E-02 |
| CANX | 0,025 | other |  |  | 2,27E-02 |
| ANKRD1 | -0,061 | transcription regulator |  |  | 2,27E-02 |
| talazoparib |  | chemical drug |  |  | 2,27E-02 |
| STK17B | 0,008 | kinase |  |  | 2,27E-02 |
| CC-122 |  | chemical drug |  |  | 2,27E-02 |
| scoparone |  | chemical - endogenous non-mammalian |  |  | 2,27E-02 |
| sulfamethoxazole/trimethoprim |  | chemical drug |  |  | 2,27E-02 |
| lindane |  | chemical drug |  |  | 2,27E-02 |
| atrasentan |  | chemical drug |  |  | 2,27E-02 |
| CB-PIC |  | chemical reagent |  |  | 2,27E-02 |
| cannabinoid |  | chemical drug |  |  | 2,27E-02 |
| ampelopsin |  | chemical drug |  |  | 2,27E-02 |
| azelaic acid |  | chemical - endogenous mammalian |  |  | 2,27E-02 |
| phloroglucinol |  | chemical drug |  |  | 2,27E-02 |
| 5-hydroxytryptophan |  | chemical - endogenous mammalian |  |  | 2,27E-02 |
| Z-LLL-CHO |  | chemical - protease inhibitor |  | -0,776 | 2,31E-02 |
| ABCB1 | 0,262 | transporter |  |  | 2,46E-02 |
| BAX | -0,098 | transporter |  |  | 2,46E-02 |
| NOG | -0,184 | growth factor |  |  | 2,55E-02 |
| 3-nitropropionic acid |  | chemical toxicant |  |  | 2,55E-02 |
| bucladesine |  | chemical toxicant |  | 0,900 | 2,62E-02 |
| oblimersen |  | biologic drug |  |  | 2,64E-02 |
| carboplatin |  | chemical drug |  |  | 2,64E-02 |
| 1,2-dioleoyloxy-3-(trimethylammonium)propane |  | chemical reagent |  |  | 2,72E-02 |
| CYD0618 |  | chemical reagent |  |  | 2,72E-02 |
| Iberiotoxin |  | chemical reagent |  |  | 2,72E-02 |
| P110 |  | group |  |  | 2,72E-02 |
| givinostat |  | chemical drug |  |  | 2,72E-02 |
| HAND2-AS1 |  | other |  |  | 2,72E-02 |
| USP48 | 0,070 | peptidase |  |  | 2,72E-02 |
| RASSF3 | -0,026 | other |  |  | 2,72E-02 |
| EN2 |  | transcription regulator |  |  | 2,72E-02 |
| CLEC12A |  | other |  |  | 2,72E-02 |
| Ap2 alpha |  | group |  |  | 2,72E-02 |
| lumefantrine |  | chemical drug |  |  | 2,72E-02 |
| MAP4K1 | 0,436 | kinase |  |  | 2,72E-02 |
| DHX36 | 0,023 | enzyme |  |  | 2,72E-02 |
| BAD | -0,044 | other |  |  | 2,72E-02 |
| PROK2 |  | other |  |  | 2,72E-02 |
| HAS1 |  | enzyme |  |  | 2,72E-02 |
| APAF1 | -0,003 | other |  |  | 2,72E-02 |
| KAT7 | -0,002 | enzyme |  |  | 2,72E-02 |
| LRIG1 | 0,101 | other |  |  | 2,72E-02 |
| DNAJA3 | -0,144 | other |  |  | 2,72E-02 |
| COL4A1 | 0,010 | other |  |  | 2,72E-02 |
| MEST | -0,069 | peptidase |  |  | 2,72E-02 |
| MATK |  | kinase |  |  | 2,72E-02 |
| Ifna4 |  | other |  |  | 2,72E-02 |
| BCL2A1 |  | other |  |  | 2,72E-02 |
| fenebrutinib |  | chemical drug |  |  | 2,72E-02 |
| MIA-459 |  | chemical reagent |  |  | 2,72E-02 |
| MIA-313 |  | chemical reagent |  |  | 2,72E-02 |
| CIMO |  | chemical reagent |  |  | 2,72E-02 |
| ifosfamide |  | chemical drug |  |  | 2,72E-02 |
| propyl-2-(8-(3,4-difluorobenzyl)-2',5'-dioxo-8-azaspiro[bicyclo[3.2.1] octane-3,4'-imidazolidine]-1'-yl)acetate |  | chemical reagent |  |  | 2,72E-02 |
| sparfosic acid |  | chemical drug |  |  | 2,72E-02 |
| KL |  | enzyme |  |  | 2,73E-02 |
| indomethacin |  | chemical drug |  |  | 2,74E-02 |
| P38 MAPK |  | group |  |  | 2,98E-02 |
| PPP2CA | -0,014 | phosphatase |  |  | 3,10E-02 |
| NEUROD1 | -1,205 | transcription regulator |  |  | 3,10E-02 |
| STAT2 | 0,050 | transcription regulator |  |  | 3,10E-02 |
| anisomycin |  | chemical - endogenous non-mammalian |  |  | 3,10E-02 |
| bortezomib |  | chemical drug |  | -1,916 | 3,10E-02 |
| GNE-495 |  | chemical drug |  |  | 3,16E-02 |
| doramapimod |  | chemical drug |  |  | 3,16E-02 |
| SLC5A8 |  | transporter |  |  | 3,16E-02 |
| SFRP5 | -0,035 | transmembrane receptor |  |  | 3,16E-02 |
| ASCL2 |  | transcription regulator |  |  | 3,16E-02 |
| PDGFD | 0,111 | growth factor |  |  | 3,16E-02 |
| IER3 | -0,490 | other |  |  | 3,16E-02 |
| SELL | -0,000 | transmembrane receptor |  |  | 3,16E-02 |
| HMGCR | 0,032 | enzyme |  |  | 3,16E-02 |
| SNCB | -1,056 | other |  |  | 3,16E-02 |
| RBX1 | 0,017 | enzyme |  |  | 3,16E-02 |
| IL17B | -1,004 | cytokine |  |  | 3,16E-02 |
| ADCY5 | 0,026 | enzyme |  |  | 3,16E-02 |
| GNL1 | 0,061 | other |  |  | 3,16E-02 |
| TRIM14 | 0,265 | other |  |  | 3,16E-02 |
| casticin |  | chemical - endogenous non-mammalian |  |  | 3,16E-02 |
| ITGB1BP1 | 0,049 | other |  |  | 3,16E-02 |
| PGK1 | -0,017 | kinase |  |  | 3,16E-02 |
| nicorandil |  | chemical drug |  |  | 3,16E-02 |
| selegiline |  | chemical drug |  |  | 3,16E-02 |
| gliotoxin |  | chemical toxicant |  |  | 3,16E-02 |
| diacerein |  | chemical drug |  |  | 3,16E-02 |
| retroinverso ERG inhibitory peptide 2 |  | chemical reagent |  |  | 3,16E-02 |
| retroinverso ERG inhibitory peptide 1 |  | chemical reagent |  |  | 3,16E-02 |
| miR-124 mimic |  | chemical reagent |  |  | 3,16E-02 |
| rutin |  | chemical drug |  |  | 3,16E-02 |
| noladin ether |  | chemical - endogenous mammalian |  |  | 3,16E-02 |
| TNFRSF8 | -0,271 | transmembrane receptor |  |  | 3,19E-02 |
| N-[N-(3,5-difluorophenacetyl-L-Ala)]-S-phenylglycine t-butyl ester |  | chemical - protease inhibitor |  |  | 3,19E-02 |
| CXCR4 | 3,314 | G-protein coupled receptor |  |  | 3,39E-02 |
| MSC | 0,067 | transcription regulator |  |  | 3,49E-02 |
| emodin |  | chemical drug |  |  | 3,49E-02 |
| IL2RG |  | transmembrane receptor |  |  | 3,59E-02 |
| carbon monoxide |  | chemical - endogenous mammalian |  |  | 3,59E-02 |
| fingolimod phosphate |  | chemical - endogenous mammalian |  |  | 3,61E-02 |
| HCFC1 | -0,074 | transcription regulator |  |  | 3,61E-02 |
| rhodamine 6G |  | chemical toxicant |  |  | 3,61E-02 |
| cosyntropin |  | biologic drug |  |  | 3,61E-02 |
| HVCN1 | -0,922 | ion channel |  |  | 3,61E-02 |
| NRAD1 |  | other |  |  | 3,61E-02 |
| SULT2B1 |  | enzyme |  |  | 3,61E-02 |
| PDS5B | 0,026 | other |  |  | 3,61E-02 |
| ABCC1 | 0,078 | transporter |  |  | 3,61E-02 |
| HINT1 | -1,197 | enzyme |  |  | 3,61E-02 |
| GRK5 | 0,002 | kinase |  |  | 3,61E-02 |
| CXCR1 |  | G-protein coupled receptor |  |  | 3,61E-02 |
| RLN1 |  | other |  |  | 3,61E-02 |
| CYSLTR1 | 0,000 | G-protein coupled receptor |  |  | 3,61E-02 |
| ponesimod |  | chemical drug |  |  | 3,61E-02 |
| LAMA4 | 0,029 | enzyme |  |  | 3,61E-02 |
| ARAF | 0,037 | kinase |  |  | 3,61E-02 |
| MAD2L1 |  | other |  |  | 3,61E-02 |
| NEU3 | -0,022 | enzyme |  |  | 3,61E-02 |
| CCX771 |  | chemical reagent |  |  | 3,61E-02 |
| TSPO | 0,021 | transmembrane receptor |  |  | 3,61E-02 |
| MM-401 |  | chemical reagent |  |  | 3,61E-02 |
| cholestyramine |  | chemical drug |  |  | 3,61E-02 |
| naproxen |  | chemical drug |  |  | 3,61E-02 |
| pranlukast |  | chemical drug |  |  | 3,61E-02 |
| vandetanib |  | chemical drug |  |  | 3,61E-02 |
| ETV6-NTRK3 |  | fusion gene/product |  |  | 3,61E-02 |
| retinaldehyde |  | chemical - endogenous mammalian |  |  | 3,61E-02 |
| levofloxacin |  | chemical drug |  |  | 3,61E-02 |
| huperzine A |  | chemical drug |  |  | 3,61E-02 |
| citric acid |  | chemical - endogenous mammalian |  |  | 3,61E-02 |
| alpha-tocopherol succinate |  | chemical drug |  |  | 3,61E-02 |
| glucosamine |  | chemical - endogenous mammalian |  |  | 3,69E-02 |
| LDL |  | complex |  | -1,715 | 3,78E-02 |
| TLR4 | 0,300 | transmembrane receptor |  | 0,254 | 3,80E-02 |
| HNF4A | -1,206 | transcription regulator |  |  | 3,84E-02 |
| Ngf |  | group |  |  | 3,89E-02 |
| SOD1 | 0,000 | enzyme |  |  | 3,96E-02 |
| kainic acid |  | chemical toxicant |  |  | 3,97E-02 |
| prexasertib |  | chemical drug |  |  | 4,00E-02 |
| tannic acid |  | chemical toxicant |  |  | 4,05E-02 |
| phosphatidylcholine |  | chemical - endogenous mammalian |  |  | 4,05E-02 |
| bazedoxifene |  | chemical drug |  |  | 4,05E-02 |
| Mir218 |  | microRNA |  |  | 4,05E-02 |
| Ck2 |  | complex |  |  | 4,05E-02 |
| cytidylyl-3'-5'-guanosine |  | chemical reagent |  |  | 4,05E-02 |
| propargylamine |  | chemical reagent |  |  | 4,05E-02 |
| ZBED2 | -0,577 | transcription regulator |  |  | 4,05E-02 |
| TACSTD2 | 0,000 | other |  |  | 4,05E-02 |
| SERPINB2 | 0,665 | other |  |  | 4,05E-02 |
| BCL11A |  | transcription regulator |  |  | 4,05E-02 |
| BRD1 | 0,066 | other |  |  | 4,05E-02 |
| DKK2 | -1,197 | other |  |  | 4,05E-02 |
| CDKN2B |  | transcription regulator |  |  | 4,05E-02 |
| RPS6KB2 | -0,070 | kinase |  |  | 4,05E-02 |
| ARG2 | -0,240 | enzyme |  |  | 4,05E-02 |
| PANDAR |  | other |  |  | 4,05E-02 |
| GPR183 |  | G-protein coupled receptor |  |  | 4,05E-02 |
| SHANK3 | 0,183 | other |  |  | 4,05E-02 |
| di-spiropyrrolizidino oxindole andrographolide derivative CY2 |  | chemical reagent |  |  | 4,05E-02 |
| CSNK1A1 | -0,018 | kinase |  |  | 4,05E-02 |
| BRMS1 | -0,018 | transcription regulator |  |  | 4,05E-02 |
| SCH772984 |  | chemical drug |  |  | 4,05E-02 |
| dantrolene |  | chemical drug |  |  | 4,05E-02 |
| benazepril |  | chemical drug |  |  | 4,05E-02 |
| ammonium trichloro(dioxoethylene-O,O'-)tellurate |  | chemical drug |  |  | 4,05E-02 |
| brimonidine |  | chemical drug |  |  | 4,05E-02 |
| PD 168393 |  | chemical drug |  |  | 4,05E-02 |
| prodigiosin |  | chemical toxicant |  |  | 4,05E-02 |
| ziprasidone |  | chemical drug |  |  | 4,05E-02 |
| tyrphostin AG 127 |  | chemical drug |  |  | 4,05E-02 |
| ITGB3 | -0,016 | transmembrane receptor |  |  | 4,11E-02 |
| fulvestrant |  | chemical drug |  | -0,684 | 4,13E-02 |
| Immunoglobulin |  | complex |  | -0,063 | 4,20E-02 |
| IKZF3 |  | transcription regulator |  |  | 4,21E-02 |
| lipoteichoic acid |  | chemical - endogenous non-mammalian |  |  | 4,21E-02 |
| gemcitabine |  | chemical drug |  |  | 4,32E-02 |
| EZH2 | -0,005 | transcription regulator |  | -0,200 | 4,33E-02 |
| RICTOR | -0,006 | other |  | -0,152 | 4,36E-02 |
| tyrphostin AG 1478 |  | chemical drug |  |  | 4,43E-02 |
| IL5 |  | cytokine |  | 0,000 | 4,45E-02 |
| lnc-HAND2-2 |  | other |  |  | 4,49E-02 |
| niclosamide |  | chemical drug |  |  | 4,49E-02 |
| Stat1-Stat2 |  | complex |  |  | 4,49E-02 |
| Glucocorticoid-GCR |  | complex |  |  | 4,49E-02 |
| NAP1L1 |  | other |  |  | 4,49E-02 |
| Shc |  | group |  |  | 4,49E-02 |
| necrostatin-1 |  | chemical reagent |  |  | 4,49E-02 |
| PHF21A | -0,007 | other |  |  | 4,49E-02 |
| nirogacestat |  | chemical drug |  |  | 4,49E-02 |
| GFPT1 | 0,011 | enzyme |  |  | 4,49E-02 |
| TNFRSF17 |  | transmembrane receptor |  |  | 4,49E-02 |
| AGRP |  | other |  |  | 4,49E-02 |
| mir-503 |  | microRNA |  |  | 4,49E-02 |
| UGDH | -0,107 | enzyme |  |  | 4,49E-02 |
| RANBP9 | 0,000 | other |  |  | 4,49E-02 |
| SLC6A1 |  | transporter |  |  | 4,49E-02 |
| SIAH1 | -0,015 | enzyme |  |  | 4,49E-02 |
| piroxicam |  | chemical drug |  |  | 4,49E-02 |
| levamisole |  | chemical drug |  |  | 4,49E-02 |
| PTH |  | other |  |  | 4,73E-02 |
| minocycline |  | chemical drug |  |  | 4,76E-02 |
| tetracycline |  | chemical drug |  |  | 4,76E-02 |
| Hif1 |  | complex |  |  | 4,76E-02 |
| PIAS1 | -0,046 | transcription regulator |  |  | 4,87E-02 |
| ursolic acid |  | chemical drug |  |  | 4,87E-02 |
| UDP |  | chemical - endogenous mammalian |  |  | 4,93E-02 |
| AGO1 | -0,106 | translation regulator |  |  | 4,93E-02 |
| ganetespib |  | chemical drug |  |  | 4,93E-02 |
| olaparib |  | chemical drug |  |  | 4,93E-02 |
| BMS-754807 |  | chemical drug |  |  | 4,93E-02 |
| Mcpt4 |  | peptidase |  |  | 4,93E-02 |
| MCL1 | 0,074 | transporter |  |  | 4,93E-02 |
| (-)-arctigenin |  | chemical reagent |  |  | 4,93E-02 |
| DNM1L | -0,059 | enzyme |  |  | 4,93E-02 |
| LINC-ROR |  | other |  |  | 4,93E-02 |
| LNX2 | 0,409 | other |  |  | 4,93E-02 |
| GADD45B | 0,178 | other |  |  | 4,93E-02 |
| ERRFI1 | 0,030 | other |  |  | 4,93E-02 |
| MANF | -0,035 | other |  |  | 4,93E-02 |
| CHRNA3 | -0,414 | transmembrane receptor |  |  | 4,93E-02 |
| lidocaine |  | chemical drug |  |  | 4,93E-02 |
| kukoamine A |  | chemical - endogenous non-mammalian |  |  | 4,93E-02 |
| 7-(4-fluorobenzylamino)-1,3,4,8-tetrahydropyrrolo[4,3,2-de]quinolin-8(1H)-one |  | chemical reagent |  |  | 4,93E-02 |
| latrunculin A |  | chemical toxicant |  |  | 4,93E-02 |
| SS18-SSX2 |  | fusion gene/product |  |  | 4,93E-02 |
| astragaloside IV |  | chemical - endogenous non-mammalian |  |  | 4,93E-02 |
| CA074-methyl ester |  | chemical reagent |  |  | 4,93E-02 |
| delphinidin |  | chemical - endogenous non-mammalian |  |  | 4,93E-02 |
| hesperetin |  | chemical drug |  |  | 4,93E-02 |
| puerarin |  | chemical drug |  |  | 4,93E-02 |
| iodine |  | chemical - endogenous mammalian |  |  | 4,93E-02 |
| N,N-dimethylarginine |  | chemical - endogenous mammalian |  |  | 4,93E-02 |
| nandrolone |  | chemical drug |  |  | 4,93E-02 |
| trestolone |  | chemical drug |  |  | 4,93E-02 |
| MYB | 0,062 | transcription regulator |  |  | 4,93E-02 |
| IFNB1 |  | cytokine |  |  | 4,93E-02 |
| CREB1 | -0,004 | transcription regulator |  | 0,338 | 4,96E-02 |
| PRKCE | 0,033 | kinase |  |  | 4,99E-02 |
| PRNP | -0,108 | other |  |  | 4,99E-02 |
| ROR2 | -0,003 | kinase |  |  | 4,99E-02 |
| tetrachlorodibenzodioxin |  | chemical toxicant |  | -1,886 | 7,29E-02 |
| IL2 |  | cytokine |  | 1,919 | 1,11E-01 |

| Melatonin + N-acetyl serotonin | | | | | |
| --- | --- | --- | --- | --- | --- |
| Upstream Regulator | **Expr Log Ratio** | **Molecule Type** | **Predicted Activation State** | **Activation z-score** | **p-value of overlap** |
| DTNA | -0,022 | other |  |  | 7,76E-05 |
| MFAP5 | 0,107 | other |  | -0,152 | 1,69E-04 |
| norethindrone acetate |  | chemical drug |  |  | 3,84E-04 |
| PDZK1 | -0,418 | other |  |  | 7,12E-04 |
| CAV1 | -0,052 | transmembrane receptor |  |  | 1,19E-03 |
| SP600125 |  | chemical drug |  | -1,709 | 1,29E-03 |
| FOXD1 |  | transcription regulator |  |  | 1,38E-03 |
| USP2 | -0,397 | peptidase |  |  | 1,38E-03 |
| NUMBL | 0,046 | other |  |  | 1,66E-03 |
| Laminin5 |  | complex |  |  | 1,66E-03 |
| mir-30 |  | microRNA |  |  | 2,27E-03 |
| CCN2 | 0,099 | growth factor |  |  | 2,47E-03 |
| ABCB4 | -0,411 | transporter |  |  | 2,54E-03 |
| SIAH2 | 0,059 | transcription regulator |  |  | 2,61E-03 |
| cisplatin |  | chemical drug |  | -0,363 | 2,68E-03 |
| SPARC | 0,103 | other |  | 0,218 | 2,82E-03 |
| anisomycin |  | chemical - endogenous non-mammalian |  |  | 3,61E-03 |
| E2F6 | 0,030 | transcription regulator |  |  | 4,14E-03 |
| CEBPB |  | transcription regulator |  | -1,195 | 4,23E-03 |
| 9-hydroxy-(S)-10,12-octadecadienoic acid |  | chemical - endogenous mammalian |  |  | 5,12E-03 |
| PRPF31 | 0,030 | other |  |  | 5,12E-03 |
| ADTRP |  | enzyme |  |  | 5,12E-03 |
| black cohosh extract |  | chemical drug |  |  | 5,12E-03 |
| USP39 | -0,013 | peptidase |  |  | 5,12E-03 |
| PRCC | 0,032 | other |  |  | 5,12E-03 |
| DYNLRB1 | -0,004 | other |  |  | 5,12E-03 |
| PAIP2 | 0,008 | translation regulator |  |  | 5,12E-03 |
| UHMK1 | -0,008 | kinase |  |  | 5,12E-03 |
| PAM | 0,091 | enzyme |  |  | 5,12E-03 |
| mir-617 |  | microRNA |  |  | 5,12E-03 |
| LINC00278 |  | other |  |  | 5,12E-03 |
| NKX2-8 | -1,748 | transcription regulator |  |  | 5,12E-03 |
| GDF10 | 0,000 | growth factor |  |  | 5,12E-03 |
| UBL5 | -0,172 | other |  |  | 5,12E-03 |
| MATN3 | -0,077 | other |  |  | 5,12E-03 |
| MTARC2 |  | enzyme |  |  | 5,12E-03 |
| mitoguazone |  | chemical drug |  |  | 5,12E-03 |
| pegademase bovine |  | biologic drug |  |  | 5,12E-03 |
| SMAD3-EP300 |  | complex |  |  | 5,12E-03 |
| DICER1 | -0,007 | enzyme |  | 0,128 | 5,34E-03 |
| ODC1 | -0,027 | enzyme |  |  | 5,61E-03 |
| GnRH-A |  | chemical reagent |  |  | 6,02E-03 |
| HTATIP2 | -0,061 | transcription regulator |  |  | 6,12E-03 |
| Brd4 |  | kinase |  |  | 6,50E-03 |
| chrysin |  | chemical - endogenous non-mammalian |  |  | 6,65E-03 |
| UBE3A | -0,002 | enzyme |  |  | 7,21E-03 |
| BCL6 | 0,023 | transcription regulator |  |  | 7,76E-03 |
| SFTPA1 |  | transporter |  |  | 7,78E-03 |
| miR-23a-3p (and other miRNAs w/seed UCACAUU) |  | mature microRNA |  |  | 7,78E-03 |
| Fus | -0,091 | transcription regulator |  |  | 7,78E-03 |
| NOTCH4 | -0,279 | transcription regulator |  |  | 8,38E-03 |
| NFIX | 0,029 | transcription regulator |  |  | 8,38E-03 |
| MACROH2A1 | -0,038 | other |  |  | 8,62E-03 |
| collagen type i (family) |  | group |  |  | 8,99E-03 |
| ADA | -0,129 | enzyme |  |  | 8,99E-03 |
| SNAI1 | -0,259 | transcription regulator |  | -0,849 | 9,08E-03 |
| EML4-ALK |  | fusion gene/product |  |  | 1,01E-02 |
| miglitol |  | chemical drug |  |  | 1,02E-02 |
| cytochrome-c oxidase |  | complex |  |  | 1,02E-02 |
| HLTF | -0,004 | transcription regulator |  |  | 1,02E-02 |
| PRPF8 | -0,012 | other |  |  | 1,02E-02 |
| KCNJ6 | 0,057 | ion channel |  |  | 1,02E-02 |
| ING2 | -0,014 | transcription regulator |  |  | 1,02E-02 |
| BOC | 0,033 | other |  |  | 1,02E-02 |
| DBF4B | -0,334 | other |  |  | 1,02E-02 |
| MYO9B | 0,033 | enzyme |  |  | 1,02E-02 |
| MSH3 | 0,026 | enzyme |  |  | 1,02E-02 |
| CRTAP | -0,011 | other |  |  | 1,02E-02 |
| RNF11 | 0,011 | enzyme |  |  | 1,02E-02 |
| MATN1 | -0,904 | other |  |  | 1,02E-02 |
| ZFYVE16 | -0,008 | other |  |  | 1,02E-02 |
| 1,5-bis-(dihexyl-N-nitrosoamino)-2,4-dinitrobenzene |  | chemical reagent |  |  | 1,02E-02 |
| sarpogrelate |  | chemical drug |  |  | 1,02E-02 |
| methiothepin |  | chemical drug |  |  | 1,02E-02 |
| sibutramine |  | chemical drug |  |  | 1,02E-02 |
| PSB-1115 |  | chemical reagent |  |  | 1,02E-02 |
| synthetic peptide |  | chemical reagent |  |  | 1,02E-02 |
| vitamin K1 |  | chemical - endogenous mammalian |  |  | 1,02E-02 |
| tibolone |  | chemical drug |  |  | 1,02E-02 |
| SBDS | 0,097 | other |  |  | 1,04E-02 |
| LGALS1 | 0,005 | other |  |  | 1,04E-02 |
| MITF | 0,069 | transcription regulator | Inhibited | -2,000 | 1,09E-02 |
| MECOM | -0,004 | transcription regulator |  |  | 1,10E-02 |
| fludarabine |  | chemical drug |  |  | 1,10E-02 |
| MEF2 |  | group |  |  | 1,24E-02 |
| medroxyprogesterone acetate |  | chemical drug |  | -1,633 | 1,24E-02 |
| HNF1A | -0,234 | transcription regulator | Inhibited | -2,000 | 1,28E-02 |
| IHH | 0,892 | enzyme |  |  | 1,28E-02 |
| SOX17 |  | transcription regulator |  |  | 1,31E-02 |
| GLIS2 | -0,027 | transcription regulator |  |  | 1,31E-02 |
| GAB2 | -0,020 | other |  |  | 1,31E-02 |
| MKNK1 | -0,166 | kinase |  | 0,000 | 1,33E-02 |
| palmitic acid |  | chemical - endogenous mammalian |  | -0,152 | 1,36E-02 |
| CST5 |  | other | Activated | 2,236 | 1,40E-02 |
| crizotinib |  | chemical drug |  |  | 1,46E-02 |
| NVP-TAE684 |  | chemical drug |  |  | 1,46E-02 |
| FUS-DDIT3 |  | fusion gene/product |  |  | 1,46E-02 |
| MIR100HG |  | other |  |  | 1,53E-02 |
| hexa-D-arginine |  | biologic drug |  |  | 1,53E-02 |
| ZNF451 | -0,069 | enzyme |  |  | 1,53E-02 |
| MLLT1 | 0,006 | transcription regulator |  |  | 1,53E-02 |
| HDGF | -0,025 | growth factor |  |  | 1,53E-02 |
| FERMT3 |  | enzyme |  |  | 1,53E-02 |
| defibrotide |  | biologic drug |  |  | 1,53E-02 |
| CSNK1E |  | kinase |  |  | 1,53E-02 |
| RAD51 | -0,281 | enzyme |  |  | 1,53E-02 |
| riociguat |  | chemical drug |  |  | 1,53E-02 |
| STK16 | 0,053 | kinase |  |  | 1,53E-02 |
| STRAP | 0,015 | other |  |  | 1,53E-02 |
| RPL37 | -0,080 | other |  |  | 1,53E-02 |
| SCT |  | other |  |  | 1,53E-02 |
| SNRNP70 | -0,092 | other |  |  | 1,53E-02 |
| filipin |  | chemical - endogenous non-mammalian |  |  | 1,53E-02 |
| tomelukast |  | chemical drug |  |  | 1,53E-02 |
| MIR23A/24-2/27A cluster |  | group |  |  | 1,53E-02 |
| TTI-101 |  | chemical drug |  |  | 1,53E-02 |
| TAPI |  | chemical - protease inhibitor |  |  | 1,53E-02 |
| mir23a/24-2/27a |  | group |  |  | 1,53E-02 |
| raffinose |  | chemical - endogenous mammalian |  |  | 1,53E-02 |
| CCAR2 | -0,023 | peptidase |  |  | 1,54E-02 |
| CIDEC | 0,039 | other |  |  | 1,70E-02 |
| sucrose |  | chemical - endogenous mammalian |  |  | 1,70E-02 |
| MMP3 |  | peptidase |  |  | 1,72E-02 |
| genistein |  | chemical drug |  | -0,057 | 1,84E-02 |
| DACH1 | 0,047 | transcription regulator |  |  | 1,87E-02 |
| ASXL1 | -0,116 | transcription regulator |  |  | 1,96E-02 |
| 13(S)-hydroxyoctadecadienoic acid |  | chemical - endogenous mammalian |  |  | 2,03E-02 |
| ENTPD2 | 0,730 | enzyme |  |  | 2,03E-02 |
| YTHDF1 | 0,021 | other |  |  | 2,03E-02 |
| VLDL |  | complex |  |  | 2,03E-02 |
| NXF1 | -0,072 | other |  |  | 2,03E-02 |
| SF3B1 | -0,047 | other |  |  | 2,03E-02 |
| miR-339-5p (and other miRNAs w/seed CCCUGUC) |  | mature microRNA |  |  | 2,03E-02 |
| LAMB1 | -0,011 | other |  |  | 2,03E-02 |
| SNX17 | 0,031 | transporter |  |  | 2,03E-02 |
| SEMA3E | -0,070 | other |  |  | 2,03E-02 |
| GIPC1 | 0,036 | other |  |  | 2,03E-02 |
| CPT2 | 0,100 | enzyme |  |  | 2,03E-02 |
| DDX19B | -0,060 | enzyme |  |  | 2,03E-02 |
| COL1A2 | 0,099 | other |  |  | 2,03E-02 |
| selinexor |  | chemical drug |  |  | 2,03E-02 |
| SYK023 |  | chemical reagent |  |  | 2,03E-02 |
| SKL-2001 |  | chemical reagent |  |  | 2,03E-02 |
| tyrphostin AG 18 |  | chemical drug |  |  | 2,03E-02 |
| long chain fatty acid |  | chemical - endogenous mammalian |  |  | 2,03E-02 |
| iodixanol |  | chemical drug |  |  | 2,03E-02 |
| ADORA2B | -0,473 | G-protein coupled receptor |  |  | 2,04E-02 |
| SRSF2 | 0,000 | transcription regulator |  |  | 2,04E-02 |
| SKIL | 0,016 | transcription regulator |  |  | 2,13E-02 |
| TGFB1 | 0,046 | growth factor |  | 0,934 | 2,19E-02 |
| eflornithine |  | chemical drug |  |  | 2,23E-02 |
| SMAD2 | -0,003 | transcription regulator |  |  | 2,28E-02 |
| ERBB2 | 0,032 | kinase |  | -1,219 | 2,31E-02 |
| cyclopamine |  | chemical reagent |  |  | 2,32E-02 |
| MASTL | -0,173 | kinase |  |  | 2,42E-02 |
| Integrinα |  | group |  |  | 2,53E-02 |
| BI 2536 |  | chemical drug |  |  | 2,53E-02 |
| edratide |  | biologic drug |  |  | 2,53E-02 |
| Ho |  | group |  |  | 2,53E-02 |
| SLC22A2 | 0,733 | transporter |  |  | 2,53E-02 |
| MLYCD | 0,054 | enzyme |  |  | 2,53E-02 |
| FOXN4 |  | transcription regulator |  |  | 2,53E-02 |
| 2,3-bis(3'-hydroxybenzyl)butane-1,4-diol |  | chemical - endogenous mammalian |  |  | 2,53E-02 |
| EIF3I | 0,000 | translation regulator |  |  | 2,53E-02 |
| TLN1 | 0,010 | other |  |  | 2,53E-02 |
| mir-590 |  | microRNA |  |  | 2,53E-02 |
| miR-508-3p (miRNAs w/seed GAUUGUA) |  | mature microRNA |  |  | 2,53E-02 |
| WNT2B | -0,181 | other |  |  | 2,53E-02 |
| BRCA2 | -0,236 | transcription regulator |  |  | 2,53E-02 |
| AGA | -0,013 | enzyme |  |  | 2,53E-02 |
| decanoic acid |  | chemical - endogenous mammalian |  |  | 2,53E-02 |
| KAT2A | -0,062 | enzyme |  |  | 2,53E-02 |
| beta-estradiol |  | chemical - endogenous mammalian |  | -1,870 | 2,70E-02 |
| CRP |  | other |  |  | 2,71E-02 |
| MYC | -0,035 | transcription regulator |  | 0,781 | 2,77E-02 |
| CDKN1B | -0,006 | kinase |  |  | 2,81E-02 |
| 8-chlorophenylthio-adenosine 3',5'-cyclic monophosphate |  | chemical reagent |  |  | 2,81E-02 |
| ID2 | 0,069 | transcription regulator |  |  | 2,90E-02 |
| CEBPA |  | transcription regulator |  | 0,441 | 2,99E-02 |
| CCN5 | 0,328 | growth factor |  |  | 3,02E-02 |
| mGLUR Group I |  | group |  |  | 3,03E-02 |
| LINC00842 |  | other |  |  | 3,03E-02 |
| DPPA3 |  | other |  |  | 3,03E-02 |
| ZMIZ1 | -0,000 | transcription regulator |  |  | 3,03E-02 |
| FOXK2 | 0,039 | transcription regulator |  |  | 3,03E-02 |
| ACOT13 |  | enzyme |  |  | 3,03E-02 |
| miR-320b (and other miRNAs w/seed AAAGCUG) |  | mature microRNA |  |  | 3,03E-02 |
| PHGDH | -0,041 | enzyme |  |  | 3,03E-02 |
| RNF111 | 0,014 | enzyme |  |  | 3,03E-02 |
| NUDT21 | 0,005 | other |  |  | 3,03E-02 |
| TRIM33 | -0,005 | transcription regulator |  |  | 3,03E-02 |
| EFEMP2 | 0,072 | other |  |  | 3,03E-02 |
| RPS20 | -0,058 | other |  |  | 3,03E-02 |
| NET1 | -0,062 | other |  |  | 3,03E-02 |
| CXXC4 |  | other |  |  | 3,03E-02 |
| acetazolamide |  | chemical drug |  |  | 3,03E-02 |
| BML-284 |  | chemical reagent |  |  | 3,03E-02 |
| pyridoxamine |  | chemical - endogenous mammalian |  |  | 3,03E-02 |
| eprosartan |  | chemical drug |  |  | 3,03E-02 |
| EPZ005687 |  | chemical - protease inhibitor |  |  | 3,03E-02 |
| GRIN1 |  | ion channel |  |  | 3,23E-02 |
| ADAM10 | 0,002 | peptidase |  |  | 3,52E-02 |
| VIPAS39 | -0,012 | other |  |  | 3,53E-02 |
| WNT16 | 0,116 | other |  |  | 3,53E-02 |
| AZD4547 |  | chemical drug |  |  | 3,53E-02 |
| ERCC2 | -0,079 | enzyme |  |  | 3,53E-02 |
| COMT | 0,080 | enzyme |  |  | 3,53E-02 |
| SNTA1 | 0,081 | other |  |  | 3,53E-02 |
| WNK1 | 0,009 | kinase |  |  | 3,53E-02 |
| TSC22D1 |  | transcription regulator |  |  | 3,53E-02 |
| DLC1 | -0,047 | other |  |  | 3,53E-02 |
| SLC22A1 |  | transporter |  |  | 3,53E-02 |
| GUCY2F | 0,000 | kinase |  |  | 3,53E-02 |
| TP53INP1 | 0,030 | other |  |  | 3,53E-02 |
| ERBIN | -0,055 | other |  |  | 3,53E-02 |
| ITGB1BP1 | 0,136 | other |  |  | 3,53E-02 |
| H4C3 |  | other |  |  | 3,53E-02 |
| Brd4 |  | kinase |  |  | 3,53E-02 |
| thenoyltrifluoroacetone |  | chemical reagent |  |  | 3,53E-02 |
| diacerein |  | chemical drug |  |  | 3,53E-02 |
| miR-508-3p inhibitor |  | chemical reagent |  |  | 3,53E-02 |
| SP110 | 0,374 | transcription regulator |  |  | 3,59E-02 |
| FGF21 |  | growth factor |  |  | 3,67E-02 |
| ACSL4 | 0,010 | enzyme |  |  | 3,79E-02 |
| valsartan |  | chemical drug |  |  | 3,90E-02 |
| SB203580 |  | chemical drug |  | 0,059 | 3,96E-02 |
| NR3C2 | -0,122 | ligand-dependent nuclear receptor |  |  | 3,98E-02 |
| meldonium |  | chemical drug |  |  | 4,02E-02 |
| fingolimod phosphate |  | chemical - endogenous mammalian |  |  | 4,02E-02 |
| cosyntropin |  | biologic drug |  |  | 4,02E-02 |
| CHCHD5 | -0,043 | other |  |  | 4,02E-02 |
| linsitinib |  | chemical drug |  |  | 4,02E-02 |
| ANGPTL8 | -2,150 | other |  |  | 4,02E-02 |
| PON2 | 0,044 | enzyme |  |  | 4,02E-02 |
| BAG6 | -0,001 | enzyme |  |  | 4,02E-02 |
| MIA |  | other |  |  | 4,02E-02 |
| SOX18 | -0,360 | transcription regulator |  |  | 4,02E-02 |
| ponesimod |  | chemical drug |  |  | 4,02E-02 |
| EFEMP1 | 0,147 | enzyme |  |  | 4,02E-02 |
| LAMA4 | -0,068 | enzyme |  |  | 4,02E-02 |
| LPA |  | other |  |  | 4,02E-02 |
| DKC1 | 0,034 | enzyme |  |  | 4,02E-02 |
| SNW1 | -0,012 | transcription regulator |  |  | 4,02E-02 |
| GADD45GIP1 | 0,011 | other |  |  | 4,02E-02 |
| proteasome inhibitor |  | chemical drug |  |  | 4,02E-02 |
| MM-401 |  | chemical reagent |  |  | 4,02E-02 |
| GW 5074 |  | chemical drug |  |  | 4,02E-02 |
| QKI | -0,018 | other |  |  | 4,02E-02 |
| PPP3R1 | -0,059 | phosphatase |  |  | 4,02E-02 |
| PTGES | -2,109 | enzyme |  |  | 4,02E-02 |
| rosiglitazone |  | chemical drug | Inhibited | -2,164 | 4,02E-02 |
| miR-204-5p (and other miRNAs w/seed UCCCUUU) |  | mature microRNA |  |  | 4,14E-02 |
| thioacetamide |  | chemical toxicant |  |  | 4,17E-02 |
| ATF6 | 0,025 | transcription regulator |  |  | 4,26E-02 |
| GFI1 | -0,114 | transcription regulator |  |  | 4,33E-02 |
| RUNX1 | 0,025 | transcription regulator |  |  | 4,42E-02 |
| miR-30c-5p (and other miRNAs w/seed GUAAACA) |  | mature microRNA |  |  | 4,47E-02 |
| 11-dehydrocorticosterone |  | chemical - endogenous mammalian |  |  | 4,51E-02 |
| AFF4 | 0,016 | transcription regulator |  |  | 4,51E-02 |
| S100A10 | -0,021 | other |  |  | 4,51E-02 |
| bicyclol |  | chemical drug |  |  | 4,51E-02 |
| DLX1 | -0,259 | transcription regulator |  |  | 4,51E-02 |
| DNM3OS |  | other |  |  | 4,51E-02 |
| CDKN2B |  | transcription regulator |  |  | 4,51E-02 |
| STK25 | 0,022 | kinase |  |  | 4,51E-02 |
| SCUBE3 | 0,124 | other |  |  | 4,51E-02 |
| SERPINH1 | 0,073 | other |  |  | 4,51E-02 |
| aurothioglucose |  | chemical drug |  |  | 4,51E-02 |
| FGF2 | -0,040 | growth factor |  | -0,568 | 4,58E-02 |
| NFAT5 | -0,083 | transcription regulator |  |  | 4,61E-02 |
| diphenyleneiodonium |  | chemical reagent |  |  | 4,63E-02 |
| methylprednisolone |  | chemical drug |  | -0,585 | 4,73E-02 |
| IL6ST | 0,096 | transmembrane receptor |  |  | 4,75E-02 |
| HOXD10 | -0,226 | transcription regulator |  |  | 4,88E-02 |
| F3 | -0,165 | transmembrane receptor |  |  | 4,88E-02 |
| SIRT1 | -0,011 | transcription regulator | Inhibited | -2,219 | 7,86E-02 |
| JUN | 0,040 | transcription regulator |  | 1,946 | 2,58E-01 |
| AGT |  | growth factor | Activated | 2,129 | 3,22E-01 |
| KRAS | -0,011 | enzyme | Inhibited | -2,000 | 1,00E00 |
| decitabine |  | chemical drug |  | 1,982 | 1,00E00 |

| Melatonin + S26131 (10^-9^ M) treatment | | | | | |
| --- | --- | --- | --- | --- | --- |
| Upstream Regulator | **Expr Log Ratio** | **Molecule Type** | **Predicted Activation State** | **Activation z-score** | **p-value of overlap** |
| Cyclin B |  | group |  |  | 1,54E-04 |
| idronoxil |  | chemical drug |  |  | 1,54E-04 |
| RSF1 | -0,066 | transcription regulator |  |  | 5,09E-04 |
| PROKR2 | -1,291 | G-protein coupled receptor |  |  | 7,60E-04 |
| PTK6 | 0,870 | kinase |  |  | 1,06E-03 |
| casticin |  | chemical - endogenous non-mammalian |  |  | 1,06E-03 |
| DIABLO | 0,102 | other |  |  | 1,40E-03 |
| proteasome inhibitor |  | chemical drug |  |  | 1,40E-03 |
| perifosine |  | chemical drug |  |  | 1,40E-03 |
| mir-193 |  | microRNA |  |  | 1,56E-03 |
| TGFB2 | -0,028 | growth factor | Inhibited | -2,208 | 1,60E-03 |
| ITGB3 | -0,167 | transmembrane receptor |  |  | 1,65E-03 |
| PRKD1 | -0,016 | kinase |  | -0,192 | 1,92E-03 |
| NQO2 | -1,055 | enzyme |  |  | 2,24E-03 |
| USP2 | -0,017 | peptidase |  |  | 2,72E-03 |
| CLOCK | -0,303 | transcription regulator |  | -0,447 | 2,79E-03 |
| mir-8 |  | microRNA | Activated | 2,230 | 2,80E-03 |
| ABL1 | -0,048 | kinase |  |  | 3,20E-03 |
| embelin |  | chemical - endogenous non-mammalian |  |  | 3,25E-03 |
| EFNB1 | 0,048 | other |  |  | 3,25E-03 |
| quinacrine |  | chemical drug |  |  | 3,25E-03 |
| CSNK2B | 0,167 | kinase |  |  | 3,82E-03 |
| ACVR2A | -0,085 | kinase |  |  | 3,82E-03 |
| PKD1 | 0,035 | ion channel |  | 0,447 | 3,86E-03 |
| STOX1 | -0,115 | other |  |  | 4,44E-03 |
| noscapine |  | chemical drug |  |  | 4,44E-03 |
| temozolomide |  | chemical drug |  |  | 4,45E-03 |
| fulvestrant |  | chemical drug |  | -0,728 | 4,53E-03 |
| lonafarnib |  | chemical drug |  |  | 5,10E-03 |
| 1'-acetoxychavicol acetate |  | chemical reagent |  |  | 5,10E-03 |
| NFAT5 | -0,245 | transcription regulator |  | -1,467 | 5,51E-03 |
| Pdgf (complex) |  | complex |  | -1,214 | 6,18E-03 |
| flavokawain C |  | chemical - endogenous non-mammalian |  |  | 7,21E-03 |
| methyl jasmonate |  | chemical - endogenous non-mammalian |  |  | 7,21E-03 |
| omigapil |  | chemical drug |  |  | 7,21E-03 |
| glutathione peroxidase |  | group |  |  | 7,21E-03 |
| TFIIF |  | complex |  |  | 7,21E-03 |
| Max-Myc |  | complex |  |  | 7,21E-03 |
| ethylphenylhydantoin |  | chemical - endogenous mammalian |  |  | 7,21E-03 |
| HAS2-AS1 |  | other |  |  | 7,21E-03 |
| PRPF31 | 0,088 | other |  |  | 7,21E-03 |
| PYCR1 | -0,049 | enzyme |  |  | 7,21E-03 |
| DHODH | 0,054 | enzyme |  |  | 7,21E-03 |
| EIF3 |  | complex |  |  | 7,21E-03 |
| mapatumumab |  | biologic drug |  |  | 7,21E-03 |
| USP39 | 0,003 | peptidase |  |  | 7,21E-03 |
| allobarbital |  | chemical drug |  |  | 7,21E-03 |
| UHMK1 | -0,166 | kinase |  |  | 7,21E-03 |
| PAM | -0,005 | enzyme |  |  | 7,21E-03 |
| EIF5B | -0,012 | translation regulator |  |  | 7,21E-03 |
| LINC00570 |  | other |  |  | 7,21E-03 |
| HTRA2 | 0,041 | peptidase |  |  | 7,21E-03 |
| isorhapontigenin |  | chemical - endogenous non-mammalian |  |  | 7,21E-03 |
| aloperine |  | chemical reagent |  |  | 7,21E-03 |
| NKX2-8 | 0,100 | transcription regulator |  |  | 7,21E-03 |
| KIF11 | -0,156 | other |  |  | 7,21E-03 |
| TBC1D1 | -0,065 | other |  |  | 7,21E-03 |
| ARL6IP5 | 0,004 | other |  |  | 7,21E-03 |
| UBL5 | -0,022 | other |  |  | 7,21E-03 |
| LINC02206 |  | other |  |  | 7,21E-03 |
| kurarinone |  | chemical reagent |  |  | 7,21E-03 |
| diclofenac/hyaluronic acid |  | chemical drug |  |  | 7,21E-03 |
| annonacin |  | chemical reagent |  |  | 7,21E-03 |
| droxinostat |  | chemical reagent |  |  | 7,21E-03 |
| amurensin G |  | chemical reagent |  |  | 7,21E-03 |
| tetraarsenic tetrasulfide |  | chemical reagent |  |  | 7,21E-03 |
| bisindolylmaleimide III |  | chemical - kinase inhibitor |  |  | 7,21E-03 |
| barbital |  | chemical drug |  |  | 7,21E-03 |
| isosafrole |  | chemical toxicant |  |  | 7,21E-03 |
| STAT3 inhibitor IX |  | chemical reagent |  |  | 7,21E-03 |
| INHA | 1,207 | growth factor |  | 1,091 | 7,31E-03 |
| PI-103 |  | chemical drug |  |  | 7,32E-03 |
| miR-128-3p (and other miRNAs w/seed CACAGUG) |  | mature microRNA |  |  | 7,32E-03 |
| AKR1B1 | 0,000 | enzyme |  |  | 7,32E-03 |
| HSF1 | 0,033 | transcription regulator |  | -0,715 | 7,55E-03 |
| raloxifene |  | chemical drug |  | -1,000 | 8,25E-03 |
| MAP2K4 | 0,012 | kinase |  |  | 8,49E-03 |
| PLAG1 | -0,190 | transcription regulator |  |  | 8,49E-03 |
| dacinostat |  | chemical drug |  |  | 9,01E-03 |
| TP53COR1 |  | other |  |  | 9,01E-03 |
| TNFRSF13B |  | transmembrane receptor |  |  | 9,91E-03 |
| GF 120918 |  | chemical reagent |  |  | 9,91E-03 |
| salinosporamide A |  | chemical drug |  |  | 1,08E-02 |
| Abcb1b |  | transporter |  |  | 1,08E-02 |
| NEUROG1 |  | transcription regulator |  |  | 1,16E-02 |
| CCK | 0,000 | other |  |  | 1,18E-02 |
| FSHB |  | other |  |  | 1,18E-02 |
| evodiamine |  | chemical - endogenous non-mammalian |  |  | 1,18E-02 |
| pregna-4,17-diene-3,16-dione |  | chemical - endogenous non-mammalian |  |  | 1,28E-02 |
| zerumbone |  | chemical - endogenous non-mammalian |  |  | 1,28E-02 |
| MAP3K3 | -0,053 | kinase |  |  | 1,28E-02 |
| BMPER | -0,134 | other |  |  | 1,28E-02 |
| NUPR1 | 0,847 | transcription regulator |  | 0,333 | 1,29E-02 |
| tributyltin |  | chemical reagent |  |  | 1,35E-02 |
| Clock-Bmal1 |  | complex |  |  | 1,44E-02 |
| Gelonin |  | chemical toxicant |  |  | 1,44E-02 |
| DPTA-NONOate |  | chemical reagent |  |  | 1,44E-02 |
| Cry-Period |  | complex |  |  | 1,44E-02 |
| cytochrome-c oxidase |  | complex |  |  | 1,44E-02 |
| SPINK7 |  | other |  |  | 1,44E-02 |
| KIR |  | group |  |  | 1,44E-02 |
| PRPF8 | -0,054 | other |  |  | 1,44E-02 |
| miR-512-3p (miRNAs w/seed AGUGCUG) |  | mature microRNA |  |  | 1,44E-02 |
| miR-637 (and other miRNAs w/seed CUGGGGG) |  | mature microRNA |  |  | 1,44E-02 |
| GTF2E2 | 0,002 | transcription regulator |  |  | 1,44E-02 |
| BCAN | -0,009 | other |  |  | 1,44E-02 |
| MUC16 |  | other |  |  | 1,44E-02 |
| Dst |  | other |  |  | 1,44E-02 |
| FBXO4 | 0,066 | enzyme |  |  | 1,44E-02 |
| NAGLU | 0,105 | enzyme |  |  | 1,44E-02 |
| BOC | -0,016 | other |  |  | 1,44E-02 |
| IP6K2 | 0,137 | kinase |  |  | 1,44E-02 |
| SEPTIN4 | 0,046 | enzyme |  |  | 1,44E-02 |
| BAI |  | chemical drug |  |  | 1,44E-02 |
| malonic acid |  | chemical - endogenous mammalian |  |  | 1,44E-02 |
| vitamin K1 |  | chemical - endogenous mammalian |  |  | 1,44E-02 |
| MHY1485 |  | chemical reagent |  |  | 1,44E-02 |
| alpha-TEA |  | chemical drug |  |  | 1,44E-02 |
| HMOX1 | -0,014 | enzyme |  | -1,067 | 1,46E-02 |
| methyl-beta-cyclodextrin |  | chemical drug |  |  | 1,61E-02 |
| levothyroxine |  | chemical - endogenous mammalian |  |  | 1,70E-02 |
| 1-methyl-4-phenylpyridinium |  | chemical toxicant |  |  | 1,73E-02 |
| STAT5A | 0,008 | transcription regulator |  |  | 1,83E-02 |
| tetrodotoxin |  | chemical drug |  |  | 1,92E-02 |
| (+)-MK-801 |  | chemical drug |  |  | 1,95E-02 |
| POLG | 0,034 | enzyme |  |  | 1,97E-02 |
| heparin |  | chemical - endogenous mammalian |  |  | 2,02E-02 |
| Gsk3 |  | group |  |  | 2,02E-02 |
| CFLAR | -0,371 | other |  |  | 2,09E-02 |
| ceramide |  | chemical - endogenous mammalian |  |  | 2,09E-02 |
| fludarabine |  | chemical drug |  |  | 2,09E-02 |
| mycosporine-like amino acid |  | chemical reagent |  |  | 2,15E-02 |
| ixabepilone |  | chemical drug |  |  | 2,15E-02 |
| CK1 |  | group |  |  | 2,15E-02 |
| GALNT14 | -0,536 | enzyme |  |  | 2,15E-02 |
| IPPK | 0,070 | kinase |  |  | 2,15E-02 |
| ABCC2 | -0,264 | transporter |  |  | 2,15E-02 |
| miR-222-5p (miRNAs w/seed UCAGUAG) |  | mature microRNA |  |  | 2,15E-02 |
| ELAVL2 | 0,481 | other |  |  | 2,15E-02 |
| SIVA1 |  | other |  |  | 2,15E-02 |
| SRPK2 | -0,039 | kinase |  |  | 2,15E-02 |
| SYNPO |  | other |  |  | 2,15E-02 |
| INHBC | 1,354 | growth factor |  |  | 2,15E-02 |
| CASP8AP2 | -0,123 | transcription regulator |  |  | 2,15E-02 |
| zotiraciclib |  | chemical drug |  |  | 2,15E-02 |
| RPL37 | 0,013 | other |  |  | 2,15E-02 |
| SCT |  | other |  |  | 2,15E-02 |
| SNRNP70 | 0,107 | other |  |  | 2,15E-02 |
| tempo |  | chemical reagent |  |  | 2,15E-02 |
| chitosan |  | chemical - endogenous mammalian |  |  | 2,15E-02 |
| raffinose |  | chemical - endogenous mammalian |  |  | 2,15E-02 |
| NR3C1 | -0,064 | ligand-dependent nuclear receptor |  |  | 2,17E-02 |
| ITGAV | -0,097 | transmembrane receptor |  |  | 2,22E-02 |
| KN 93 |  | chemical drug |  |  | 2,22E-02 |
| MLXIPL | -0,159 | transcription regulator |  | 1,964 | 2,24E-02 |
| PP2/AG1879 tyrosine kinase inhibitor |  | chemical drug |  |  | 2,29E-02 |
| LARP1 | -0,085 | translation regulator |  |  | 2,29E-02 |
| romidepsin |  | biologic drug |  |  | 2,29E-02 |
| Histone h4 |  | group |  |  | 2,29E-02 |
| prostaglandin J2 |  | chemical - endogenous mammalian |  |  | 2,36E-02 |
| vitamin K3 |  | chemical drug |  |  | 2,36E-02 |
| MYCL | -0,560 | transcription regulator |  |  | 2,36E-02 |
| CREB1 | -0,119 | transcription regulator |  | 0,707 | 2,43E-02 |
| SOX17 |  | transcription regulator |  |  | 2,49E-02 |
| TP53 | -0,040 | transcription regulator |  | -0,253 | 2,52E-02 |
| miR-30c-5p (and other miRNAs w/seed GUAAACA) |  | mature microRNA |  | 1,067 | 2,55E-02 |
| CAV1 | -0,048 | transmembrane receptor |  |  | 2,55E-02 |
| Ap2 |  | group |  |  | 2,63E-02 |
| mir-221 |  | microRNA |  |  | 2,63E-02 |
| miR-92a-3p (and other miRNAs w/seed AUUGCAC) |  | mature microRNA |  |  | 2,77E-02 |
| AIM2 |  | other |  |  | 2,77E-02 |
| CGP 74514A |  | chemical drug |  |  | 2,85E-02 |
| N-acetyl-D-mannosamine |  | chemical - endogenous mammalian |  |  | 2,85E-02 |
| flavokawain A |  | chemical - endogenous non-mammalian |  |  | 2,85E-02 |
| SF3B1 | -0,034 | other |  |  | 2,85E-02 |
| CTSD | 0,053 | peptidase |  |  | 2,85E-02 |
| IBSP | -1,077 | other |  |  | 2,85E-02 |
| DTNB | -0,002 | other |  |  | 2,85E-02 |
| HAS3 | 0,312 | enzyme |  |  | 2,85E-02 |
| D-mannosamine |  | chemical - endogenous mammalian |  |  | 2,85E-02 |
| FASTK | 0,063 | kinase |  |  | 2,85E-02 |
| CTNNAL1 | -0,023 | other |  |  | 2,85E-02 |
| CDC20 | -0,114 | other |  |  | 2,85E-02 |
| CRABP2 | -0,393 | transporter |  |  | 2,85E-02 |
| 1-[2,3-bis(furan-2-yl)quinoxalin-6-yl]-3-(4-bromophenyl)urea |  | chemical reagent |  |  | 2,85E-02 |
| NBN | -0,058 | other |  |  | 2,85E-02 |
| ditiocarb |  | chemical drug |  |  | 2,85E-02 |
| salsalate |  | chemical drug |  |  | 2,85E-02 |
| rubitecan |  | chemical drug |  |  | 2,85E-02 |
| nocodazole |  | chemical reagent |  |  | 2,89E-02 |
| alvocidib |  | chemical drug |  |  | 2,92E-02 |
| actinomycin D |  | biologic drug |  | 1,400 | 2,97E-02 |
| FGF2 | -0,088 | growth factor |  | -1,029 | 3,06E-02 |
| KDM2B | -0,012 | enzyme |  |  | 3,06E-02 |
| GDF9 | 0,532 | growth factor |  |  | 3,06E-02 |
| NQO1 | -0,061 | enzyme |  |  | 3,06E-02 |
| sucrose |  | chemical - endogenous mammalian |  |  | 3,21E-02 |
| SATB1 | -0,015 | transcription regulator |  | 1,067 | 3,36E-02 |
| ENG | 0,096 | transmembrane receptor |  |  | 3,37E-02 |
| mir-25 |  | microRNA |  |  | 3,52E-02 |
| M344 |  | chemical reagent |  |  | 3,52E-02 |
| advanced glycation end product 3 |  | chemical - endogenous mammalian |  |  | 3,55E-02 |
| delta-12-prostaglandin J2 |  | chemical - endogenous mammalian |  |  | 3,55E-02 |
| DIPQUO |  | chemical reagent |  |  | 3,55E-02 |
| Hottip |  | other |  |  | 3,55E-02 |
| sulfadimethoxine |  | chemical toxicant |  |  | 3,55E-02 |
| TCF15 | 0,123 | transcription regulator |  |  | 3,55E-02 |
| NDST2 | 0,011 | enzyme |  |  | 3,55E-02 |
| IgG2b |  | complex |  |  | 3,55E-02 |
| PF-562271 |  | chemical drug |  |  | 3,55E-02 |
| RPS11 | 0,131 | other |  |  | 3,55E-02 |
| LGMN | 0,073 | peptidase |  |  | 3,55E-02 |
| ELAVL4 | 0,215 | translation regulator |  |  | 3,55E-02 |
| EDIL3 | -0,092 | other |  |  | 3,55E-02 |
| CMA1 | 1,044 | peptidase |  |  | 3,55E-02 |
| NDFIP2 | 0,003 | other |  |  | 3,55E-02 |
| STK17B | -0,075 | kinase |  |  | 3,55E-02 |
| polyinosine-polycytidylic acid/polyethylenimine formulation |  | chemical reagent |  |  | 3,55E-02 |
| pentamidine |  | chemical drug |  |  | 3,55E-02 |
| Collagen type V |  | complex |  |  | 3,55E-02 |
| Arg-Gly-Asp |  | chemical reagent |  |  | 3,55E-02 |
| CYP1A1 | -0,709 | enzyme |  |  | 3,58E-02 |
| PEBP1 | 0,072 | other |  |  | 3,68E-02 |
| fisetin |  | chemical drug |  |  | 3,68E-02 |
| RPS6KB1 | -0,075 | kinase |  |  | 3,84E-02 |
| HNF4A | -1,059 | transcription regulator |  | -0,651 | 3,92E-02 |
| ShK-223 |  | chemical reagent |  |  | 3,95E-02 |
| CSF2 | 2,963 | cytokine | Inhibited | -2,213 | 4,08E-02 |
| YAP1 | -0,074 | transcription regulator |  |  | 4,11E-02 |
| ESRRB | -1,825 | transcription regulator |  |  | 4,18E-02 |
| Rp-8-pCPT-cGMPS-triethylamine |  | chemical reagent |  |  | 4,25E-02 |
| C9orf116 |  | other |  |  | 4,25E-02 |
| AMELY |  | growth factor |  |  | 4,25E-02 |
| DPPA3 |  | other |  |  | 4,25E-02 |
| CPXM1 | -1,289 | peptidase |  |  | 4,25E-02 |
| AMER1 | -0,192 | other |  |  | 4,25E-02 |
| PIWIL4 | -0,000 | other |  |  | 4,25E-02 |
| GNB5 | -0,086 | enzyme |  |  | 4,25E-02 |
| HAS1 |  | enzyme |  |  | 4,25E-02 |
| miR-193a-5p (miRNAs w/seed GGGUCUU) |  | mature microRNA |  |  | 4,25E-02 |
| miR-320b (and other miRNAs w/seed AAAGCUG) |  | mature microRNA |  |  | 4,25E-02 |
| NUDT21 | -0,088 | other |  |  | 4,25E-02 |
| HTR4 | 0,405 | G-protein coupled receptor |  |  | 4,25E-02 |
| DSPP |  | other |  |  | 4,25E-02 |
| HIVEP2 | -0,064 | transcription regulator |  |  | 4,25E-02 |
| Gm19378 |  | other |  |  | 4,25E-02 |
| RPS20 | -0,036 | other |  |  | 4,25E-02 |
| FZD6 | -0,053 | G-protein coupled receptor |  |  | 4,25E-02 |
| LGH447 |  | chemical drug |  |  | 4,25E-02 |
| Congo Red |  | chemical toxicant |  |  | 4,25E-02 |
| acetazolamide |  | chemical drug |  |  | 4,25E-02 |
| BML-284 |  | chemical reagent |  |  | 4,25E-02 |
| Z-VRPR-FMK |  | chemical - protease inhibitor |  |  | 4,25E-02 |
| benz[a]anthracene |  | chemical toxicant |  |  | 4,25E-02 |
| catechol |  | chemical - endogenous mammalian |  |  | 4,25E-02 |
| resiniferatoxin |  | chemical drug |  |  | 4,25E-02 |
| CV 3988 |  | chemical reagent |  |  | 4,25E-02 |
| phenylamil |  | chemical reagent |  |  | 4,25E-02 |
| oxamic acid |  | chemical - endogenous non-mammalian |  |  | 4,25E-02 |
| Z-LLL-CHO |  | chemical - protease inhibitor |  | 1,000 | 4,26E-02 |
| EP300 |  | transcription regulator |  |  | 4,32E-02 |
| E. coli serotype 0127B8 lipopolysaccharide |  | chemical - endogenous non-mammalian |  | 0,000 | 4,44E-02 |
| KAT5 | 0,009 | transcription regulator |  |  | 4,54E-02 |
| CCN2 | 0,057 | growth factor |  |  | 4,54E-02 |
| OLR1 |  | transmembrane receptor |  |  | 4,69E-02 |
| SPP1 | -1,089 | cytokine |  | -1,829 | 4,89E-02 |
| flavokawain B |  | chemical - endogenous non-mammalian |  |  | 4,94E-02 |
| doramapimod |  | chemical drug |  |  | 4,94E-02 |
| menthol |  | chemical drug |  |  | 4,94E-02 |
| RIOX1 | 0,046 | enzyme |  |  | 4,94E-02 |
| SOX2-OCT4 |  | complex |  |  | 4,94E-02 |
| Collagen type III |  | complex |  |  | 4,94E-02 |
| EZH1 | 0,004 | enzyme |  |  | 4,94E-02 |
| ERCC1 | 0,028 | enzyme |  |  | 4,94E-02 |
| OGN | 2,500 | growth factor |  |  | 4,94E-02 |
| THOC1 | 0,061 | transcription regulator |  |  | 4,94E-02 |
| NPAS2 | 0,012 | transcription regulator |  |  | 4,94E-02 |
| FZD2 | -0,008 | G-protein coupled receptor |  |  | 4,94E-02 |
| MFAP2 | -0,131 | other |  |  | 4,94E-02 |
| H4C3 |  | other |  |  | 4,94E-02 |
| benzoquinone |  | chemical - endogenous mammalian |  |  | 4,94E-02 |
| AR-A014418 |  | chemical reagent |  |  | 4,94E-02 |
| trimethyltin |  | chemical reagent |  |  | 4,94E-02 |
| clonazepam |  | chemical drug |  |  | 4,94E-02 |
| DDT |  | chemical toxicant |  |  | 4,94E-02 |
| zVAD |  | chemical - protease inhibitor |  |  | 4,94E-02 |
| GRIN3A | 0,532 | ion channel |  |  | 4,95E-02 |
| U0126 |  | chemical drug |  | -0,104 | 5,00E-02 |
| IKBKB | 0,105 | kinase | Inhibited | -2,160 | 6,07E-02 |
| tazemetostat |  | chemical drug | Activated | 2,236 | 7,03E-02 |
| SP2509 |  | chemical reagent | Activated | 2,236 | 7,50E-02 |
| PD98059 |  | chemical - kinase inhibitor | Activated | 2,027 | 1,31E-01 |
| curcumin |  | chemical drug | Activated | 2,173 | 1,47E-01 |
| Immunoglobulin |  | complex |  | 1,910 | 1,63E-01 |
| MYCN | -0,097 | transcription regulator |  | 1,964 | 2,23E-01 |
| arsenic trioxide |  | chemical drug |  | 1,972 | 2,23E-01 |
| TNF | 0,867 | cytokine |  | -1,850 | 2,45E-01 |
| TCF4 | -0,192 | transcription regulator | Inhibited | -2,000 | 2,60E-01 |
| RARA | -0,002 | ligand-dependent nuclear receptor |  | 1,982 | 2,70E-01 |
| lipopolysaccharide |  | chemical drug | Inhibited | -2,503 | 1,00E00 |

| Melatonin + IIK7 treatment (10^-9^ M) | | | | | | | | | | | |
| --- | --- | --- | --- | --- | --- | --- | --- | --- | --- | --- | --- |
| Upstream Regulator | | **Expr Log Ratio** | | **Molecule Type** | | **Predicted Activation State** | | **Activation z-score** | | **p-value of overlap** | |
| ZBTB17 | | 0,032 | | transcription regulator | |  | |  | | 2,11E-26 | |
| E2F4 | | -0,076 | | transcription regulator | | Activated | | 2,000 | | 5,77E-26 | |
| l-asparaginase | |  | | biologic drug | | Activated | | 5,657 | | 3,87E-25 | |
| CKAP2L | | -0,357 | | other | | Inhibited | | -4,796 | | 1,19E-23 | |
| CDKN1A | | 0,004 | | kinase | | Activated | | 3,219 | | 6,36E-23 | |
| CEBPB | |  | | transcription regulator | | Inhibited | | -4,275 | | 3,72E-21 | |
| COPS5 | | -0,065 | | transcription regulator | |  | | -0,667 | | 1,50E-18 | |
| discodermolide | |  | | chemical drug | |  | |  | | 7,45E-18 | |
| calcitriol | |  | | chemical drug | | Activated | | 4,697 | | 1,53E-17 | |
| PTGER2 | | -0,254 | | G-protein coupled receptor | | Inhibited | | -4,487 | | 1,66E-16 | |
| RABL6 | | -0,081 | | other | | Inhibited | | -4,359 | | 7,85E-16 | |
| aflatoxin B1 | |  | | chemical - endogenous non-mammalian | | Inhibited | | -5,000 | | 1,42E-15 | |
| CCND1 | | -0,354 | | transcription regulator | | Inhibited | | -2,387 | | 1,66E-14 | |
| TP53 | | -0,084 | | transcription regulator | | Activated | | 4,700 | | 6,47E-14 | |
| dextran sulfate | |  | | chemical drug | |  | |  | | 6,52E-14 | |
| MYOD1 | | 0,000 | | transcription regulator | |  | | -0,939 | | 9,09E-14 | |
| CDK4 | | 0,004 | | kinase | |  | |  | | 9,23E-14 | |
| NUPR1 | | 0,000 | | transcription regulator | | Activated | | 4,221 | | 1,06E-13 | |
| PCLAF | | -0,545 | | other | | Inhibited | | -2,998 | | 1,67E-12 | |
| telapristone acetate | |  | | chemical drug | |  | |  | | 2,21E-12 | |
| E2F1 | | -0,389 | | transcription regulator | | Inhibited | | -2,768 | | 5,75E-12 | |
| ERBB2 | | -0,009 | | kinase | | Inhibited | | -2,779 | | 7,69E-12 | |
| FOXM1 | | -0,374 | | transcription regulator | | Inhibited | | -3,701 | | 8,07E-12 | |
| CASR | | 2,305 | | G-protein coupled receptor | | Inhibited | | -2,573 | | 1,15E-10 | |
| BNIP3L | | -0,008 | | other | | Activated | | 3,742 | | 1,16E-10 | |
| TBX2 | | -0,136 | | transcription regulator | | Inhibited | | -3,873 | | 3,20E-10 | |
| mir-21 | |  | | microRNA | | Activated | | 2,883 | | 3,37E-10 | |
| E2F3 | | 0,032 | | transcription regulator | | Inhibited | | -3,988 | | 4,10E-10 | |
| methylselenic acid | |  | | chemical reagent | |  | |  | | 5,20E-10 | |
| LIN9 | | -0,080 | | other | | Inhibited | | -2,952 | | 5,77E-10 | |
| medroxyprogesterone acetate | |  | | chemical drug | |  | | 0,546 | | 7,51E-10 | |
| CSF2 | | 0,770 | | cytokine | | Inhibited | | -4,075 | | 1,01E-09 | |
| MITF | | 0,135 | | transcription regulator | | Inhibited | | -2,921 | | 1,02E-09 | |
| LDLR | | -0,098 | | transporter | |  | |  | | 1,30E-09 | |
| fulvestrant | |  | | chemical drug | | Activated | | 2,848 | | 1,38E-09 | |
| HGF | | 0,086 | | growth factor | | Inhibited | | -3,947 | | 1,83E-09 | |
| FOXO3 | | 0,069 | | transcription regulator | |  | | 1,408 | | 1,89E-09 | |
| EP400 | | 0,068 | | other | | Inhibited | | -3,148 | | 3,09E-09 | |
| imipramine blue | |  | | chemical drug | | Activated | | 2,405 | | 3,38E-09 | |
| EWSR1 | |  | | other | | Inhibited | | -2,132 | | 4,69E-09 | |
| RRP1B | | -0,097 | | transcription regulator | |  | |  | | 8,83E-09 | |
| NR1H3 | | 0,044 | | ligand-dependent nuclear receptor | |  | |  | | 1,21E-08 | |
| RB1 | | 0,024 | | transcription regulator | | Activated | | 2,308 | | 1,28E-08 | |
| HSF1 | | 0,061 | | transcription regulator | |  | | -0,338 | | 1,67E-08 | |
| 2-(4-amino-1-isopropyl-1H-pyrazolo[3,4-d]pyrimidin-3-yl)-1H-indol-5-ol | |  | | chemical reagent | | Activated | | 3,091 | | 1,79E-08 | |
| RBL2 | | 0,137 | | other | | Activated | | 2,954 | | 2,21E-08 | |
| docetaxel | |  | | chemical drug | |  | | -0,266 | | 2,23E-08 | |
| beta-estradiol | |  | | chemical - endogenous mammalian | |  | | -1,346 | | 2,29E-08 | |
| TRPS1 | | 0,074 | | transcription regulator | | Activated | | 3,302 | | 2,83E-08 | |
| Vegf | |  | | group | | Inhibited | | -3,639 | | 6,03E-08 | |
| E2F7 | | -0,107 | | transcription regulator | |  | |  | | 7,10E-08 | |
| ethidium | |  | | chemical drug | |  | | 1,633 | | 7,10E-08 | |
| CREB1 | | 0,013 | | transcription regulator | |  | | 0,438 | | 7,47E-08 | |
| CDKN2A | |  | | transcription regulator | | Activated | | 3,138 | | 7,68E-08 | |
| KRAS | | -0,105 | | enzyme | | Inhibited | | -2,553 | | 1,39E-07 | |
| TGFB1 | | -0,081 | | growth factor | |  | | 0,831 | | 1,49E-07 | |
| gefitinib | |  | | chemical drug | |  | | 0,849 | | 1,55E-07 | |
| AREG | | -0,548 | | growth factor | | Inhibited | | -3,606 | | 1,82E-07 | |
| KDM5B | | 0,009 | | transcription regulator | | Activated | | 3,487 | | 1,86E-07 | |
| ESR1 | | -0,132 | | ligand-dependent nuclear receptor | | Inhibited | | -3,798 | | 2,03E-07 | |
| MYBL2 | | -0,589 | | transcription regulator | | Inhibited | | -2,157 | | 2,12E-07 | |
| E2F2 | | -0,375 | | transcription regulator | |  | | -1,671 | | 2,20E-07 | |
| VDR | | -0,089 | | transcription regulator | | Inhibited | | -2,548 | | 2,23E-07 | |
| E2F8 | | -0,547 | | transcription regulator | |  | |  | | 2,81E-07 | |
| S100A6 | |  | | transporter | | Inhibited | | -2,121 | | 2,94E-07 | |
| let-7 | |  | | microRNA | | Activated | | 2,978 | | 3,05E-07 | |
| E2f | |  | | group | | Inhibited | | -2,595 | | 3,46E-07 | |
| TCF3 | | -0,056 | | transcription regulator | | Activated | | 2,500 | | 3,97E-07 | |
| rhodamine 6G | |  | | chemical toxicant | |  | | 1,342 | | 4,18E-07 | |
| Irgm1 | |  | | other | | Activated | | 3,592 | | 5,80E-07 | |
| Rb | |  | | group | | Activated | | 2,157 | | 6,00E-07 | |
| YY1 | | -0,056 | | transcription regulator | |  | |  | | 6,13E-07 | |
| AGN194204 | |  | | chemical drug | |  | | -0,113 | | 6,35E-07 | |
| HOXD12 | | -1,818 | | transcription regulator | |  | | 0,762 | | 1,63E-06 | |
| NFYB | | -0,042 | | transcription regulator | |  | |  | | 2,88E-06 | |
| NSUN6 | | -0,010 | | enzyme | |  | | -0,707 | | 3,41E-06 | |
| trichostatin A | |  | | chemical drug | |  | | 0,782 | | 3,63E-06 | |
| ESR2 | | 0,175 | | ligand-dependent nuclear receptor | |  | | -1,082 | | 3,63E-06 | |
| HNF1A-AS1 | |  | | other | | Inhibited | | -2,449 | | 3,93E-06 | |
| H2AZ1 | | -0,370 | | other | | Inhibited | | -2,449 | | 4,38E-06 | |
| 8-bromo-cAMP | |  | | chemical reagent | |  | | -1,633 | | 4,59E-06 | |
| YAP1 | | -0,008 | | transcription regulator | | Inhibited | | -2,802 | | 4,63E-06 | |
| MFAP5 | | -0,109 | | other | |  | | 0,718 | | 5,14E-06 | |
| KAT2A | | 0,057 | | enzyme | | Inhibited | | -2,887 | | 8,66E-06 | |
| GW3965 | |  | | chemical reagent | | Activated | | 2,534 | | 9,46E-06 | |
| KDM3B | | -0,009 | | enzyme | | Activated | | 2,646 | | 9,57E-06 | |
| AGT | |  | | growth factor | |  | | -0,310 | | 9,94E-06 | |
| RBX1 | | 0,031 | | enzyme | |  | | 0,000 | | 1,10E-05 | |
| CKS1B | | -0,346 | | kinase | |  | | -1,981 | | 1,10E-05 | |
| GON4L | | 0,093 | | transcription regulator | |  | |  | | 1,33E-05 | |
| NDUFA13 | | 0,186 | | enzyme | |  | | 1,536 | | 1,33E-05 | |
| CDKN1B | | 0,008 | | kinase | |  | | 0,693 | | 1,34E-05 | |
| COTI-2 | |  | | chemical drug | |  | | 1,000 | | 2,15E-05 | |
| RNA polymerase II | |  | | complex | |  | |  | | 2,24E-05 | |
| TCF4 | | -0,017 | | transcription regulator | |  | | -1,667 | | 2,27E-05 | |
| doxorubicin | |  | | chemical drug | |  | | 0,966 | | 2,34E-05 | |
| SP2509 | |  | | chemical reagent | | Activated | | 2,482 | | 2,37E-05 | |
| paclitaxel | |  | | chemical drug | |  | | 0,108 | | 2,49E-05 | |
| TP73 | | -0,629 | | transcription regulator | |  | | 0,221 | | 2,52E-05 | |
| decitabine | |  | | chemical drug | |  | | 1,167 | | 2,66E-05 | |
| 4-nitroquinoline-1-oxide | |  | | chemical toxicant | | Inhibited | | -2,236 | | 2,78E-05 | |
| MYC | | -0,005 | | transcription regulator | | Inhibited | | -3,628 | | 2,98E-05 | |
| IL6 | |  | | cytokine | |  | | 0,827 | | 2,99E-05 | |
| mibolerone | |  | | chemical drug | |  | | -1,387 | | 3,49E-05 | |
| SMARCB1 | | 0,005 | | transcription regulator | | Activated | | 3,188 | | 3,52E-05 | |
| CD3 | |  | | complex | |  | | -1,641 | | 3,70E-05 | |
| bendamustine | |  | | chemical drug | |  | | 0,000 | | 3,80E-05 | |
| CDKN2B | |  | | transcription regulator | |  | | -0,152 | | 3,80E-05 | |
| etoposide | |  | | chemical drug | |  | | -0,148 | | 4,20E-05 | |
| TLX1 | | 0,671 | | transcription regulator | |  | | -1,633 | | 4,54E-05 | |
| NFYA | | 0,055 | | transcription regulator | |  | | -0,975 | | 4,56E-05 | |
| KLK4 | | 0,000 | | peptidase | |  | |  | | 5,24E-05 | |
| AR | | 0,095 | | ligand-dependent nuclear receptor | |  | | -1,427 | | 5,25E-05 | |
| CKS2 | | -0,467 | | kinase | |  | |  | | 5,45E-05 | |
| FSH | |  | | complex | |  | | -0,847 | | 5,51E-05 | |
| topotecan | |  | | chemical drug | |  | | -1,127 | | 5,66E-05 | |
| DSCAML1 | |  | | other | |  | | 0,000 | | 6,12E-05 | |
| butyric acid | |  | | chemical - endogenous mammalian | |  | | 1,884 | | 6,19E-05 | |
| HOXD9 | | 0,026 | | transcription regulator | |  | | 0,762 | | 6,21E-05 | |
| IGF2 | | 0,259 | | growth factor | |  | |  | | 6,67E-05 | |
| progesterone | |  | | chemical - endogenous mammalian | |  | | 0,937 | | 6,94E-05 | |
| PIM1 | | -0,228 | | kinase | |  | | -0,503 | | 7,12E-05 | |
| geldanamycin | |  | | chemical drug | |  | | -0,778 | | 7,47E-05 | |
| ACKR3 | | -0,002 | | G-protein coupled receptor | |  | | -0,283 | | 8,34E-05 | |
| metribolone | |  | | chemical reagent | |  | | -1,072 | | 8,46E-05 | |
| RBL1 | | -0,214 | | transcription regulator | |  | | 1,609 | | 9,56E-05 | |
| TASP1 | | -0,046 | | peptidase | |  | |  | | 9,57E-05 | |
| SMOC2 | |  | | other | | Inhibited | | -2,000 | | 9,57E-05 | |
| BMS-754807 | |  | | chemical drug | |  | | 1,067 | | 9,57E-05 | |
| trestolone | |  | | chemical drug | |  | |  | | 9,57E-05 | |
| SIX2 | | 0,026 | | transcription regulator | |  | | 1,414 | | 1,15E-04 | |
| BMS-690514 | |  | | chemical drug | |  | | 1,633 | | 1,20E-04 | |
| eltanexor | |  | | chemical drug | | Activated | | 2,449 | | 1,20E-04 | |
| E2F6 | | 0,000 | | transcription regulator | |  | | 1,732 | | 1,29E-04 | |
| seocalcitol | |  | | chemical drug | |  | | 1,471 | | 1,29E-04 | |
| (-)-gossypol | |  | | chemical drug | |  | |  | | 1,34E-04 | |
| TP63 | | 3,611 | | transcription regulator | | Inhibited | | -2,024 | | 1,40E-04 | |
| HOXD8 | | 0,025 | | transcription regulator | |  | | 0,762 | | 1,41E-04 | |
| tetrodotoxin | |  | | chemical drug | |  | | -1,342 | | 1,60E-04 | |
| dexamethasone | |  | | chemical drug | | Activated | | 2,190 | | 1,80E-04 | |
| camptothecin | |  | | chemical drug | |  | | -1,152 | | 2,00E-04 | |
| TEAD2 | | 0,183 | | transcription regulator | |  | | 1,000 | | 2,08E-04 | |
| MAPK1 | | 0,096 | | kinase | |  | | 0,447 | | 2,09E-04 | |
| diethylstilbestrol | |  | | chemical drug | |  | | -1,971 | | 2,12E-04 | |
| imatinib | |  | | chemical drug | |  | | 0,931 | | 2,14E-04 | |
| hydroxyurea | |  | | chemical drug | |  | | -0,518 | | 2,22E-04 | |
| RARA | | 0,071 | | ligand-dependent nuclear receptor | | Inhibited | | -3,286 | | 2,29E-04 | |
| EGFR | | 0,087 | | kinase | |  | | -1,174 | | 2,33E-04 | |
| FOXO1 | | -0,011 | | transcription regulator | | Inhibited | | -3,894 | | 2,40E-04 | |
| 5-fluoro-2-hydroxycinnamaldehyde | |  | | chemical reagent | |  | |  | | 2,63E-04 | |
| 5-fluoro-2-benzoyloxycinnamaldehyde | |  | | chemical reagent | |  | |  | | 2,63E-04 | |
| propyl-2-(8-(3,4-difluorobenzyl)-2',5'-dioxo-8-azaspiro[bicyclo[3.2.1] octane-3,4'-imidazolidine]-1'-yl)acetate | |  | | chemical reagent | |  | |  | | 2,63E-04 | |
| tanespimycin | |  | | chemical drug | |  | | 0,467 | | 2,73E-04 | |
| NAE1 | | -0,106 | | enzyme | |  | | -1,000 | | 2,74E-04 | |
| SRSF3 | | -0,213 | | other | |  | | -0,225 | | 2,74E-04 | |
| MRTFB | | 0,169 | | transcription regulator | |  | | 0,333 | | 2,82E-04 | |
| TMPRSS2-ERG | |  | | fusion gene/product | |  | | 0,143 | | 2,92E-04 | |
| PTEN | | -0,034 | | phosphatase | |  | | 1,671 | | 2,94E-04 | |
| IGFBP2 | | 0,072 | | other | |  | | -0,436 | | 3,36E-04 | |
| NRG1 | | -0,214 | | growth factor | |  | | -1,129 | | 3,61E-04 | |
| cardiotoxin | |  | | chemical - other | |  | | 0,000 | | 3,61E-04 | |
| ATP7B | | 0,018 | | transporter | | Activated | | 2,449 | | 3,62E-04 | |
| asiatic acid | |  | | chemical reagent | |  | |  | | 3,67E-04 | |
| nocodazole | |  | | chemical reagent | |  | | 0,384 | | 3,75E-04 | |
| benzyl isothiocyanate | |  | | chemical - endogenous non-mammalian | |  | | -0,119 | | 4,16E-04 | |
| L-methionine | |  | | chemical - endogenous mammalian | |  | |  | | 4,20E-04 | |
| CCNA2 | | -0,509 | | other | |  | |  | | 4,52E-04 | |
| tazemetostat | |  | | chemical drug | |  | | 1,563 | | 4,66E-04 | |
| epothilone B | |  | | chemical drug | |  | | 0,849 | | 4,80E-04 | |
| costunolide | |  | | chemical - endogenous non-mammalian | |  | | -1,982 | | 4,80E-04 | |
| ELAVL1 | | -0,118 | | other | | Inhibited | | -2,053 | | 4,87E-04 | |
| 5-N-ethylcarboxamido adenosine | |  | | chemical reagent | |  | | -0,777 | | 5,01E-04 | |
| troglitazone | |  | | chemical drug | |  | | -0,129 | | 5,15E-04 | |
| hydrogen peroxide | |  | | chemical - endogenous mammalian | |  | | 0,947 | | 5,16E-04 | |
| MLXIP | | -0,001 | | transcription regulator | |  | |  | | 5,78E-04 | |
| CYP24A1 | | 0,192 | | enzyme | |  | |  | | 5,78E-04 | |
| MHY1485 | |  | | chemical reagent | |  | |  | | 5,78E-04 | |
| APP | | 0,080 | | other | |  | | -0,010 | | 5,95E-04 | |
| NRAS | | -0,056 | | enzyme | |  | |  | | 5,98E-04 | |
| HDAC1 | | -0,091 | | transcription regulator | |  | | -0,788 | | 6,12E-04 | |
| NAMPT | | 0,168 | | cytokine | |  | | -0,302 | | 6,14E-04 | |
| HELLS | | -0,397 | | enzyme | |  | |  | | 6,16E-04 | |
| LLGL2 | | -0,265 | | other | |  | | -1,342 | | 6,87E-04 | |
| tyrphostin AG490 | |  | | chemical drug | |  | | 0,931 | | 6,93E-04 | |
| FLT3 | | -0,085 | | kinase | |  | |  | | 6,94E-04 | |
| NME1 | | 0,478 | | kinase | |  | |  | | 6,94E-04 | |
| eflornithine | |  | | chemical drug | |  | | 1,633 | | 6,94E-04 | |
| NELFCD | | -0,043 | | other | |  | |  | | 7,10E-04 | |
| IL5 | |  | | cytokine | |  | | -1,832 | | 7,15E-04 | |
| MXD1 | | 0,135 | | transcription regulator | | Activated | | 2,121 | | 7,37E-04 | |
| triptolide | |  | | chemical drug | |  | | -0,451 | | 7,45E-04 | |
| PDGF BB | |  | | complex | |  | | -0,828 | | 7,61E-04 | |
| PRKCD | | -0,110 | | kinase | |  | | 1,127 | | 7,65E-04 | |
| pimozide | |  | | chemical drug | |  | | -0,849 | | 7,76E-04 | |
| ATF6 | | 0,024 | | transcription regulator | |  | | -1,673 | | 8,54E-04 | |
| tetradecanoylphorbol acetate | |  | | chemical drug | |  | | -1,292 | | 8,80E-04 | |
| tamoxifen | |  | | chemical drug | | Activated | | 2,144 | | 9,06E-04 | |
| SHH | | -1,744 | | peptidase | |  | | -0,966 | | 9,27E-04 | |
| ADRA1D | | 0,911 | | G-protein coupled receptor | |  | |  | | 9,84E-04 | |
| NELFA | | 0,036 | | other | |  | |  | | 1,05E-03 | |
| NELFE | | -0,037 | | other | |  | |  | | 1,05E-03 | |
| cisplatin | |  | | chemical drug | |  | | -0,136 | | 1,05E-03 | |
| IL10 | |  | | cytokine | | Inhibited | | -3,048 | | 1,06E-03 | |
| Cdk | |  | | group | |  | | 0,000 | | 1,08E-03 | |
| DDIT3 | | 0,141 | | transcription regulator | |  | | -0,747 | | 1,08E-03 | |
| phenethyl isothiocyanate | |  | | chemical drug | |  | |  | | 1,13E-03 | |
| aspirin | |  | | chemical drug | |  | | -0,894 | | 1,25E-03 | |
| CG | |  | | complex | |  | | 0,598 | | 1,26E-03 | |
| PPARA | | 0,462 | | ligand-dependent nuclear receptor | |  | | 0,737 | | 1,29E-03 | |
| HOXD10 | | 2,167 | | transcription regulator | |  | | 0,239 | | 1,34E-03 | |
| FGF2 | | 0,063 | | growth factor | | Inhibited | | -2,057 | | 1,35E-03 | |
| palbociclib | |  | | chemical drug | |  | | 1,673 | | 1,36E-03 | |
| ADRA1A | |  | | G-protein coupled receptor | |  | |  | | 1,36E-03 | |
| SMARCE1 | | 0,001 | | transcription regulator | | Activated | | 2,000 | | 1,43E-03 | |
| BRCA1 | | -0,419 | | transcription regulator | |  | | -1,376 | | 1,44E-03 | |
| CD 437 | |  | | chemical drug | |  | | 1,941 | | 1,46E-03 | |
| O6-benzylguanine | |  | | chemical drug | |  | |  | | 1,47E-03 | |
| 2-hydroxy-1-naphthylaldehyde isonicotinoyl hydrazone | |  | | chemical reagent | |  | |  | | 1,47E-03 | |
| IGF1 | | 0,228 | | growth factor | |  | | -0,189 | | 1,50E-03 | |
| KITLG | | 0,012 | | growth factor | |  | | -0,368 | | 1,56E-03 | |
| bortezomib | |  | | chemical drug | |  | | -0,593 | | 1,56E-03 | |
| valproic acid | |  | | chemical drug | |  | | 1,817 | | 1,56E-03 | |
| NR3C1 | | -0,082 | | ligand-dependent nuclear receptor | |  | | -0,146 | | 1,62E-03 | |
| black raspberry extract | |  | | chemical drug | |  | |  | | 1,65E-03 | |
| GSTA4 | | 0,181 | | enzyme | |  | |  | | 1,71E-03 | |
| miR-153-3p (miRNAs w/seed UGCAUAG) | |  | | mature microRNA | |  | |  | | 1,71E-03 | |
| mir-153 | |  | | microRNA | |  | |  | | 1,71E-03 | |
| proTAME | |  | | chemical reagent | |  | |  | | 1,71E-03 | |
| GSK3235025 | |  | | chemical reagent | |  | |  | | 1,71E-03 | |
| 10E,12Z-octadecadienoic acid | |  | | chemical - endogenous mammalian | | Inhibited | | -3,317 | | 1,76E-03 | |
| n-nitrosomethylbenzylamine | |  | | chemical toxicant | |  | |  | | 1,80E-03 | |
| RASSF1 | |  | | other | |  | | -0,034 | | 1,82E-03 | |
| streptozocin | |  | | chemical drug | |  | | -0,224 | | 1,83E-03 | |
| alvocidib | |  | | chemical drug | |  | | 0,133 | | 1,83E-03 | |
| LMNB1 | | -0,568 | | other | |  | |  | | 1,83E-03 | |
| Calcineurin protein(s) | |  | | complex | |  | | 1,746 | | 1,86E-03 | |
| ERBB3 | | 0,508 | | kinase | |  | | -0,213 | | 1,87E-03 | |
| PDLIM2 | | 0,000 | | other | |  | | -1,463 | | 1,95E-03 | |
| 7-(4-fluorobenzylamino)-1,3,4,8-tetrahydropyrrolo[4,3,2-de]quinolin-8(1H)-one | |  | | chemical reagent | |  | |  | | 1,98E-03 | |
| hesperetin | |  | | chemical drug | |  | |  | | 1,98E-03 | |
| IKBKB | | 0,059 | | kinase | |  | | -1,528 | | 2,03E-03 | |
| estrogen | |  | | chemical drug | | Inhibited | | -2,225 | | 2,03E-03 | |
| DYRK1A | | -0,103 | | kinase | |  | | 1,131 | | 2,04E-03 | |
| MEX3A | | 0,011 | | other | | Inhibited | | -2,236 | | 2,07E-03 | |
| SN-38 | |  | | chemical drug | |  | | 0,762 | | 2,07E-03 | |
| ASCL1 | | 0,127 | | transcription regulator | | Inhibited | | -2,630 | | 2,08E-03 | |
| DSCAM | |  | | other | |  | | -0,707 | | 2,08E-03 | |
| okadaic acid | |  | | chemical toxicant | |  | | -0,547 | | 2,08E-03 | |
| tretinoin | |  | | chemical - endogenous mammalian | | Activated | | 2,052 | | 2,13E-03 | |
| miR-16-5p (and other miRNAs w/seed AGCAGCA) | |  | | mature microRNA | |  | | 0,407 | | 2,14E-03 | |
| 5-fluorouracil | |  | | chemical drug | |  | | 0,929 | | 2,14E-03 | |
| HRAS | | -0,192 | | enzyme | |  | | -1,022 | | 2,27E-03 | |
| Z-LLL-CHO | |  | | chemical - protease inhibitor | |  | | -1,097 | | 2,35E-03 | |
| Il3 | |  | | cytokine | |  | | -1,192 | | 2,35E-03 | |
| Hif1 | |  | | complex | |  | | 0,469 | | 2,35E-03 | |
| 1,4-bis[2-(3,5-dichloropyridyloxy)]benzene | |  | | chemical toxicant | | Inhibited | | -2,438 | | 2,36E-03 | |
| EPO | | -1,144 | | cytokine | |  | | -0,224 | | 2,40E-03 | |
| PBRM1 | | 0,002 | | other | |  | |  | | 2,59E-03 | |
| PINK1 | | 0,091 | | kinase | |  | |  | | 2,59E-03 | |
| LY-2510924 | |  | | biologic drug | |  | |  | | 2,59E-03 | |
| KPT-9274 | |  | | chemical drug | |  | |  | | 2,59E-03 | |
| cucurbitacin B | |  | | chemical - endogenous non-mammalian | |  | |  | | 2,59E-03 | |
| miR-291a-3p (and other miRNAs w/seed AAGUGCU) | |  | | mature microRNA | | Activated | | 2,023 | | 2,70E-03 | |
| curcumin | |  | | chemical drug | |  | | 1,465 | | 2,75E-03 | |
| E2F5 | | 0,114 | | transcription regulator | |  | |  | | 2,81E-03 | |
| PAX3 | | -0,072 | | transcription regulator | |  | |  | | 3,05E-03 | |
| DCAF1 | | 0,001 | | kinase | |  | | -0,762 | | 3,26E-03 | |
| CCNE1 | | -0,140 | | transcription regulator | |  | | -1,982 | | 3,26E-03 | |
| BAG1 | | -0,117 | | other | |  | |  | | 3,26E-03 | |
| HNF4A | | -1,168 | | transcription regulator | | Activated | | 2,212 | | 3,28E-03 | |
| Ppp2c | |  | | group | |  | |  | | 3,31E-03 | |
| FZR1 | | 0,019 | | kinase | |  | |  | | 3,31E-03 | |
| PA2G4 | | -0,156 | | transcription regulator | |  | |  | | 3,31E-03 | |
| pirinixic acid | |  | | chemical toxicant | |  | | -1,162 | | 3,35E-03 | |
| YTHDF2 | | -0,029 | | other | |  | |  | | 3,36E-03 | |
| AKR1C3 | | 1,148 | | enzyme | |  | |  | | 3,36E-03 | |
| VLDL | |  | | complex | |  | |  | | 3,36E-03 | |
| AZD7762 | |  | | chemical drug | |  | |  | | 3,36E-03 | |
| danusertib | |  | | chemical drug | |  | |  | | 3,36E-03 | |
| CHFR | | 0,002 | | enzyme | |  | |  | | 3,36E-03 | |
| G0S2 | |  | | other | |  | |  | | 3,36E-03 | |
| LSM1 | | -0,071 | | other | |  | |  | | 3,36E-03 | |
| CDC20 | | -0,743 | | other | |  | |  | | 3,36E-03 | |
| USP1 | | -0,158 | | peptidase | |  | |  | | 3,36E-03 | |
| WTAP | | -0,057 | | other | |  | |  | | 3,36E-03 | |
| abrocitinib | |  | | chemical drug | |  | |  | | 3,36E-03 | |
| karenitecin | |  | | chemical drug | |  | |  | | 3,36E-03 | |
| STUB1 | | -0,036 | | enzyme | | Activated | | 2,236 | | 3,43E-03 | |
| celecoxib | |  | | chemical drug | |  | | 0,518 | | 3,47E-03 | |
| RPS6KB1 | | -0,006 | | kinase | |  | | 1,400 | | 3,59E-03 | |
| everolimus | |  | | chemical drug | |  | | 1,067 | | 3,59E-03 | |
| STAT5a/b | |  | | group | |  | | 0,729 | | 3,66E-03 | |
| HSPB1 | | -0,027 | | other | |  | | -1,980 | | 3,75E-03 | |
| NOTCH4 | | -0,478 | | transcription regulator | |  | | -1,000 | | 3,75E-03 | |
| TPH1 | | -0,310 | | enzyme | |  | | -1,890 | | 3,86E-03 | |
| KDM1A | | -0,026 | | enzyme | | Inhibited | | -3,583 | | 4,08E-03 | |
| MYB | | 0,086 | | transcription regulator | |  | | -1,523 | | 4,18E-03 | |
| ACTL6A | | -0,020 | | other | | Inhibited | | -2,000 | | 4,30E-03 | |
| ZNF281 | | -0,032 | | transcription regulator | |  | | -1,342 | | 4,38E-03 | |
| baicalein | |  | | chemical drug | |  | | 0,301 | | 4,38E-03 | |
| SMAD7 | | 0,059 | | transcription regulator | |  | | -1,353 | | 4,40E-03 | |
| Mek | |  | | group | |  | | 0,737 | | 4,44E-03 | |
| IL15 | | 0,167 | | cytokine | |  | | 0,418 | | 4,63E-03 | |
| PDCD1 | |  | | phosphatase | |  | | 1,633 | | 4,70E-03 | |
| BRAF | | 0,160 | | kinase | |  | |  | | 4,71E-03 | |
| miR-124-3p (and other miRNAs w/seed AAGGCAC) | |  | | mature microRNA | |  | | 0,353 | | 4,75E-03 | |
| SP600125 | |  | | chemical drug | |  | | 0,108 | | 4,81E-03 | |
| trans-cinnamaldehyde | |  | | chemical drug | |  | | 1,000 | | 4,89E-03 | |
| dicarbethoxydihydrocollidine | |  | | chemical toxicant | |  | |  | | 4,89E-03 | |
| forskolin | |  | | chemical toxicant | |  | | 0,552 | | 4,95E-03 | |
| CLCN5 | | 0,199 | | ion channel | |  | |  | | 5,08E-03 | |
| PDGFRB | | 0,054 | | kinase | |  | |  | | 5,08E-03 | |
| SIAH2 | | 0,014 | | transcription regulator | |  | |  | | 5,08E-03 | |
| DEK | | -0,166 | | transcription regulator | |  | |  | | 5,08E-03 | |
| Mn(III)-tetrakis-(4-benzoic acid) porphyrin | |  | | chemical reagent | |  | |  | | 5,08E-03 | |
| EIF4E | | -0,122 | | translation regulator | |  | | -1,005 | | 5,27E-03 | |
| cytarabine | |  | | chemical drug | |  | | -1,858 | | 5,46E-03 | |
| GATA1 | | 0,017 | | transcription regulator | | Activated | | 2,028 | | 5,50E-03 | |
| CDK12 | | -0,012 | | kinase | |  | |  | | 5,51E-03 | |
| HYAL1 | |  | | enzyme | |  | |  | | 5,51E-03 | |
| TLN1 | | -0,013 | | other | |  | |  | | 5,51E-03 | |
| miR-379-5p (and other miRNAs w/seed GGUAGAC) | |  | | mature microRNA | |  | |  | | 5,51E-03 | |
| NRP2 | | -0,028 | | kinase | |  | |  | | 5,51E-03 | |
| URI1 | | -0,009 | | transcription regulator | |  | |  | | 5,51E-03 | |
| CDC27 | | -0,086 | | other | |  | |  | | 5,51E-03 | |
| SQ 29548 | |  | | chemical reagent | |  | |  | | 5,51E-03 | |
| sodium azide | |  | | chemical toxicant | |  | |  | | 5,51E-03 | |
| floxuridine | |  | | chemical drug | |  | |  | | 5,51E-03 | |
| vemurafenib | |  | | chemical drug | |  | | 0,987 | | 5,54E-03 | |
| NR4A3 | | -0,101 | | ligand-dependent nuclear receptor | |  | | -1,890 | | 5,67E-03 | |
| KLF5 | | -0,120 | | transcription regulator | |  | | -1,941 | | 5,67E-03 | |
| MET | | -0,214 | | kinase | |  | | -1,678 | | 5,72E-03 | |
| CD38 | |  | | enzyme | |  | | -1,841 | | 5,73E-03 | |
| dihydrotestosterone | |  | | chemical - endogenous mammalian | |  | | -0,344 | | 5,94E-03 | |
| CD28 | | -1,185 | | transmembrane receptor | | Inhibited | | -2,217 | | 6,04E-03 | |
| cyclic GMP | |  | | chemical - endogenous mammalian | |  | |  | | 6,15E-03 | |
| miR-143-3p (and other miRNAs w/seed GAGAUGA) | |  | | mature microRNA | |  | |  | | 6,15E-03 | |
| asciminib | |  | | chemical drug | |  | |  | | 6,15E-03 | |
| caspase | |  | | group | |  | |  | | 6,24E-03 | |
| RNF181 | | -0,099 | | enzyme | |  | | 0,000 | | 6,24E-03 | |
| MAC | |  | | complex | |  | | 0,762 | | 6,24E-03 | |
| FANCC | | -0,153 | | other | |  | |  | | 6,30E-03 | |
| MED1 | | -0,021 | | transcription regulator | | Inhibited | | -2,945 | | 6,32E-03 | |
| GRIN3A | | 0,893 | | ion channel | | Inhibited | | -2,828 | | 6,35E-03 | |
| TCF | |  | | group | |  | |  | | 6,40E-03 | |
| Akt | |  | | group | |  | | 1,537 | | 6,43E-03 | |
| SNCA | | -0,272 | | enzyme | |  | | 0,335 | | 6,60E-03 | |
| CTLA4 | | -1,158 | | transmembrane receptor | |  | | 1,633 | | 6,75E-03 | |
| ITGB3 | | -0,099 | | transmembrane receptor | |  | |  | | 6,75E-03 | |
| grape seed extract | |  | | chemical drug | |  | |  | | 7,00E-03 | |
| ITGAV | | 0,044 | | transmembrane receptor | |  | | 0,106 | | 7,00E-03 | |
| PRLR | | 1,953 | | transmembrane receptor | |  | | -1,690 | | 7,00E-03 | |
| PGR | | 0,164 | | ligand-dependent nuclear receptor | |  | | 1,494 | | 7,19E-03 | |
| ZFP36 | | -0,016 | | transcription regulator | | Activated | | 2,646 | | 7,19E-03 | |
| sirolimus | |  | | chemical drug | | Activated | | 3,202 | | 7,22E-03 | |
| MXI1 | | 0,178 | | transcription regulator | |  | |  | | 7,33E-03 | |
| ARG1 | | -0,446 | | enzyme | |  | |  | | 7,33E-03 | |
| NRP1 | | 0,091 | | transmembrane receptor | |  | |  | | 7,33E-03 | |
| mir-30 | |  | | microRNA | |  | | 0,774 | | 7,49E-03 | |
| ziritaxestat | |  | | chemical drug | |  | | 1,342 | | 7,49E-03 | |
| ADRB | |  | | group | |  | | -0,156 | | 7,53E-03 | |
| UBQLN2 | | 0,036 | | other | |  | |  | | 8,13E-03 | |
| norethindrone acetate | |  | | chemical drug | |  | |  | | 8,13E-03 | |
| HSD17B12 | | -0,022 | | enzyme | |  | |  | | 8,13E-03 | |
| ACOT13 | |  | | enzyme | |  | |  | | 8,13E-03 | |
| MAP4K1 | | -0,213 | | kinase | |  | |  | | 8,13E-03 | |
| HAS1 | |  | | enzyme | |  | |  | | 8,13E-03 | |
| LRIG1 | | -0,075 | | other | |  | |  | | 8,13E-03 | |
| TFF2 | |  | | other | |  | |  | | 8,13E-03 | |
| fenebrutinib | |  | | chemical drug | |  | |  | | 8,13E-03 | |
| pyridoxamine | |  | | chemical - endogenous mammalian | |  | |  | | 8,13E-03 | |
| zonisamide | |  | | chemical drug | |  | |  | | 8,13E-03 | |
| Z-VEID-FMK | |  | | chemical reagent | |  | |  | | 8,13E-03 | |
| ATM | | 0,048 | | kinase | |  | |  | | 8,26E-03 | |
| azoxymethane | |  | | chemical toxicant | |  | |  | | 8,26E-03 | |
| NFKBIA | | -0,004 | | transcription regulator | |  | | -0,343 | | 8,60E-03 | |
| TAL1 | | 0,722 | | transcription regulator | |  | | -1,890 | | 8,63E-03 | |
| TAF1 | | 0,078 | | transcription regulator | |  | |  | | 8,64E-03 | |
| sertraline | |  | | chemical drug | |  | |  | | 8,64E-03 | |
| LOC105372576 | |  | | other | |  | | -1,961 | | 8,69E-03 | |
| TAZ | | -1,576 | | enzyme | |  | | -1,195 | | 8,97E-03 | |
| E. coli B4 lipopolysaccharide | |  | | chemical toxicant | | Inhibited | | -2,028 | | 9,29E-03 | |
| PAX3-FOXO1 | |  | | fusion gene/product | |  | | 1,554 | | 9,29E-03 | |
| bee venom | |  | | chemical - endogenous non-mammalian | | Activated | | 2,449 | | 9,48E-03 | |
| RHOA | | -0,058 | | enzyme | |  | | -1,408 | | 9,48E-03 | |
| INSR | | -0,092 | | kinase | |  | | 0,378 | | 9,50E-03 | |
| trabectedin | |  | | chemical drug | |  | |  | | 9,63E-03 | |
| NUMB | | -0,048 | | other | |  | |  | | 1,01E-02 | |
| APOC1 | |  | | transporter | |  | |  | | 1,01E-02 | |
| antimycin A | |  | | chemical - endogenous non-mammalian | |  | |  | | 1,01E-02 | |
| acetyl-L-carnitine | |  | | chemical - endogenous mammalian | |  | |  | | 1,01E-02 | |
| TSHZ3 | | -0,180 | | transcription regulator | |  | | 0,314 | | 1,03E-02 | |
| iron | |  | | chemical - endogenous mammalian | |  | | -0,362 | | 1,03E-02 | |
| OSM | |  | | cytokine | |  | | 0,601 | | 1,05E-02 | |
| PIAS1 | | 0,009 | | transcription regulator | |  | | -0,816 | | 1,06E-02 | |
| ursolic acid | |  | | chemical drug | |  | | -0,152 | | 1,06E-02 | |
| TFDP1 | | -0,110 | | transcription regulator | |  | |  | | 1,06E-02 | |
| miR-450a-5p (and other miRNAs w/seed UUUGCGA) | |  | | mature microRNA | |  | | 0,000 | | 1,06E-02 | |
| FUS-DDIT3 | |  | | fusion gene/product | |  | | 0,000 | | 1,06E-02 | |
| MIR17HG | |  | | other | |  | | -1,414 | | 1,08E-02 | |
| GNE-495 | |  | | chemical drug | |  | |  | | 1,12E-02 | |
| TP73-AS1 | |  | | other | |  | |  | | 1,12E-02 | |
| APC (complex) | |  | | complex | |  | |  | | 1,12E-02 | |
| PCM1 | | -0,010 | | other | |  | |  | | 1,12E-02 | |
| IER3 | | -0,595 | | other | |  | |  | | 1,12E-02 | |
| CR1 | |  | | transmembrane receptor | |  | |  | | 1,12E-02 | |
| STIM2 | | -0,046 | | transporter | |  | |  | | 1,12E-02 | |
| mir-374 | |  | | microRNA | |  | |  | | 1,12E-02 | |
| E4F1 | | 0,024 | | transcription regulator | |  | |  | | 1,12E-02 | |
| 4-coumaric acid | |  | | chemical - endogenous mammalian | |  | |  | | 1,12E-02 | |
| miR-124 mimic | |  | | chemical reagent | |  | |  | | 1,12E-02 | |
| leucovorin | |  | | chemical - endogenous mammalian | |  | |  | | 1,12E-02 | |
| PRKCE | | 0,117 | | kinase | |  | | 0,323 | | 1,13E-02 | |
| GLI1 | | -0,024 | | transcription regulator | |  | | -1,024 | | 1,13E-02 | |
| 26s Proteasome | |  | | complex | |  | | -0,993 | | 1,15E-02 | |
| ERVW-1 | |  | | other | |  | |  | | 1,17E-02 | |
| CHEK1 | | -0,140 | | kinase | |  | |  | | 1,17E-02 | |
| miR-296-5p (miRNAs w/seed GGGCCCC) | |  | | mature microRNA | |  | |  | | 1,17E-02 | |
| IAPP | |  | | other | |  | |  | | 1,17E-02 | |
| HOXD3 | | 0,136 | | transcription regulator | |  | |  | | 1,17E-02 | |
| RPA1 | | -0,087 | | other | |  | |  | | 1,17E-02 | |
| HNRNPAB | | -0,108 | | enzyme | |  | |  | | 1,17E-02 | |
| TXNIP | | 0,143 | | other | |  | | 1,000 | | 1,19E-02 | |
| rifampin | |  | | chemical drug | |  | |  | | 1,20E-02 | |
| 2-deoxyglucose | |  | | chemical drug | |  | | 0,024 | | 1,20E-02 | |
| Nfat (family) | |  | | group | |  | | 1,890 | | 1,23E-02 | |
| LY294002 | |  | | chemical drug | |  | | 1,823 | | 1,25E-02 | |
| trans-hydroxytamoxifen | |  | | chemical drug | |  | | -0,632 | | 1,25E-02 | |
| bleomycin | |  | | biologic drug | |  | | 0,213 | | 1,27E-02 | |
| EBI3 | | 0,932 | | cytokine | |  | | -1,501 | | 1,27E-02 | |
| eprenetapopt | |  | | chemical drug | |  | |  | | 1,28E-02 | |
| idarubicin | |  | | chemical drug | |  | |  | | 1,34E-02 | |
| linalool | |  | | chemical - endogenous non-mammalian | |  | |  | | 1,34E-02 | |
| ADCYAP1 | | -0,286 | | other | |  | | 1,212 | | 1,35E-02 | |
| fenofibrate | |  | | chemical drug | |  | | 1,200 | | 1,35E-02 | |
| NSD2 | | -0,171 | | enzyme | |  | |  | | 1,40E-02 | |
| Firre | |  | | other | |  | | -1,633 | | 1,42E-02 | |
| PIN1 | | -0,118 | | enzyme | |  | |  | | 1,42E-02 | |
| epinephrine | |  | | chemical - endogenous mammalian | |  | |  | | 1,42E-02 | |
| TEAD4 | | -0,116 | | transcription regulator | |  | | -0,128 | | 1,42E-02 | |
| S-nitroso-N-acetyl-DL-penicillamine | |  | | chemical reagent | |  | | 0,447 | | 1,46E-02 | |
| GIP | |  | | other | |  | | 0,456 | | 1,46E-02 | |
| NOTCH3 | | 0,114 | | transcription regulator | |  | | 0,254 | | 1,46E-02 | |
| ACSL4 | | 0,027 | | enzyme | |  | | -1,387 | | 1,46E-02 | |
| tetrachlorodibenzodioxin | |  | | chemical toxicant | |  | | 0,502 | | 1,46E-02 | |
| TNFSF11 | |  | | cytokine | |  | | -0,169 | | 1,47E-02 | |
| RAS | |  | | group | |  | | -1,172 | | 1,47E-02 | |
| CHCHD5 | | -0,071 | | other | |  | |  | | 1,47E-02 | |
| TRPC4AP | | 0,002 | | transporter | |  | |  | | 1,47E-02 | |
| SULT2B1 | |  | | enzyme | |  | |  | | 1,47E-02 | |
| LGI1 | | 0,000 | | other | |  | |  | | 1,47E-02 | |
| GRK5 | | -0,186 | | kinase | |  | |  | | 1,47E-02 | |
| miR-217-5p (and other miRNAs w/seed ACUGCAU) | |  | | mature microRNA | |  | |  | | 1,47E-02 | |
| miR-532-3p (miRNAs w/seed CUCCCAC) | |  | | mature microRNA | |  | |  | | 1,47E-02 | |
| miR-139-5p (miRNAs w/seed CUACAGU) | |  | | mature microRNA | |  | |  | | 1,47E-02 | |
| SOX18 | | -2,763 | | transcription regulator | |  | |  | | 1,47E-02 | |
| POU3F3 | |  | | transcription regulator | |  | |  | | 1,47E-02 | |
| LPA | |  | | other | |  | |  | | 1,47E-02 | |
| MAD2L1 | |  | | other | |  | |  | | 1,47E-02 | |
| SUPT5H | | 0,017 | | transcription regulator | |  | |  | | 1,47E-02 | |
| RASSF10 | | -0,707 | | other | |  | |  | | 1,47E-02 | |
| HSPA8 | | -0,076 | | enzyme | |  | |  | | 1,47E-02 | |
| magnesium sulfate | |  | | chemical drug | |  | |  | | 1,47E-02 | |
| citric acid | |  | | chemical - endogenous mammalian | |  | |  | | 1,47E-02 | |
| alpha-tocopherol succinate | |  | | chemical drug | |  | |  | | 1,47E-02 | |
| sivelestat | |  | | chemical drug | |  | |  | | 1,47E-02 | |
| zinc | |  | | chemical drug | |  | | 0,393 | | 1,50E-02 | |
| branched chain amino acids | |  | | chemical drug | |  | |  | | 1,52E-02 | |
| MAPK13 | | -0,059 | | kinase | |  | |  | | 1,52E-02 | |
| GADD45A | | -0,194 | | other | |  | |  | | 1,52E-02 | |
| aphidicolin | |  | | chemical toxicant | |  | |  | | 1,52E-02 | |
| probucol | |  | | chemical drug | |  | |  | | 1,52E-02 | |
| agmatine | |  | | chemical - endogenous mammalian | |  | |  | | 1,52E-02 | |
| HTT | | 0,046 | | transcription regulator | |  | |  | | 1,52E-02 | |
| WNT1 | | 0,743 | | cytokine | |  | | 0,610 | | 1,53E-02 | |
| CD24 | |  | | other | | Inhibited | | -2,646 | | 1,56E-02 | |
| nitric oxide | |  | | chemical - endogenous mammalian | |  | | 0,168 | | 1,56E-02 | |
| TRAF2 | | -0,231 | | enzyme | |  | | -1,039 | | 1,58E-02 | |
| GnRH analog | |  | | biologic drug | |  | | 0,775 | | 1,60E-02 | |
| RBPJ | | -0,131 | | transcription regulator | |  | | -0,128 | | 1,62E-02 | |
| DNMT3A | | 0,027 | | enzyme | |  | | 0,308 | | 1,65E-02 | |
| BCR (complex) | |  | | complex | |  | | 1,423 | | 1,66E-02 | |
| mir-25 | |  | | microRNA | |  | | 0,152 | | 1,66E-02 | |
| RCE1 | | -0,065 | | peptidase | |  | |  | | 1,66E-02 | |
| RETN | |  | | other | |  | | -0,371 | | 1,67E-02 | |
| mir-15 | |  | | microRNA | |  | | 0,105 | | 1,67E-02 | |
| MYCL | | -0,551 | | transcription regulator | |  | | -1,455 | | 1,67E-02 | |
| mycophenolic acid | |  | | chemical drug | |  | | -0,816 | | 1,67E-02 | |
| ST1926 | |  | | chemical drug | | Activated | | 3,000 | | 1,68E-02 | |
| MTOR | | 0,056 | | kinase | |  | | 0,020 | | 1,69E-02 | |
| phytohemagglutinin | |  | | chemical drug | |  | | 0,000 | | 1,69E-02 | |
| soy isoflavones | |  | | chemical drug | |  | |  | | 1,72E-02 | |
| EIF4G1 | | -0,044 | | translation regulator | |  | |  | | 1,72E-02 | |
| GAS5 | |  | | other | |  | |  | | 1,72E-02 | |
| ACTB | | -0,163 | | other | |  | |  | | 1,72E-02 | |
| tunicamycin | |  | | chemical - endogenous non-mammalian | |  | | 0,881 | | 1,73E-02 | |
| HDAC5 | | 0,206 | | transcription regulator | |  | | 1,038 | | 1,76E-02 | |
| POU2AF1 | |  | | transcription regulator | |  | |  | | 1,76E-02 | |
| poly rI:rC-RNA | |  | | biologic drug | | Inhibited | | -2,233 | | 1,80E-02 | |
| KDM3A | | 0,103 | | transcription regulator | |  | | -0,927 | | 1,80E-02 | |
| sildenafil | |  | | chemical drug | |  | | 0,218 | | 1,80E-02 | |
| sorafenib | |  | | chemical drug | |  | | -0,094 | | 1,80E-02 | |
| FEV | | -0,363 | | transcription regulator | |  | | -0,816 | | 1,85E-02 | |
| bazedoxifene | |  | | chemical drug | |  | |  | | 1,86E-02 | |
| SERPINB2 | | 0,110 | | other | |  | |  | | 1,86E-02 | |
| BCL11A | |  | | transcription regulator | |  | |  | | 1,86E-02 | |
| SHOX | | 0,198 | | transcription regulator | |  | |  | | 1,86E-02 | |
| SHANK3 | | 0,260 | | other | |  | |  | | 1,86E-02 | |
| TKT | | -0,114 | | enzyme | |  | |  | | 1,86E-02 | |
| raltitrexed | |  | | chemical drug | |  | |  | | 1,86E-02 | |
| brimonidine | |  | | chemical drug | |  | |  | | 1,86E-02 | |
| PD 168393 | |  | | chemical drug | |  | |  | | 1,86E-02 | |
| prodigiosin | |  | | chemical toxicant | |  | |  | | 1,86E-02 | |
| D-thioctic acid | |  | | chemical - endogenous mammalian | |  | |  | | 1,86E-02 | |
| MI-503 | |  | | chemical reagent | |  | |  | | 1,86E-02 | |
| ATF3 | | -0,323 | | transcription regulator | |  | | 0,816 | | 1,87E-02 | |
| INHBA | | -0,151 | | growth factor | |  | | 1,211 | | 1,88E-02 | |
| napabucasin | |  | | chemical drug | |  | | 1,387 | | 1,89E-02 | |
| IL1B | |  | | cytokine | |  | | 0,454 | | 1,89E-02 | |
| FBN1 | | 0,272 | | other | |  | |  | | 1,93E-02 | |
| DRAP1 | | -0,201 | | transcription regulator | |  | |  | | 1,93E-02 | |
| PRKAG3 | |  | | kinase | |  | |  | | 1,94E-02 | |
| MYOC | |  | | other | |  | |  | | 1,95E-02 | |
| SRSF2 | | -0,165 | | transcription regulator | |  | |  | | 1,95E-02 | |
| CSF3 | |  | | cytokine | |  | | 1,425 | | 1,97E-02 | |
| NEUROG1 | |  | | transcription regulator | |  | | 0,000 | | 2,01E-02 | |
| carbon monoxide | |  | | chemical - endogenous mammalian | |  | | 1,407 | | 2,01E-02 | |
| sulindac | |  | | chemical drug | |  | |  | | 2,01E-02 | |
| EOMES | | 0,600 | | transcription regulator | |  | | 0,478 | | 2,04E-02 | |
| COL18A1 | |  | | other | |  | | -1,664 | | 2,05E-02 | |
| TFRC | | 0,147 | | transporter | |  | |  | | 2,05E-02 | |
| NEDD9 | | 0,123 | | other | |  | | 0,000 | | 2,05E-02 | |
| ritonavir | |  | | chemical drug | |  | |  | | 2,10E-02 | |
| SKIL | | 0,054 | | transcription regulator | |  | | -1,015 | | 2,11E-02 | |
| CYP27B1 | | 0,595 | | enzyme | |  | |  | | 2,11E-02 | |
| pembrolizumab | |  | | biologic drug | |  | | 1,000 | | 2,11E-02 | |
| methyl methanesulfonate | |  | | chemical toxicant | |  | | 0,776 | | 2,11E-02 | |
| oxaliplatin | |  | | chemical drug | |  | | -1,491 | | 2,13E-02 | |
| EBF2 | | -0,136 | | transcription regulator | |  | |  | | 2,15E-02 | |
| BCL2L11 | |  | | other | |  | |  | | 2,15E-02 | |
| SOX6 | | -1,242 | | transcription regulator | |  | |  | | 2,15E-02 | |
| PCYT1A | | 0,123 | | enzyme | |  | |  | | 2,15E-02 | |
| ochratoxin A | |  | | chemical toxicant | |  | |  | | 2,15E-02 | |
| UCN-01 | |  | | chemical drug | |  | |  | | 2,15E-02 | |
| estrogen receptor | |  | | group | |  | | 1,744 | | 2,18E-02 | |
| PRKAA1 | | 0,026 | | kinase | |  | | 1,539 | | 2,22E-02 | |
| RBM5 | | 0,047 | | other | |  | | 1,154 | | 2,27E-02 | |
| TEAD | |  | | group | |  | |  | | 2,29E-02 | |
| niclosamide | |  | | chemical drug | |  | |  | | 2,29E-02 | |
| Shc | |  | | group | |  | |  | | 2,29E-02 | |
| nirogacestat | |  | | chemical drug | |  | |  | | 2,29E-02 | |
| mir-503 | |  | | microRNA | |  | |  | | 2,29E-02 | |
| alpha-santalol | |  | | chemical reagent | |  | |  | | 2,29E-02 | |
| ATF5 | | 0,020 | | transcription regulator | |  | |  | | 2,29E-02 | |
| gossypol | |  | | chemical drug | |  | |  | | 2,29E-02 | |
| palmitoylethanolamide | |  | | chemical drug | |  | |  | | 2,29E-02 | |
| thymidine | |  | | chemical - endogenous mammalian | |  | |  | | 2,29E-02 | |
| quercetin | |  | | chemical drug | |  | | 0,508 | | 2,34E-02 | |
| EP300 | |  | | transcription regulator | |  | | 0,367 | | 2,36E-02 | |
| raloxifene | |  | | chemical drug | |  | | -1,387 | | 2,38E-02 | |
| androgen | |  | | chemical drug | |  | | 1,000 | | 2,38E-02 | |
| NR2F2 | | -0,264 | | ligand-dependent nuclear receptor | |  | | -1,501 | | 2,39E-02 | |
| Trp53cor1 | |  | | other | |  | |  | | 2,39E-02 | |
| L 663536 | |  | | chemical reagent | |  | |  | | 2,39E-02 | |
| 9-hydroxy-(S)-10,12-octadecadienoic acid | |  | | chemical - endogenous mammalian | |  | |  | | 2,41E-02 | |
| Katp Channel (family) | |  | | group | |  | |  | | 2,41E-02 | |
| 2'-adenylic acid | |  | | chemical - endogenous mammalian | |  | |  | | 2,41E-02 | |
| NKX1-2 | |  | | transcription regulator | |  | |  | | 2,41E-02 | |
| omigapil | |  | | chemical drug | |  | |  | | 2,41E-02 | |
| ORG 34517 | |  | | chemical drug | |  | |  | | 2,41E-02 | |
| ZYG11B | | 0,030 | | other | |  | |  | | 2,41E-02 | |
| Hdac1/2 | |  | | group | |  | |  | | 2,41E-02 | |
| AMG-9810 | |  | | chemical reagent | |  | |  | | 2,41E-02 | |
| sodium thiosulfate | |  | | chemical drug | |  | |  | | 2,41E-02 | |
| MPHOSPH8 | | 0,033 | | transcription regulator | |  | |  | | 2,41E-02 | |
| Ces1b/Ces1c | |  | | enzyme | |  | |  | | 2,41E-02 | |
| OXSM | | 0,000 | | kinase | |  | |  | | 2,41E-02 | |
| Tug1 | |  | | other | |  | |  | | 2,41E-02 | |
| MLC1 | |  | | transporter | |  | |  | | 2,41E-02 | |
| USP49 | | 0,024 | | peptidase | |  | |  | | 2,41E-02 | |
| FBXL8 | | -1,170 | | other | |  | |  | | 2,41E-02 | |
| RGN | | 0,254 | | enzyme | |  | |  | | 2,41E-02 | |
| CYTH2 | | 0,008 | | other | |  | |  | | 2,41E-02 | |
| ADTRP | |  | | enzyme | |  | |  | | 2,41E-02 | |
| INTS12 | | -0,026 | | other | |  | |  | | 2,41E-02 | |
| CCDC71L | |  | | other | |  | |  | | 2,41E-02 | |
| N-arachidonylglycine | |  | | chemical - endogenous mammalian | |  | |  | | 2,41E-02 | |
| TMPRSS2 | |  | | peptidase | |  | |  | | 2,41E-02 | |
| UBN1 | | -0,039 | | transcription regulator | |  | |  | | 2,41E-02 | |
| THAP11 | | 0,026 | | transcription regulator | |  | |  | | 2,41E-02 | |
| IPCEF1 | | 1,248 | | enzyme | |  | |  | | 2,41E-02 | |
| TRIM17 | | -0,892 | | enzyme | |  | |  | | 2,41E-02 | |
| CLEC16A | | 0,005 | | other | |  | |  | | 2,41E-02 | |
| UBE3D | | 0,087 | | enzyme | |  | |  | | 2,41E-02 | |
| RAB35 | | -0,078 | | enzyme | |  | |  | | 2,41E-02 | |
| METAP2 | | -0,167 | | peptidase | |  | |  | | 2,41E-02 | |
| SMCHD1 | | -0,040 | | enzyme | |  | |  | | 2,41E-02 | |
| ZYG11A | | 0,589 | | other | |  | |  | | 2,41E-02 | |
| amuvatinib | |  | | chemical drug | |  | |  | | 2,41E-02 | |
| black cohosh extract | |  | | chemical drug | |  | |  | | 2,41E-02 | |
| PMF1/PMF1-BGLAP | |  | | transcription regulator | |  | |  | | 2,41E-02 | |
| voxtalisib | |  | | chemical drug | |  | |  | | 2,41E-02 | |
| PRCC | | -0,036 | | other | |  | |  | | 2,41E-02 | |
| MEDI-547 | |  | | biologic drug | |  | |  | | 2,41E-02 | |
| DYNLRB1 | | -0,041 | | other | |  | |  | | 2,41E-02 | |
| GPSM1 | | -0,045 | | other | |  | |  | | 2,41E-02 | |
| OSTN | | 0,000 | | other | |  | |  | | 2,41E-02 | |
| NAPA | | 0,033 | | transporter | |  | |  | | 2,41E-02 | |
| MRGBP | | -0,082 | | other | |  | |  | | 2,41E-02 | |
| LINC00920 | |  | | other | |  | |  | | 2,41E-02 | |
| SINHCAF | | -0,080 | | other | |  | |  | | 2,41E-02 | |
| ENAH | |  | | other | |  | |  | | 2,41E-02 | |
| CCZ1/CCZ1B | |  | | other | |  | |  | | 2,41E-02 | |
| MOS | |  | | kinase | |  | |  | | 2,41E-02 | |
| mir-617 | |  | | microRNA | |  | |  | | 2,41E-02 | |
| mir-654 | |  | | microRNA | |  | |  | | 2,41E-02 | |
| DYNLT3 | | 0,099 | | other | |  | |  | | 2,41E-02 | |
| CBLL1 | | 0,056 | | enzyme | |  | |  | | 2,41E-02 | |
| KCNA1 | | 2,026 | | ion channel | |  | |  | | 2,41E-02 | |
| AURKAIP1 | |  | | enzyme | |  | |  | | 2,41E-02 | |
| PDE1A | | 0,185 | | enzyme | |  | |  | | 2,41E-02 | |
| venetoclax | |  | | chemical drug | |  | |  | | 2,41E-02 | |
| WBP1 | | 0,065 | | other | |  | |  | | 2,41E-02 | |
| DHX29 | | -0,004 | | enzyme | |  | |  | | 2,41E-02 | |
| GNG14 | |  | | other | |  | |  | | 2,41E-02 | |
| CAB39 | | -0,022 | | enzyme | |  | |  | | 2,41E-02 | |
| INTS6 | | 0,057 | | enzyme | |  | |  | | 2,41E-02 | |
| NCBP2 | | 0,114 | | other | |  | |  | | 2,41E-02 | |
| CEP350 | | 0,105 | | other | |  | |  | | 2,41E-02 | |
| RIT1 | | -0,024 | | enzyme | |  | |  | | 2,41E-02 | |
| MPT0B214 | |  | | chemical reagent | |  | |  | | 2,41E-02 | |
| PDS5A | | 0,023 | | other | |  | |  | | 2,41E-02 | |
| RPS9 | | -0,063 | | translation regulator | |  | |  | | 2,41E-02 | |
| RARRES1 | |  | | other | |  | |  | | 2,41E-02 | |
| GRID2 | | 0,109 | | ion channel | |  | |  | | 2,41E-02 | |
| GDF10 | | 0,000 | | growth factor | |  | |  | | 2,41E-02 | |
| SMAD9 | | 0,295 | | transcription regulator | |  | |  | | 2,41E-02 | |
| GTx-560 | |  | | chemical reagent | |  | |  | | 2,41E-02 | |
| TPD52 | | 0,008 | | other | |  | |  | | 2,41E-02 | |
| PACRG | |  | | other | |  | |  | | 2,41E-02 | |
| SPAG6 | | 1,556 | | other | |  | |  | | 2,41E-02 | |
| UBAP2L | | 0,000 | | other | |  | |  | | 2,41E-02 | |
| HMGN5 | | 0,174 | | transcription regulator | |  | |  | | 2,41E-02 | |
| MPT0E028 | |  | | chemical drug | |  | |  | | 2,41E-02 | |
| MEAF6 | | 0,005 | | other | |  | |  | | 2,41E-02 | |
| spliceostatin A | |  | | chemical reagent | |  | |  | | 2,41E-02 | |
| oligomycin A | |  | | chemical - endogenous non-mammalian | |  | |  | | 2,41E-02 | |
| 3,4-(methylenedioxy)cinnamic acid | |  | | chemical reagent | |  | |  | | 2,41E-02 | |
| BMS-863233 | |  | | chemical drug | |  | |  | | 2,41E-02 | |
| NSC117079 | |  | | chemical reagent | |  | |  | | 2,41E-02 | |
| RB4 | |  | | chemical reagent | |  | |  | | 2,41E-02 | |
| RB3 | |  | | chemical reagent | |  | |  | | 2,41E-02 | |
| annonacin | |  | | chemical reagent | |  | |  | | 2,41E-02 | |
| arsenic trichloride | |  | | chemical reagent | |  | |  | | 2,41E-02 | |
| meayamycin B | |  | | chemical reagent | |  | |  | | 2,41E-02 | |
| BCL201 | |  | | chemical drug | |  | |  | | 2,41E-02 | |
| SU 9516 | |  | | chemical drug | |  | |  | | 2,41E-02 | |
| dimethyl sulfate | |  | | chemical toxicant | |  | |  | | 2,41E-02 | |
| 2-amino-2-norbornanecarboxylic acid | |  | | chemical toxicant | |  | |  | | 2,41E-02 | |
| ralaniten | |  | | chemical reagent | |  | |  | | 2,41E-02 | |
| trichloroacetic acid | |  | | chemical drug | |  | |  | | 2,41E-02 | |
| melarsoprol | |  | | chemical drug | |  | |  | | 2,41E-02 | |
| pegademase bovine | |  | | biologic drug | |  | |  | | 2,41E-02 | |
| MnTM-2-PyP | |  | | chemical reagent | |  | |  | | 2,41E-02 | |
| terameprocol | |  | | chemical drug | |  | |  | | 2,41E-02 | |
| FUS-ERG | |  | | fusion gene/product | |  | |  | | 2,41E-02 | |
| EWSR1-ERG | |  | | fusion gene/product | |  | |  | | 2,41E-02 | |
| FGFR3-TACC3 | |  | | fusion gene/product | |  | |  | | 2,41E-02 | |
| AuNP@PEG@e14a2 | |  | | chemical reagent | |  | |  | | 2,41E-02 | |
| miR-124 inhibitor | |  | | chemical reagent | |  | |  | | 2,41E-02 | |
| miR-454-3p inhibitor | |  | | chemical reagent | |  | |  | | 2,41E-02 | |
| miR-454-3p mimic | |  | | chemical reagent | |  | |  | | 2,41E-02 | |
| lometrexol | |  | | chemical drug | |  | |  | | 2,41E-02 | |
| cevimeline | |  | | chemical drug | |  | |  | | 2,41E-02 | |
| trimetrexate | |  | | chemical drug | |  | |  | | 2,41E-02 | |
| SMAD3-EP300 | |  | | complex | |  | |  | | 2,41E-02 | |
| pristanic acid | |  | | chemical - endogenous mammalian | |  | |  | | 2,41E-02 | |
| 2-ethylhexanoic acid | |  | | chemical toxicant | |  | |  | | 2,41E-02 | |
| 3'-adenylic acid | |  | | chemical - endogenous mammalian | |  | |  | | 2,41E-02 | |
| pregabalin | |  | | chemical drug | |  | |  | | 2,41E-02 | |
| miR-10a inhibitor | |  | | chemical reagent | |  | |  | | 2,41E-02 | |
| allose | |  | | chemical - endogenous mammalian | |  | |  | | 2,41E-02 | |
| 6alpha-fluorotestosterone | |  | | chemical toxicant | |  | |  | | 2,41E-02 | |
| Reverse Transcriptase | |  | | complex | |  | |  | | 2,41E-02 | |
| temozolomide | |  | | chemical drug | |  | | 1,076 | | 2,44E-02 | |
| ATF2 | | 0,084 | | transcription regulator | |  | |  | | 2,48E-02 | |
| MAPK14 | | 0,012 | | kinase | |  | | 0,262 | | 2,49E-02 | |
| MAPT | | 0,632 | | other | |  | |  | | 2,49E-02 | |
| prexasertib | |  | | chemical drug | |  | | 0,447 | | 2,53E-02 | |
| caffeine | |  | | chemical drug | |  | | 1,199 | | 2,53E-02 | |
| MAX | | -0,046 | | transcription regulator | |  | |  | | 2,60E-02 | |
| CCL2 | | -0,210 | | cytokine | |  | | 0,816 | | 2,60E-02 | |
| NR3C2 | | 0,445 | | ligand-dependent nuclear receptor | | Activated | | 2,408 | | 2,61E-02 | |
| MASTL | | -0,239 | | kinase | |  | |  | | 2,62E-02 | |
| HDAC6 | | 0,050 | | transcription regulator | |  | | 1,067 | | 2,62E-02 | |
| KDR | | -0,080 | | kinase | |  | |  | | 2,62E-02 | |
| 4-hydroxynonenal | |  | | chemical toxicant | |  | | 0,887 | | 2,62E-02 | |
| mir-26 | |  | | microRNA | |  | |  | | 2,64E-02 | |
| LTBP1 | | 0,094 | | other | |  | |  | | 2,64E-02 | |
| HDAC9 | | 0,230 | | transcription regulator | |  | |  | | 2,64E-02 | |
| DTX1 | | 0,498 | | transcription regulator | |  | |  | | 2,64E-02 | |
| GW7647 | |  | | chemical drug | |  | |  | | 2,64E-02 | |
| glutathione | |  | | chemical - endogenous mammalian | |  | | 0,424 | | 2,67E-02 | |
| PD98059 | |  | | chemical - kinase inhibitor | |  | | 0,084 | | 2,70E-02 | |
| salmonella minnesota R595 lipopolysaccharides | |  | | chemical - endogenous non-mammalian | |  | | -0,324 | | 2,72E-02 | |
| GH1 | |  | | growth factor | |  | | 1,048 | | 2,72E-02 | |
| IFNG | |  | | cytokine | |  | | -0,521 | | 2,75E-02 | |
| Tetanospasmin | |  | | chemical - endogenous non-mammalian | |  | |  | | 2,75E-02 | |
| CPT1C | | -0,133 | | enzyme | |  | |  | | 2,75E-02 | |
| PARP14 | | -0,007 | | enzyme | |  | |  | | 2,75E-02 | |
| Fcor | |  | | enzyme | |  | |  | | 2,75E-02 | |
| ganetespib | |  | | chemical drug | |  | |  | | 2,75E-02 | |
| quizartinib | |  | | chemical drug | |  | |  | | 2,75E-02 | |
| MCL1 | | 0,174 | | transporter | |  | |  | | 2,75E-02 | |
| miR-193a-3p (and other miRNAs w/seed ACUGGCC) | |  | | mature microRNA | |  | |  | | 2,75E-02 | |
| PDE3B | | 0,665 | | enzyme | |  | |  | | 2,75E-02 | |
| salinomycin | |  | | chemical - endogenous non-mammalian | |  | |  | | 2,75E-02 | |
| LGALS7/LGALS7B | |  | | other | |  | |  | | 2,75E-02 | |
| VLDLR | | 0,234 | | transporter | |  | |  | | 2,75E-02 | |
| ENTPD1 | | -1,118 | | enzyme | |  | |  | | 2,75E-02 | |
| MAPK6 | | -0,063 | | kinase | |  | |  | | 2,75E-02 | |
| 2-[[9-isopropyl-6-[[4-(2-pyridyl)phenyl]methylamino]purin-2-yl]amino]butan-1-ol | |  | | chemical drug | |  | |  | | 2,75E-02 | |
| CYBA | | -0,478 | | enzyme | |  | |  | | 2,75E-02 | |
| lidocaine | |  | | chemical drug | |  | |  | | 2,75E-02 | |
| SS18-SSX2 | |  | | fusion gene/product | |  | |  | | 2,75E-02 | |
| lamivudine | |  | | chemical drug | |  | |  | | 2,75E-02 | |
| N,N-dimethylarginine | |  | | chemical - endogenous mammalian | |  | |  | | 2,75E-02 | |
| KLF4 | | -0,028 | | transcription regulator | |  | | -1,110 | | 2,78E-02 | |
| DNMT3B | |  | | enzyme | |  | | -0,213 | | 2,79E-02 | |
| Histone h4 | |  | | group | |  | |  | | 2,80E-02 | |
| CNR1 | | 1,718 | | G-protein coupled receptor | |  | | -1,238 | | 2,80E-02 | |
| JAK1/2 | |  | | group | |  | | 0,447 | | 2,82E-02 | |
| mir-181 | |  | | microRNA | |  | | -0,714 | | 2,82E-02 | |
| ARNT | | 0,082 | | transcription regulator | |  | | 0,000 | | 2,83E-02 | |
| lovastatin | |  | | chemical drug | |  | | 0,625 | | 2,90E-02 | |
| choline fenofibrate | |  | | chemical drug | |  | |  | | 2,91E-02 | |
| ruxolitinib | |  | | chemical drug | |  | |  | | 2,91E-02 | |
| miR-22-3p (miRNAs w/seed AGCUGCC) | |  | | mature microRNA | |  | |  | | 2,91E-02 | |
| PRKAR2B | | 0,186 | | kinase | |  | |  | | 2,91E-02 | |
| TAF6 | | 0,058 | | transcription regulator | |  | |  | | 2,91E-02 | |
| epigallocatechin-gallate | |  | | chemical drug | |  | | 1,442 | | 2,91E-02 | |
| Igm | |  | | complex | |  | | 0,747 | | 2,97E-02 | |
| TEAD1 | | 0,065 | | transcription regulator | |  | | 0,277 | | 2,97E-02 | |
| gemfibrozil | |  | | chemical drug | |  | | 0,636 | | 2,97E-02 | |
| PC-SPES | |  | | chemical drug | |  | |  | | 2,98E-02 | |
| IL24 | |  | | cytokine | |  | | -0,912 | | 3,00E-02 | |
| BRD4 | |  | | kinase | |  | | -1,342 | | 3,00E-02 | |
| AKT1 | | -0,121 | | kinase | |  | | 0,742 | | 3,06E-02 | |
| Tnf (family) | |  | | group | |  | | 0,478 | | 3,10E-02 | |
| MSTN | | 0,815 | | growth factor | |  | | -0,840 | | 3,13E-02 | |
| MAOA | | 0,131 | | enzyme | |  | |  | | 3,19E-02 | |
| PTCH1 | | -0,132 | | transmembrane receptor | |  | |  | | 3,19E-02 | |
| chrysotile asbestos | |  | | chemical toxicant | |  | |  | | 3,19E-02 | |
| letrozole | |  | | chemical drug | |  | |  | | 3,19E-02 | |
| epoxomicin | |  | | chemical - protease inhibitor | |  | |  | | 3,19E-02 | |
| deoxynivalenol | |  | | chemical toxicant | |  | |  | | 3,19E-02 | |
| buthionine sulfoximine | |  | | chemical drug | |  | |  | | 3,19E-02 | |
| PRKAA | |  | | group | |  | | -0,958 | | 3,20E-02 | |
| ulipristal acetate | |  | | chemical drug | |  | |  | | 3,25E-02 | |
| WWC1 | | 0,052 | | transcription regulator | |  | |  | | 3,25E-02 | |
| PLCL2 | | -0,175 | | enzyme | |  | |  | | 3,25E-02 | |
| RSPO1 | | 0,000 | | other | |  | |  | | 3,25E-02 | |
| buparlisib | |  | | chemical drug | |  | |  | | 3,25E-02 | |
| miR-451a (and other miRNAs w/seed AACCGUU) | |  | | mature microRNA | |  | |  | | 3,25E-02 | |
| PRAME | |  | | other | |  | |  | | 3,25E-02 | |
| NACC1 | | -0,082 | | transcription regulator | |  | |  | | 3,25E-02 | |
| BAG3 | | -0,136 | | other | |  | |  | | 3,25E-02 | |
| NDP | | 0,059 | | growth factor | |  | |  | | 3,25E-02 | |
| clorgyline | |  | | chemical drug | |  | |  | | 3,25E-02 | |
| quinacrine | |  | | chemical drug | |  | |  | | 3,25E-02 | |
| D-sphingosine | |  | | chemical - endogenous mammalian | |  | |  | | 3,25E-02 | |
| dehydrocostus lactone | |  | | chemical - endogenous non-mammalian | |  | |  | | 3,25E-02 | |
| HOXA9 | | -1,422 | | transcription regulator | |  | | -0,412 | | 3,25E-02 | |
| entinostat | |  | | chemical drug | |  | | 1,732 | | 3,29E-02 | |
| folic acid | |  | | chemical - endogenous mammalian | |  | | 1,342 | | 3,29E-02 | |
| arsenic trioxide | |  | | chemical drug | |  | | -1,443 | | 3,32E-02 | |
| CD40LG | |  | | cytokine | |  | | 0,604 | | 3,32E-02 | |
| fluocinolone acetonide | |  | | chemical drug | | Activated | | 2,000 | | 3,41E-02 | |
| Hsp70 | |  | | group | |  | | 1,364 | | 3,41E-02 | |
| hydrocortisone | |  | | chemical - endogenous mammalian | |  | | 1,937 | | 3,43E-02 | |
| PIK3CA | | -0,010 | | kinase | |  | |  | | 3,46E-02 | |
| CASP3 | | 0,210 | | peptidase | |  | |  | | 3,48E-02 | |
| VCP | | -0,070 | | enzyme | |  | |  | | 3,48E-02 | |
| stearic acid | |  | | chemical - endogenous mammalian | |  | |  | | 3,48E-02 | |
| TLR9 | | -0,518 | | transmembrane receptor | |  | | -0,469 | | 3,58E-02 | |
| ZBTB20 | | 0,102 | | transcription regulator | |  | | -1,000 | | 3,62E-02 | |
| panobinostat | |  | | chemical drug | |  | | 0,685 | | 3,62E-02 | |
| TEAD3 | | 0,042 | | transcription regulator | |  | | 1,000 | | 3,62E-02 | |
| advanced glycation end-products | |  | | chemical - endogenous mammalian | |  | |  | | 3,62E-02 | |
| HIPK2 | |  | | kinase | |  | | -0,714 | | 3,63E-02 | |
| MEF2D | | 0,068 | | transcription regulator | | Activated | | 2,200 | | 3,63E-02 | |
| JQ1 | |  | | chemical reagent | |  | | 0,563 | | 3,77E-02 | |
| (Z,E)-5-(4-ethylbenzylidine)-2-thioxothiazolidin-4-one | |  | | chemical reagent | |  | |  | | 3,78E-02 | |
| (±)-2-hydroxyoleic acid | |  | | chemical drug | |  | |  | | 3,78E-02 | |
| harmine | |  | | chemical - endogenous non-mammalian | |  | |  | | 3,78E-02 | |
| SAR1A | | 0,005 | | enzyme | |  | |  | | 3,78E-02 | |
| SOX5 | | -0,044 | | transcription regulator | |  | |  | | 3,78E-02 | |
| LRAT | |  | | enzyme | |  | |  | | 3,78E-02 | |
| TLE1 | |  | | transcription regulator | |  | |  | | 3,78E-02 | |
| DOCK2 | | -0,273 | | other | |  | |  | | 3,78E-02 | |
| TIA1 | | 0,042 | | other | |  | |  | | 3,78E-02 | |
| PLA2G2E | |  | | enzyme | |  | |  | | 3,78E-02 | |
| HSPA1A/HSPA1B | |  | | enzyme | |  | |  | | 3,78E-02 | |
| cantharidin | |  | | chemical drug | |  | |  | | 3,78E-02 | |
| pterostilbene | |  | | chemical drug | |  | |  | | 3,78E-02 | |
| geranylgeranylacetone | |  | | chemical drug | |  | |  | | 3,78E-02 | |
| clofibric acid | |  | | chemical drug | |  | |  | | 3,78E-02 | |
| selumetinib | |  | | chemical drug | |  | |  | | 3,79E-02 | |
| FTO | | 0,036 | | enzyme | |  | |  | | 3,79E-02 | |
| miR-26a-5p (and other miRNAs w/seed UCAAGUA) | |  | | mature microRNA | |  | |  | | 3,79E-02 | |
| VTN | | 0,121 | | other | |  | |  | | 3,79E-02 | |
| CCNK | | -0,026 | | kinase | |  | |  | | 3,79E-02 | |
| ANGPT2 | | 1,012 | | growth factor | |  | | -0,336 | | 3,79E-02 | |
| ERK1/2 | |  | | group | |  | | -0,064 | | 3,82E-02 | |
| THPO | | -0,318 | | cytokine | |  | | 0,403 | | 3,83E-02 | |
| NS-398 | |  | | chemical reagent | |  | |  | | 3,83E-02 | |
| BAX | | -0,120 | | transporter | |  | |  | | 3,85E-02 | |
| alitretinoin | |  | | chemical drug | |  | | 1,546 | | 3,89E-02 | |
| methylprednisolone | |  | | chemical drug | |  | | 1,249 | | 3,93E-02 | |
| ARID1A | | 0,013 | | transcription regulator | |  | | 1,172 | | 3,98E-02 | |
| LEPR | | 0,142 | | transmembrane receptor | |  | | 1,709 | | 3,99E-02 | |
| ROR2 | | -0,116 | | kinase | |  | |  | | 3,99E-02 | |
| TCR | |  | | complex | |  | | -0,078 | | 4,05E-02 | |
| CD3 group | |  | | group | | Inhibited | | -2,000 | | 4,08E-02 | |
| RTN4 | | 0,102 | | other | |  | | -1,000 | | 4,08E-02 | |
| ROR1 | | -0,056 | | kinase | |  | |  | | 4,10E-02 | |
| mir-193 | |  | | microRNA | |  | |  | | 4,10E-02 | |
| NOSTRIN | | 0,311 | | transcription regulator | |  | |  | | 4,10E-02 | |
| UXT | | -0,057 | | transcription regulator | |  | |  | | 4,10E-02 | |
| SMAD5 | | -0,095 | | transcription regulator | |  | |  | | 4,10E-02 | |
| HDAC2 | | -0,055 | | transcription regulator | |  | | -0,128 | | 4,14E-02 | |
| erlotinib | |  | | chemical drug | |  | | 0,128 | | 4,14E-02 | |
| AICAR | |  | | chemical - endogenous mammalian | |  | | 1,491 | | 4,14E-02 | |
| CCL5 | | 0,000 | | cytokine | |  | | 0,555 | | 4,18E-02 | |
| rottlerin | |  | | chemical drug | | Activated | | 2,216 | | 4,18E-02 | |
| miR-34a-5p (and other miRNAs w/seed GGCAGUG) | |  | | mature microRNA | |  | | 0,272 | | 4,21E-02 | |
| SIRT1 | | 0,008 | | transcription regulator | |  | | -0,894 | | 4,23E-02 | |
| INHA | | 0,741 | | growth factor | |  | | 0,600 | | 4,31E-02 | |
| MAPK3 | | -0,005 | | kinase | | Activated | | 2,449 | | 4,31E-02 | |
| PRL | |  | | cytokine | |  | | -0,974 | | 4,31E-02 | |
| oblimersen | |  | | biologic drug | |  | | -1,982 | | 4,32E-02 | |
| APLN | | -0,083 | | other | |  | | 0,254 | | 4,32E-02 | |
| SFRP1 | | 0,359 | | transmembrane receptor | |  | | -0,655 | | 4,32E-02 | |
| EIF2S1 | | -0,107 | | translation regulator | |  | |  | | 4,32E-02 | |
| U46619 | |  | | chemical reagent | |  | |  | | 4,35E-02 | |
| ginsenoside Rb1 | |  | | chemical - endogenous non-mammalian | |  | |  | | 4,35E-02 | |
| exisulind | |  | | chemical - endogenous mammalian | |  | |  | | 4,35E-02 | |
| mocetinostat | |  | | chemical drug | |  | |  | | 4,35E-02 | |
| Ptprd | |  | | phosphatase | |  | |  | | 4,35E-02 | |
| AMBRA1 | | -0,022 | | other | |  | |  | | 4,35E-02 | |
| FH | | -0,116 | | enzyme | |  | |  | | 4,35E-02 | |
| SURF1 | |  | | enzyme | |  | |  | | 4,35E-02 | |
| STOX1 | | -1,314 | | other | |  | |  | | 4,35E-02 | |
| DNAJC3 | | -0,003 | | other | |  | |  | | 4,35E-02 | |
| COL9A1 | |  | | other | |  | |  | | 4,35E-02 | |
| mir-302 | |  | | microRNA | |  | |  | | 4,35E-02 | |
| RMRP | |  | | other | |  | |  | | 4,35E-02 | |
| AIF1 | |  | | other | |  | |  | | 4,35E-02 | |
| SAT1 | | 0,204 | | enzyme | |  | |  | | 4,35E-02 | |
| ACVR1B | | -0,132 | | kinase | |  | |  | | 4,35E-02 | |
| BMX | | 0,362 | | kinase | |  | |  | | 4,35E-02 | |
| CAMKK2 | | 0,161 | | kinase | |  | |  | | 4,35E-02 | |
| SOCS2 | | 0,064 | | other | |  | |  | | 4,35E-02 | |
| cetrorelix | |  | | biologic drug | |  | |  | | 4,35E-02 | |
| ferric ammonium citrate | |  | | chemical drug | |  | |  | | 4,35E-02 | |
| noscapine | |  | | chemical drug | |  | |  | | 4,35E-02 | |
| pyrimidin-2-one beta-ribofuranoside | |  | | chemical reagent | |  | |  | | 4,35E-02 | |
| IFI16 | |  | | transcription regulator | |  | | -0,152 | | 4,38E-02 | |
| NTRK2 | | 0,306 | | kinase | |  | | -1,961 | | 4,38E-02 | |
| EWSR1-FLI1 | |  | | fusion gene/product | |  | | -1,287 | | 4,38E-02 | |
| SP1 | | 0,017 | | transcription regulator | |  | | -1,321 | | 4,39E-02 | |
| ETV6-RUNX1 | |  | | fusion gene/product | |  | | 1,480 | | 4,43E-02 | |
| FUS | |  | | transcription regulator | |  | |  | | 4,44E-02 | |
| TAB1 | |  | | enzyme | |  | |  | | 4,44E-02 | |
| vitamin K3 | |  | | chemical drug | |  | |  | | 4,44E-02 | |
| hymecromone | |  | | chemical drug | |  | |  | | 4,44E-02 | |
| OGT | | 0,062 | | enzyme | |  | | -1,671 | | 4,47E-02 | |
| kainic acid | |  | | chemical toxicant | |  | | 0,174 | | 4,53E-02 | |
| lenalidomide | |  | | chemical drug | |  | | 0,905 | | 4,55E-02 | |
| BIRC5 | | -0,694 | | other | |  | |  | | 4,57E-02 | |
| RPS15 | | -0,083 | | other | |  | |  | | 4,57E-02 | |
| HIF1A | | 0,004 | | transcription regulator | |  | | -0,953 | | 4,60E-02 | |
| testosterone | |  | | chemical - endogenous mammalian | |  | | -0,180 | | 4,63E-02 | |
| HAVCR1 | |  | | other | |  | | 0,555 | | 4,64E-02 | |
| STAT4 | | -0,340 | | transcription regulator | |  | | 0,594 | | 4,65E-02 | |
| CITED2 | | 0,053 | | transcription regulator | |  | | 0,711 | | 4,65E-02 | |
| PDX1 | | -0,211 | | transcription regulator | |  | | 0,640 | | 4,70E-02 | |
| STAT5A | | 0,051 | | transcription regulator | |  | | 1,213 | | 4,71E-02 | |
| AZ5576 | |  | | chemical drug | |  | |  | | 4,76E-02 | |
| amikacin | |  | | chemical drug | |  | |  | | 4,76E-02 | |
| Clock-Bmal1 | |  | | complex | |  | |  | | 4,76E-02 | |
| KCP | | -0,631 | | other | |  | |  | | 4,76E-02 | |
| Gelonin | |  | | chemical toxicant | |  | |  | | 4,76E-02 | |
| axitinib | |  | | chemical drug | |  | |  | | 4,76E-02 | |
| 1,2-dibromo-3-chloropropane | |  | | chemical toxicant | |  | |  | | 4,76E-02 | |
| dibutylnitrosamine | |  | | chemical toxicant | |  | |  | | 4,76E-02 | |
| AEOL-10150 | |  | | chemical drug | |  | |  | | 4,76E-02 | |
| Mitochondrial complex 1 | |  | | complex | |  | |  | | 4,76E-02 | |
| 2-methoxycinnamaldehyde | |  | | chemical - endogenous non-mammalian | |  | |  | | 4,76E-02 | |
| thymol | |  | | chemical reagent | |  | |  | | 4,76E-02 | |
| Rbx1 | | 0,021 | | enzyme | |  | |  | | 4,76E-02 | |
| EIF4B | | 0,043 | | translation regulator | |  | |  | | 4,76E-02 | |
| FAM168A | | 0,067 | | other | |  | |  | | 4,76E-02 | |
| 4930426D05Rik | |  | | other | |  | |  | | 4,76E-02 | |
| UPK3A | |  | | other | |  | |  | | 4,76E-02 | |
| FBXL12 | | -0,060 | | other | |  | |  | | 4,76E-02 | |
| USP15 | | -0,079 | | peptidase | |  | |  | | 4,76E-02 | |
| ZNF367 | | -0,393 | | transcription regulator | |  | |  | | 4,76E-02 | |
| PCDH9 | | -0,226 | | other | |  | |  | | 4,76E-02 | |
| SLC25A33 | | -0,015 | | transporter | |  | |  | | 4,76E-02 | |
| SRRT | | -0,078 | | other | |  | |  | | 4,76E-02 | |
| PHOSPHO1 | | -2,054 | | enzyme | |  | |  | | 4,76E-02 | |
| HOXD13 | | -0,298 | | transcription regulator | |  | |  | | 4,76E-02 | |
| ECI1 | | -0,000 | | enzyme | |  | |  | | 4,76E-02 | |
| FUT9 | |  | | enzyme | |  | |  | | 4,76E-02 | |
| STAT3/5 | |  | | group | |  | |  | | 4,76E-02 | |
| BCL6 peptide inhibitor | |  | | chemical reagent | |  | |  | | 4,76E-02 | |
| 1-(3-C-ethynylribopentofuranosyl)cytosine | |  | | chemical drug | |  | |  | | 4,76E-02 | |
| abexinostat | |  | | chemical drug | |  | |  | | 4,76E-02 | |
| bucillamine | |  | | chemical drug | |  | |  | | 4,76E-02 | |
| CC 401 | |  | | chemical drug | |  | |  | | 4,76E-02 | |
| CACYBP | | -0,208 | | other | |  | |  | | 4,76E-02 | |
| CIB1 | | -0,016 | | other | |  | |  | | 4,76E-02 | |
| RNMT | | 0,009 | | enzyme | |  | |  | | 4,76E-02 | |
| safflor yellow B | |  | | chemical - endogenous non-mammalian | |  | |  | | 4,76E-02 | |
| NOSIP | | -0,117 | | other | |  | |  | | 4,76E-02 | |
| SH3GLB1 | | 0,003 | | enzyme | |  | |  | | 4,76E-02 | |
| DGKD | | 0,007 | | kinase | |  | |  | | 4,76E-02 | |
| NPBWR1 | | 0,185 | | G-protein coupled receptor | |  | |  | | 4,76E-02 | |
| HLTF | | -0,009 | | transcription regulator | |  | |  | | 4,76E-02 | |
| KIFC1 | | -0,616 | | enzyme | |  | |  | | 4,76E-02 | |
| SYCP3 | | 1,073 | | other | |  | |  | | 4,76E-02 | |
| ELOC | | -0,123 | | transcription regulator | |  | |  | | 4,76E-02 | |
| PMAIP1 | |  | | other | |  | |  | | 4,76E-02 | |
| CCNB1 | | -0,640 | | kinase | |  | |  | | 4,76E-02 | |
| SLC20A1 | | -0,184 | | transporter | |  | |  | | 4,76E-02 | |
| miR-708-5p (and other miRNAs w/seed AGGAGCU) | |  | | mature microRNA | |  | |  | | 4,76E-02 | |
| miR-411-5p (and other miRNAs w/seed AGUAGAC) | |  | | mature microRNA | |  | |  | | 4,76E-02 | |
| mir-663 | |  | | microRNA | |  | |  | | 4,76E-02 | |
| miR-1195 (miRNAs w/seed GAGUUCG) | |  | | mature microRNA | |  | |  | | 4,76E-02 | |
| TAF5L | | -0,057 | | transcription regulator | |  | |  | | 4,76E-02 | |
| PIM | |  | | group | |  | |  | | 4,76E-02 | |
| UBE4B | | 0,093 | | enzyme | |  | |  | | 4,76E-02 | |
| PLIN3 | | -0,141 | | other | |  | |  | | 4,76E-02 | |
| MUC16 | |  | | other | |  | |  | | 4,76E-02 | |
| Dst | |  | | other | |  | |  | | 4,76E-02 | |
| SNF8 | | -0,150 | | enzyme | |  | |  | | 4,76E-02 | |
| KCNJ6 | | -1,146 | | ion channel | |  | |  | | 4,76E-02 | |
| cyclo(iso-Asp-GR)-LLIIKLAKLAKKLAKLAK | |  | | chemical reagent | |  | |  | | 4,76E-02 | |
| THAP12 | | -0,019 | | other | |  | |  | | 4,76E-02 | |
| ING2 | | -0,033 | | transcription regulator | |  | |  | | 4,76E-02 | |
| NAGLU | | 0,166 | | enzyme | |  | |  | | 4,76E-02 | |
| KCNAB2 | | 0,095 | | ion channel | |  | |  | | 4,76E-02 | |
| DMAP1 | | 0,012 | | transcription regulator | |  | |  | | 4,76E-02 | |
| NTSR2 | | -1,164 | | G-protein coupled receptor | |  | |  | | 4,76E-02 | |
| SVIL | | -0,013 | | other | |  | |  | | 4,76E-02 | |
| DBF4B | | -0,265 | | other | |  | |  | | 4,76E-02 | |
| TH | | 1,522 | | enzyme | |  | |  | | 4,76E-02 | |
| CDC23 | | -0,099 | | enzyme | |  | |  | | 4,76E-02 | |
| MSH3 | | 0,059 | | enzyme | |  | |  | | 4,76E-02 | |
| GPBP1 | | -0,048 | | transcription regulator | |  | |  | | 4,76E-02 | |
| CYTH3 | | 0,106 | | other | |  | |  | | 4,76E-02 | |
| MED6 | | -0,079 | | transcription regulator | |  | |  | | 4,76E-02 | |
| troxerutin | |  | | chemical drug | |  | |  | | 4,76E-02 | |
| cyclic des-acyl ghrelin (6-13) | |  | | biologic drug | |  | |  | | 4,76E-02 | |
| PNRC1 | | 0,115 | | other | |  | |  | | 4,76E-02 | |
| IPO9 | | 0,041 | | other | |  | |  | | 4,76E-02 | |
| NEU2 | | -2,018 | | enzyme | |  | |  | | 4,76E-02 | |
| UBE2V1 | | -0,084 | | transcription regulator | |  | |  | | 4,76E-02 | |
| Gm4836 (includes others) | |  | | other | |  | |  | | 4,76E-02 | |
| CRTAP | | 0,022 | | other | |  | |  | | 4,76E-02 | |
| RPLP2 | | -0,003 | | other | |  | |  | | 4,76E-02 | |
| RNF11 | | -0,004 | | enzyme | |  | |  | | 4,76E-02 | |
| OTUD5 | | -0,042 | | enzyme | |  | |  | | 4,76E-02 | |
| ELDR | |  | | other | |  | |  | | 4,76E-02 | |
| nexrutine | |  | | chemical drug | |  | |  | | 4,76E-02 | |
| VNN1 | | -1,676 | | enzyme | |  | |  | | 4,76E-02 | |
| ZFYVE16 | | 0,076 | | other | |  | |  | | 4,76E-02 | |
| 1,5-bis-(dihexyl-N-nitrosoamino)-2,4-dinitrobenzene | |  | | chemical reagent | |  | |  | | 4,76E-02 | |
| mir-434 | |  | | microRNA | |  | |  | | 4,76E-02 | |
| NRIR | |  | | other | |  | |  | | 4,76E-02 | |
| 3830403N18Rik/Xlr | |  | | other | |  | |  | | 4,76E-02 | |
| sorafenib derivative SC-1 | |  | | chemical reagent | |  | |  | | 4,76E-02 | |
| depsipeptide | |  | | chemical - other | |  | |  | | 4,76E-02 | |
| BAI | |  | | chemical drug | |  | |  | | 4,76E-02 | |
| caseamembrin C | |  | | chemical reagent | |  | |  | | 4,76E-02 | |
| S7 | |  | | chemical drug | |  | |  | | 4,76E-02 | |
| SI163 | |  | | chemical drug | |  | |  | | 4,76E-02 | |
| S29 | |  | | chemical drug | |  | |  | | 4,76E-02 | |
| daphnoretin | |  | | chemical - endogenous non-mammalian | |  | |  | | 4,76E-02 | |
| canertinib | |  | | chemical drug | |  | |  | | 4,76E-02 | |
| lonidamine | |  | | chemical drug | |  | |  | | 4,76E-02 | |
| sarpogrelate | |  | | chemical drug | |  | |  | | 4,76E-02 | |
| methiothepin | |  | | chemical drug | |  | |  | | 4,76E-02 | |
| sibutramine | |  | | chemical drug | |  | |  | | 4,76E-02 | |
| PSB-1115 | |  | | chemical reagent | |  | |  | | 4,76E-02 | |
| XK469 | |  | | chemical drug | |  | |  | | 4,76E-02 | |
| foscarnet | |  | | chemical drug | |  | |  | | 4,76E-02 | |
| talipexole | |  | | chemical drug | |  | |  | | 4,76E-02 | |
| miltefosine | |  | | chemical drug | |  | |  | | 4,76E-02 | |
| AN-207 | |  | | chemical toxicant | |  | |  | | 4,76E-02 | |
| cAMP-dependent protein kinase | |  | | complex | |  | |  | | 4,76E-02 | |
| SSR180575 | |  | | chemical drug | |  | |  | | 4,76E-02 | |
| SC144 | |  | | chemical reagent | |  | |  | | 4,76E-02 | |
| KMT2A-MLLT3 | |  | | fusion gene/product | |  | |  | | 4,76E-02 | |
| AZD4573 | |  | | chemical drug | |  | |  | | 4,76E-02 | |
| isoalantolactone | |  | | chemical - endogenous non-mammalian | |  | |  | | 4,76E-02 | |
| swainsonine | |  | | chemical - endogenous non-mammalian | |  | |  | | 4,76E-02 | |
| 3-keto, N-aminoethyl aminocaproyl dihydrocinnamoyl cyclopamine | |  | | chemical reagent | |  | |  | | 4,76E-02 | |
| desflurane | |  | | chemical drug | |  | |  | | 4,76E-02 | |
| 3-O-methylglucose | |  | | chemical reagent | |  | |  | | 4,76E-02 | |
| tibolone | |  | | chemical drug | |  | |  | | 4,76E-02 | |
| MACROH2A1 | | -0,158 | | other | |  | | 0,000 | | 4,78E-02 | |
| Calcineurin A | |  | | group | |  | |  | | 4,78E-02 | |
| GAB2 | | 0,090 | | other | |  | |  | | 4,78E-02 | |
| miR-24-3p (and other miRNAs w/seed GGCUCAG) | |  | | mature microRNA | |  | |  | | 4,78E-02 | |
| IgG | |  | | complex | |  | | 1,854 | | 4,81E-02 | |
| cetuximab | |  | | biologic drug | |  | |  | | 4,94E-02 | |
| THRAP3 | | -0,072 | | transcription regulator | |  | |  | | 4,94E-02 | |
| FOXG1 | |  | | transcription regulator | |  | |  | | 4,94E-02 | |
| RXFP2 | |  | | G-protein coupled receptor | |  | |  | | 4,94E-02 | |
| PDGF (family) | |  | | group | |  | |  | | 4,94E-02 | |
| mir-196 | |  | | microRNA | |  | |  | | 4,94E-02 | |
| GHRHR | |  | | G-protein coupled receptor | |  | |  | | 4,94E-02 | |
| HBP1 | | 0,126 | | transcription regulator | |  | |  | | 4,94E-02 | |
| SP2 | | -0,052 | | transcription regulator | |  | |  | | 4,94E-02 | |
| SERTAD2 | | -0,019 | | transcription regulator | |  | |  | | 4,94E-02 | |
| TOPBP1 | | -0,142 | | other | |  | |  | | 4,94E-02 | |
| lith-O-Asp | |  | | chemical reagent | |  | |  | | 4,94E-02 | |
| L-685,458 | |  | | chemical - protease inhibitor | |  | |  | | 4,94E-02 | |
| hydroxyflutamide | |  | | chemical drug | |  | |  | | 4,94E-02 | |
| NUP98-KDM5A | |  | | fusion gene/product | |  | |  | | 4,94E-02 | |
| NUP98-NSD1 | |  | | fusion gene/product | |  | |  | | 4,94E-02 | |
| sea cucumber body wall meal | |  | | chemical reagent | |  | |  | | 4,94E-02 | |
| NAD+ | |  | | chemical - endogenous mammalian | |  | |  | | 4,94E-02 | |
| SRF | | -0,110 | | transcription regulator | |  | | 1,673 | | 4,96E-02 | |
| SB203580 | |  | | chemical drug | |  | | -0,567 | | 4,96E-02 | |
| CLOCK | | -0,134 | | transcription regulator | |  | | 0,000 | | 4,98E-02 | |
| mir-17 | |  | | microRNA | |  | | -0,418 | | 4,99E-02 | |
| VHL | | 0,051 | | transcription regulator | | Activated | | 2,177 | | 5,29E-02 | |
| SPARC | | -0,003 | | other | | Activated | | 2,414 | | 5,76E-02 | |
| miR-17-5p (and other miRNAs w/seed AAAGUGC) | |  | | mature microRNA | | Inhibited | | -2,170 | | 6,37E-02 | |
| palmitic acid | |  | | chemical - endogenous mammalian | | Inhibited | | -2,173 | | 7,00E-02 | |
| FAS | |  | | transmembrane receptor | |  | | -1,886 | | 7,34E-02 | |
| NLRP3 | | -0,083 | | other | | Activated | | 2,213 | | 8,21E-02 | |
| gentamicin | |  | | chemical drug | |  | | -1,941 | | 8,22E-02 | |
| miR-146a-5p (and other miRNAs w/seed GAGAACU) | |  | | mature microRNA | | Activated | | 2,236 | | 8,79E-02 | |
| mir-133 | |  | | microRNA | | Activated | | 2,000 | | 8,82E-02 | |
| LEF1 | | 0,269 | | transcription regulator | | Activated | | 2,646 | | 9,48E-02 | |
| N-nitro-L-arginine methyl ester | |  | | chemical drug | |  | | 1,863 | | 1,00E-01 | |
| EGF | | 0,849 | | growth factor | |  | | -1,960 | | 1,01E-01 | |
| PPARD | | -0,022 | | ligand-dependent nuclear receptor | |  | | 1,970 | | 1,17E-01 | |
| BCL6 | | 0,030 | | transcription regulator | |  | | -1,901 | | 1,25E-01 | |
| PRMT1 | | -0,142 | | enzyme | |  | | 1,980 | | 1,27E-01 | |
| CEBPA | |  | | transcription regulator | |  | | 1,773 | | 1,39E-01 | |
| IL6R | | -0,194 | | transmembrane receptor | |  | | 1,982 | | 1,58E-01 | |
| PTP4A1 | | -0,057 | | phosphatase | | Inhibited | | -2,216 | | 1,60E-01 | |
| propylthiouracil | |  | | chemical drug | | Inhibited | | -2,449 | | 1,68E-01 | |
| Ifnar | |  | | group | | Inhibited | | -2,000 | | 2,01E-01 | |
| glucocorticoid | |  | | chemical drug | | Activated | | 2,061 | | 2,05E-01 | |
| eicosapentenoic acid | |  | | chemical drug | |  | | 1,982 | | 2,26E-01 | |
| oleic acid | |  | | chemical - endogenous mammalian | | Activated | | 2,213 | | 2,35E-01 | |
| SOX2 | |  | | transcription regulator | |  | | 1,761 | | 2,36E-01 | |
| PIK3CG | | 0,058 | | kinase | | Activated | | 2,000 | | 2,52E-01 | |
| 1-methyl-4-phenyl-1,2,3,6-tetrahydropyridine | |  | | chemical toxicant | | Inhibited | | -2,000 | | 2,52E-01 | |
| IRF8 | | -0,988 | | transcription regulator | |  | | -1,946 | | 2,71E-01 | |
| sulforafan | |  | | chemical drug | | Activated | | 2,000 | | 2,71E-01 | |
| Tcf7 | |  | | transcription regulator | | Activated | | 2,646 | | 2,95E-01 | |
| LEP | |  | | growth factor | | Activated | | 2,809 | | 2,99E-01 | |
| SPI1 | | 0,758 | | transcription regulator | |  | | 1,977 | | 3,01E-01 | |
| TGFB3 | | 0,139 | | growth factor | |  | | 1,919 | | 3,27E-01 | |
| THRB | | 0,222 | | ligand-dependent nuclear receptor | | Activated | | 2,236 | | 3,73E-01 | |
| RICTOR | | 0,107 | | other | | Activated | | 2,138 | | 3,91E-01 | |
| PITX2 | | 0,279 | | transcription regulator | |  | | -1,987 | | 3,93E-01 | |
| MEF2C | | -0,002 | | transcription regulator | |  | | 1,941 | | 4,04E-01 | |
| PTGER4 | | -0,049 | | G-protein coupled receptor | |  | | 1,982 | | 5,25E-01 | |
| P38 MAPK | |  | | group | |  | | 1,937 | | 1,00E00 | |
| SATB1 | | -0,002 | | transcription regulator | |  | | -1,954 | | 1,00E00 | |
| PRDM1 | | -0,172 | | transcription regulator | | Activated | | 2,236 | | 1,00E00 | |
| Melatonin + 4P-PDOT (10^-9^ M) | | | | | | | | | | |  |
| Upstream Regulator | **Expr Log Ratio** | | **Molecule Type** | | **Predicted Activation State** | | **Activation z-score** | | **p-value of overlap** | |  |
| PROKR2 | -0,406 | | G-protein coupled receptor | |  | |  | | 3,77E-04 | |  |
| ISLR |  | | other | |  | |  | | 9,42E-04 | |  |
| MIR99A-LET7C-MIR125B2 |  | | group | |  | |  | | 1,12E-03 | |  |
| MSI2 | 0,004 | | other | |  | |  | | 1,36E-03 | |  |
| YAP1 | -0,017 | | transcription regulator | |  | |  | | 2,04E-03 | |  |
| RPS15 | -0,029 | | other | |  | |  | | 2,90E-03 | |  |
| SUV39H1 | -0,103 | | enzyme | |  | |  | | 5,03E-03 | |  |
| HAS2-AS1 |  | | other | |  | |  | | 5,07E-03 | |  |
| PRPF31 | 0,073 | | other | |  | |  | | 5,07E-03 | |  |
| ADTRP |  | | enzyme | |  | |  | | 5,07E-03 | |  |
| USP39 | 0,001 | | peptidase | |  | |  | | 5,07E-03 | |  |
| PAIP2 | -0,030 | | translation regulator | |  | |  | | 5,07E-03 | |  |
| PRNCR1 |  | | other | |  | |  | | 5,07E-03 | |  |
| UBL5 | -0,188 | | other | |  | |  | | 5,07E-03 | |  |
| LINC02206 |  | | other | |  | |  | | 5,07E-03 | |  |
| RKQ peptide |  | | chemical reagent | |  | |  | | 5,07E-03 | |  |
| TAC peptide |  | | chemical reagent | |  | |  | | 5,07E-03 | |  |
| TDC |  | | chemical reagent | |  | |  | | 5,07E-03 | |  |
| STAT3 inhibitor IX |  | | chemical reagent | |  | |  | | 5,07E-03 | |  |
| mir-101 |  | | microRNA | |  | |  | | 6,02E-03 | |  |
| levodopa |  | | chemical - endogenous mammalian | |  | | 1,000 | | 7,46E-03 | |  |
| miR-30c-5p (and other miRNAs w/seed GUAAACA) |  | | mature microRNA | |  | | 1,948 | | 7,90E-03 | |  |
| CAV1 | -0,049 | | transmembrane receptor | |  | |  | | 7,90E-03 | |  |
| methyl-beta-cyclodextrin |  | | chemical drug | |  | |  | | 8,24E-03 | |  |
| amphetamine |  | | chemical drug | |  | |  | | 9,58E-03 | |  |
| 2,3',4,4',5-pentachlorobiphenyl |  | | chemical toxicant | |  | |  | | 1,01E-02 | |  |
| FBXO31 | -0,038 | | enzyme | |  | |  | | 1,01E-02 | |  |
| FTX |  | | other | |  | |  | | 1,01E-02 | |  |
| PRPF8 | 0,023 | | other | |  | |  | | 1,01E-02 | |  |
| SNF8 | -0,046 | | enzyme | |  | |  | | 1,01E-02 | |  |
| 5-oxo-6-8-11-14-(e,z,z,z)-eicosatetraenoic acid |  | | chemical - endogenous mammalian | |  | |  | | 1,01E-02 | |  |
| NFIA | -0,332 | | transcription regulator | |  | |  | | 1,08E-02 | |  |
| KN 93 |  | | chemical drug | |  | |  | | 1,15E-02 | |  |
| DIO3 |  | | enzyme | |  | | 0,000 | | 1,17E-02 | |  |
| CST5 |  | | other | |  | | 0,447 | | 1,36E-02 | |  |
| PAK1 | -0,031 | | kinase | |  | |  | | 1,36E-02 | |  |
| mycosporine-like amino acid |  | | chemical reagent | |  | |  | | 1,51E-02 | |  |
| hexa-D-arginine |  | | biologic drug | |  | |  | | 1,51E-02 | |  |
| GALNT14 | -0,801 | | enzyme | |  | |  | | 1,51E-02 | |  |
| MSRA | 0,008 | | enzyme | |  | |  | | 1,51E-02 | |  |
| gelatinase |  | | group | |  | |  | | 1,51E-02 | |  |
| ELAVL2 | -0,650 | | other | |  | |  | | 1,51E-02 | |  |
| ALDH1A3 | -0,047 | | enzyme | |  | |  | | 1,51E-02 | |  |
| Ank2 |  | | other | |  | |  | | 1,51E-02 | |  |
| DTNA | -0,079 | | other | |  | |  | | 1,51E-02 | |  |
| INHBC | 0,710 | | growth factor | |  | |  | | 1,51E-02 | |  |
| RPL37 | -0,020 | | other | |  | |  | | 1,51E-02 | |  |
| YY1AP1 |  | | other | |  | |  | | 1,51E-02 | |  |
| SNRNP70 | 0,037 | | other | |  | |  | | 1,51E-02 | |  |
| mecamylamine |  | | chemical drug | |  | |  | | 1,51E-02 | |  |
| GDF9 | -0,141 | | growth factor | |  | |  | | 1,59E-02 | |  |
| streptozocin |  | | chemical drug | |  | | -0,648 | | 1,60E-02 | |  |
| INHA | 0,983 | | growth factor | |  | |  | | 1,68E-02 | |  |
| N-acetyl-D-mannosamine |  | | chemical - endogenous mammalian | |  | |  | | 2,01E-02 | |  |
| SF3B1 | 0,017 | | other | |  | |  | | 2,01E-02 | |  |
| LAMB1 | 0,004 | | other | |  | |  | | 2,01E-02 | |  |
| HAS3 | -0,012 | | enzyme | |  | |  | | 2,01E-02 | |  |
| SEMA3E | -0,187 | | other | |  | |  | | 2,01E-02 | |  |
| D-mannosamine |  | | chemical - endogenous mammalian | |  | |  | | 2,01E-02 | |  |
| GAA | 0,044 | | enzyme | |  | |  | | 2,01E-02 | |  |
| ARNT2 | 0,051 | | transcription regulator | |  | | 0,000 | | 2,09E-02 | |  |
| mir-150 |  | | microRNA | |  | |  | | 2,10E-02 | |  |
| TGFB2 | -0,024 | | growth factor | |  | |  | | 2,18E-02 | |  |
| SIM1 | 0,013 | | transcription regulator | |  | | 0,000 | | 2,40E-02 | |  |
| OLR1 |  | | transmembrane receptor | |  | |  | | 2,47E-02 | |  |
| Adenosine Receptor |  | | group | |  | |  | | 2,51E-02 | |  |
| Hottip |  | | other | |  | |  | | 2,51E-02 | |  |
| FOXN4 |  | | transcription regulator | |  | |  | | 2,51E-02 | |  |
| mir-342 |  | | microRNA | |  | |  | | 2,51E-02 | |  |
| H2AB3 (includes others) |  | | other | |  | |  | | 2,77E-02 | |  |
| mir-30 |  | | microRNA | |  | |  | | 2,77E-02 | |  |
| lithium chloride |  | | chemical drug | |  | |  | | 2,86E-02 | |  |
| HTT | 0,074 | | transcription regulator | |  | |  | | 2,96E-02 | |  |
| norethindrone acetate |  | | chemical drug | |  | |  | | 3,01E-02 | |  |
| mGLUR Group I |  | | group | |  | |  | | 3,01E-02 | |  |
| PIWIL4 | 0,102 | | other | |  | |  | | 3,01E-02 | |  |
| HAS1 |  | | enzyme | |  | |  | | 3,01E-02 | |  |
| miR-342-3p (miRNAs w/seed CUCACAC) |  | | mature microRNA | |  | |  | | 3,01E-02 | |  |
| NUDT21 | -0,012 | | other | |  | |  | | 3,01E-02 | |  |
| RPS20 | -0,072 | | other | |  | |  | | 3,01E-02 | |  |
| enzastaurin |  | | chemical drug | |  | |  | | 3,01E-02 | |  |
| BML-284 |  | | chemical reagent | |  | |  | | 3,01E-02 | |  |
| papaverine |  | | chemical drug | |  | |  | | 3,01E-02 | |  |
| resiniferatoxin |  | | chemical drug | |  | |  | | 3,01E-02 | |  |
| oxamic acid |  | | chemical - endogenous non-mammalian | |  | |  | | 3,01E-02 | |  |
| WNT1 | -0,579 | | cytokine | |  | |  | | 3,03E-02 | |  |
| MFAP5 | -0,302 | | other | |  | |  | | 3,07E-02 | |  |
| WBP2 | 0,043 | | transcription regulator | |  | |  | | 3,14E-02 | |  |
| Z-LLL-CHO |  | | chemical - protease inhibitor | |  | | -0,692 | | 3,37E-02 | |  |
| ADAM10 | -0,027 | | peptidase | |  | |  | | 3,45E-02 | |  |
| CX3CL1 |  | | cytokine | |  | |  | | 3,45E-02 | |  |
| doramapimod |  | | chemical drug | |  | |  | | 3,50E-02 | |  |
| REV1 | 0,043 | | enzyme | |  | |  | | 3,50E-02 | |  |
| COMT | 0,056 | | enzyme | |  | |  | | 3,50E-02 | |  |
| SPTBN4 | 0,236 | | other | |  | |  | | 3,50E-02 | |  |
| PIK3C3 | 0,028 | | kinase | |  | |  | | 3,50E-02 | |  |
| GUCY2F | 0,006 | | kinase | |  | |  | | 3,50E-02 | |  |
| MED13L | -0,014 | | other | |  | |  | | 3,50E-02 | |  |
| GSK 650394 |  | | chemical reagent | |  | |  | | 3,50E-02 | |  |
| ITGB1BP1 | -0,018 | | other | |  | |  | | 3,50E-02 | |  |
| SJN 2511 |  | | chemical drug | |  | |  | | 3,50E-02 | |  |
| prostaglandin D2 |  | | chemical - endogenous mammalian | |  | |  | | 3,84E-02 | |  |
| TNFRSF8 | -3,943 | | transmembrane receptor | |  | |  | | 3,84E-02 | |  |
| TNF | 0,000 | | cytokine | |  | | -0,528 | | 3,85E-02 | |  |
| IL25 |  | | cytokine | |  | |  | | 3,96E-02 | |  |
| fingolimod phosphate |  | | chemical - endogenous mammalian | |  | |  | | 3,99E-02 | |  |
| cosyntropin |  | | biologic drug | |  | |  | | 3,99E-02 | |  |
| PON2 | 0,044 | | enzyme | |  | |  | | 3,99E-02 | |  |
| PDLIM7 |  | | other | |  | |  | | 3,99E-02 | |  |
| RLN1 |  | | other | |  | |  | | 3,99E-02 | |  |
| SOX18 | -0,280 | | transcription regulator | |  | |  | | 3,99E-02 | |  |
| ponesimod |  | | chemical drug | |  | |  | | 3,99E-02 | |  |
| LAMA4 | -0,016 | | enzyme | |  | |  | | 3,99E-02 | |  |
| ARAF | 0,082 | | kinase | |  | |  | | 3,99E-02 | |  |
| SDC2 | 0,021 | | other | |  | |  | | 3,99E-02 | |  |
| LASP1 | 0,003 | | transporter | |  | |  | | 3,99E-02 | |  |
| DKC1 | -0,006 | | enzyme | |  | |  | | 3,99E-02 | |  |
| PCBP1 | -0,021 | | translation regulator | |  | |  | | 3,99E-02 | |  |
| levofloxacin |  | | chemical drug | |  | |  | | 3,99E-02 | |  |
| Histone h4 |  | | group | |  | |  | | 4,03E-02 | |  |
| lovastatin |  | | chemical drug | |  | |  | | 4,10E-02 | |  |
| NEUROG1 |  | | transcription regulator | |  | |  | | 4,31E-02 | |  |
| EGLN1 |  | | enzyme | |  | |  | | 4,43E-02 | |  |
| glucosamine |  | | chemical - endogenous mammalian | |  | |  | | 4,43E-02 | |  |
| 2-oxoadipic acid |  | | chemical - endogenous mammalian | |  | |  | | 4,47E-02 | |  |
| MIR100-LET7A2-MIR125B1 |  | | group | |  | |  | | 4,47E-02 | |  |
| 2-aminoadipic acid |  | | chemical - endogenous mammalian | |  | |  | | 4,47E-02 | |  |
| SOX7 | -0,477 | | transcription regulator | |  | |  | | 4,55E-02 | |  |
| PTGER4 | 0,300 | | G-protein coupled receptor | |  | |  | | 4,58E-02 | |  |
| HNF4A | -0,391 | | transcription regulator | |  | | -1,039 | | 4,70E-02 | |  |
| RBPJ | -0,106 | | transcription regulator | |  | |  | | 4,88E-02 | |  |
| ITGB3 | -0,128 | | transmembrane receptor | |  | |  | | 4,93E-02 | |  |
| 13,14-dihydro-15-ketoprostaglandin D2 |  | | chemical - endogenous mammalian | |  | |  | | 4,96E-02 | |  |
| GFPT1 | -0,055 | | enzyme | |  | |  | | 4,96E-02 | |  |
| NMNAT1 | 0,081 | | enzyme | |  | |  | | 4,96E-02 | |  |
| UGDH | -0,177 | | enzyme | |  | |  | | 4,96E-02 | |  |
| BW 245C |  | | chemical reagent | |  | |  | | 4,96E-02 | |  |
| FGF2 | -0,033 | | growth factor | |  | | -1,969 | | 2,55E-01 | |  |
| TGFB1 | 0,052 | | growth factor | | Inhibited | | -2,396 | | 4,11E-01 | |  |
| MYC | 0,028 | | transcription regulator | |  | | -1,945 | | 4,58E-01 | |  |
| tetradecanoylphorbol acetate |  | | chemical drug | | Inhibited | | -2,195 | | 1,00E00 | |  |

**Supplemental table 8 S8: Canonical pathways affected by the treatments and identified by IPA software**

| Canonical pathways | p-value | Z-score |
| --- | --- | --- |
| Melatonin (10^-7^M) |  |  |
| NAD Biosynthesis from 2-amino-3Carboxymuconate Semialdehyde | 2,48E-02 |  |
| Assembly of RNA Polymerase I Complex | 4,22E-02 |  |
| NAD biosynthesis II (from tryptophan) | 4,56E-02 |  |
| Osteoarthritis Pathway | 5,20E-02 |  |
| Telomerase Signaling | 5,65E-02 |  |
| Melatonin (10^-9^M) |  |  |
| Induction of Apoptosis by HIV1 | 1,92E-04 |  |
| Lipoate Salvage and Modification | 4,39E-03 |  |
| BAG2 Signaling Pathway | 6,04E-3 |  |
| BAG2 Signaling Pathway | 6,04E-03 |  |
| Protein Ubiquitination Pathway | 7,23E-03 |  |
| N-acetyl serotonin (10^-9^M) + melatonin (10^-9^M) |  |  |
| nNOS Signaling in Neurons | 1,54E-03 |  |
| Cell Cycle: G2/M DNA Damage Checkpoint Regulation | 1,84E-03 |  |
| The Visual Cycle | 5,05E-03 |  |
| Molecular Mechanisms of Cancer | 6,18E-03 |  |
| Regulation of Cellular Mechanics by  Calpain Protease | 9,32E-03 |  |
| IIK7 (10^-9^M) + melatonin (10^-9^M) |  |  |
| Kinetochore Metaphase Signaling Pathway | 5,23E-10 |  |
| Mitotic Roles of Polo-Like Kinase | 2,55E-08 |  |
| Cell Cycle: G2/M DNA Damage Checkpoint  Regulation | 1,46E-07 |  |
| GADD45 Signaling | 3,84E-06 |  |
| Hereditary Breast Cancer Signaling | 4,36E-06 |  |
| S26131 (10^-9^M) + melatonin (10^-9^M) |  |  |
| PTEN Signaling | 3,74E-03 |  |
| Semaphorin Neuronal Repulsive Signaling  Pathway | 3,84E-03 |  |
| Ephrin A Signaling | 4,07E-03 |  |
| EIF2 Signaling | 4,48E-03 |  |
| CSDE1 Signaling Pathway | 6,65E-03 |  |
| 4P-PDOT (10^-9^M) + melatonin (10^-9^M) |  |  |
| Spermine Biosynthesis | 9,59E-03 |  |
| ATM Signaling | 1,15E-02 |  |
| Oxidized GTP and dGTP Detoxification | 1,91E-02 |  |
| G-Protein Coupled Receptor Signaling | 2,00E-02 |  |
| cAMP-mediated signaling | 2,62E-02 |  |
